# Thermodynamic model of gene regulation for the Or59b olfactory receptor in *Drosophila*

**DOI:** 10.1371/journal.pcbi.1006709

**Published:** 2019-01-17

**Authors:** Alejandra González, Shadi Jafari, Alberto Zenere, Mattias Alenius, Claudio Altafini

**Affiliations:** 1 Department of Electrical Engineering, Linköping University, Linköping, Sweden; 2 Department of Clinical and Experimental Medicine, Linköping University, Linköping, Sweden; University of Illinois at Urbana-Champaign, UNITED STATES

## Abstract

Complex eukaryotic promoters normally contain multiple cis-regulatory sequences for different transcription factors (TFs). The binding patterns of the TFs to these sites, as well as the way the TFs interact with each other and with the RNA polymerase (RNAp), lead to combinatorial problems rarely understood in detail, especially under varying epigenetic conditions. The aim of this paper is to build a model describing how the main regulatory cluster of the olfactory receptor Or59b drives transcription of this gene in *Drosophila*. The cluster-driven expression of this gene is represented as the equilibrium probability of RNAp being bound to the promoter region, using a statistical thermodynamic approach. The RNAp equilibrium probability is computed in terms of the occupancy probabilities of the single TFs of the cluster to the corresponding binding sites, and of the interaction rules among TFs and RNAp, using experimental data of Or59b expression to tune the model parameters. The model reproduces correctly the changes in RNAp binding probability induced by various mutation of specific sites and epigenetic modifications. Some of its predictions have also been validated in novel experiments.

## Author summary

The paper proposes and validates experimentally a model for the fine-graded regulation of a gene, called Or59b, coding for an olfactory receptor in *Drosophila*. The model is based on statistical thermodynamical theory, theory that so far has been mostly used for prokaryotes. In order to apply it to our more complex eukaryotic system, we have performed a large number of “perturbative” *in vivo* experiments (mutations, knockdown, knockout, epigenetic conditions) meant to unravel the regulatory rules by which the Or59b main regulatory cluster drives gene expression in as much detail as possible. We make use of the knowledge of the Or59b cis-regulatory module acquired in this way to set up the model and to identify its parameters. The model predictions are then tested experimentally in new epigenetic conditions. These new experiments validate the model behavior and confirm its predictive power.

## Introduction

The variety of ways in which the information of the genetic code is expressed in different multicellular organisms depends upon a broad spectrum of regulatory mechanisms. These regulatory mechanisms determine which of the genes are “turned on” and which are “turned off” under specific sets of circumstances, at any given time, and thereby control gene expression. They are also the reason why some genes are expressed in only special types of cells, instead of being expressed in every cell of an organism [1]. Gene promoters contain specific motifs where transcription factors (TFs) can bind, allowing them to enhance or inhibit transcription in response to intracellular or extracellular signals. However, the action of a combination of TFs on their respective motifs is by itself not enough to explain the patterns of gene expression and the spatial restriction needed to explain cell-specific gene regulation [2, 3]. Auxiliary mechanisms like synergistic and competitive effects, cis-regulatory modules, TF isoforms, splicing variants and chromatin state are necessary to determine the regulatory code and the spatially restricted expression [1, 3–6]. As the regulatory mechanisms are all interlaced, the combinatorial complexity rapidly grows with an increasing intricate regulation, and with it the number of experiments that must be performed to get a complete picture of the regulatory process. For eukaryotes, capturing such complex mechanisms of transcriptional regulation in a model is a daunting challenge: only a few gene regulations have been dissected in detail and the resulting models validated experimentally (a classical example being the segmentation network in the *Drosophila* embryo [7–11]).

For prokaryotes, one of the approaches most frequently used to model transcriptional regulation is based on statistical thermodynamics [12–16]. Thermodynamic models use statistical mechanics to compute the level of gene expression by means of the equilibrium probability that an RNA polymerase (RNAp) is bound to the promoter of interest. They are based on the assumption that the two are proportional [17]. The probability of RNAp binding at the specific promoter is obtained from the set of probabilities of promoter occupancy in the various possible configuration states, probabilities which are themselves calculated as functions of the binding affinities of the TSs, of their interactions (cooperative allosteric effects, short-range repression, etc.) and of their interactions with the RNAp in equilibrium conditions. When we try to use thermodynamical models for describing gene regulation in eukaryotes, the picture becomes significantly more complex, not only because the combinatorial regulation due to the multiple binding sites scales in size, but also, and more importantly, because of the role played by chromatin [18].

One of the most studied gene regulatory processes in any multi cellular organism is the monogenic expression of odorant receptors (ORs) in the olfactory system. The olfactory sensory neurons (OSNs) choose to express a single OR from a large gene repertoire in the genome. The specific OR determines the identity and function of the OSN, and the neurons that express the same receptor project their axons to one glomerulus in the brain, creating a functional class [19].

The monogenic OR expression is conserved from *Drosophila* to mouse and humans. A wealth of experiments has explored the regulatory mechanisms that secure single OR expression. In vertebrates, the regulation is based on changes in chromatin state. During OSN development, ORs are covered with heterochromatin and restricted opening of the chromatin induces expression of one OR allele. OR activity on the neuronal surface induces a complex feedback loop that decreases the probability of chromatin opening. This choice-like model can predict the monogenic OR expression but the expression is spatially restricted in a nonrandom pattern. The process that directs the choice is not well understood. In the smaller and not so numerically complex *Drosophila* olfactory system, 61 compared to 1400 ORs in mouse, genetic screens and bioinformatic studies have proposed that the monogenic expression is based on TF combinations and cis-regulatory structures that regulate OR expression in a nonrandom predetermined process. However, the expression of TFs is not restricted to the OSNs that express the regulated ORs and the motifs that the TFs bind are frequent in the genome, suggesting that TF combinatorialism is not the single mechanism that generates spatially restricted OR expression in *Drosophila*.

**Fig 1:**
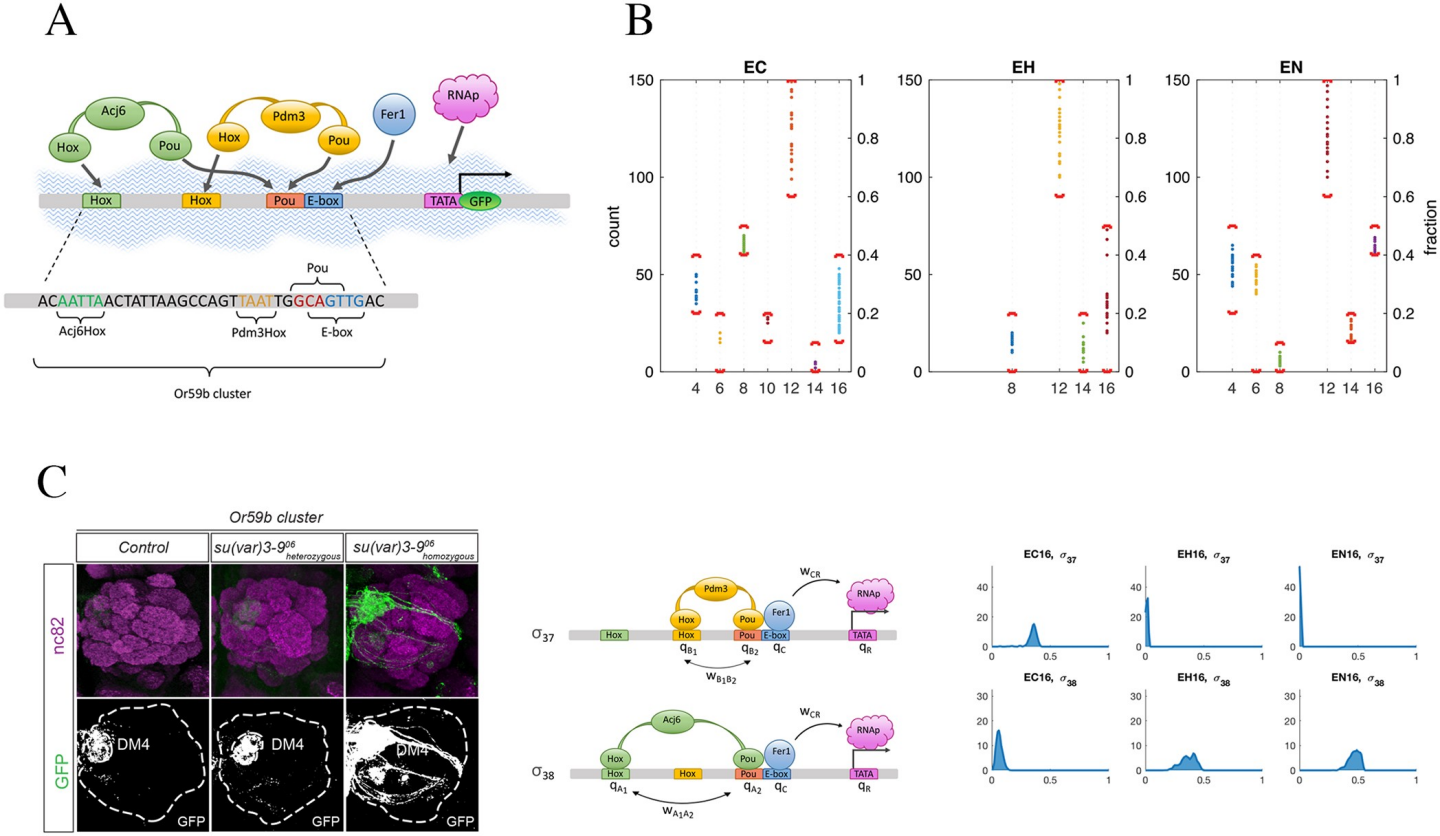
(A): Sketch of the Or59b cluster and TFs involved in the regulation. (B): Experimental countings of the number of GFP-expressing OSNs in the DM4 glomerulus, see Table A of S1 Text for more details. The left axis gives the absolute count, the right axis the normalized value. In the horizontal axis, the experiments are listed as reported in Table 1. For each experiment, the red brackets denote the intervals [lower bound, upper bound], reported also in Table 1. (C): Left panel: whole-mount brain staining showing the expression of GFP driven by the intact Or59b cluster (row E16 in Table 1). The upper row shows synaptic neuropil regions labeled with the presynaptic marker nc82 (magenta). GFP is shown in the lower row. In this paper, only the DM4 glomerulus is of relevance for Or59b expression. The leftmost staining corresponds to normal chromatin (case EC16 of Table 1), the middle one to heterozygous su(var)3-9 mutant (case EH16 of Table 1) and the right one to homozygous su(var)3-9 mutant (case EN16 of Table 1). Middle panel: the two configuration states contributing the most to expression, as suggested by our model: *σ*_37_ and *σ*_38_. See Figs. A-B of S1 Text for a list of all configurations. Right panel: the distributions of the probabilities *P*(*σ*_37_) and *P*(*σ*_38_). When passing from normal chromatin to su(var)3-9 mutants, the first decreases and the second increases.

We have previously genetically investigated the mechanisms behind monogenic Or59b expression in *Drosophila*. We generated an in vivo qualitative description of the regulation events that drive OR59b expression, which was derived from a large set of experiments. Genetic screens revealed that Or59b expression is driven by three TFs: Acj6, Fer1 and Pdm3. Acj6 and Pdm3 are Pou-Homeobox proteins. They have two subunits which each recognizes one of two distinct DNA core motifs (and their variants), called Homeobox domain (AATTA [20, 21]) and Pou domain (TGCAA/T [22, 23]), and have been shown to specify a subset of *Drosophila* ORs [21, 24, 25]. Fer1 is a basic helix-loop-helix protein (bHLH) and binds variations of a core sequence called Ebox motif (CAGCTG). Bioinformatic analysis revealed that binding motifs for the three TFs exist in a cluster directly upstream the promoter region, see Fig 1(A). Our previous genetic experiments demonstrate that the cluster of motives acts as a mini enhancer and is sufficient to drive expression to the Or59b OSN class. Although all four motives in the cluster are short and not consensus, the experiments demonstrate that they are required and that the short-lived TF binding is sufficient to induce expression. Extensive mutation analysis suggests a model where the two Pou-Homeobox proteins Acj6 and Pdm3 open chromatin and the basic helix-loop-helix protein Fer1 induces expression. A competition in between the opening factors and Fer1 limits the expression. Local cooperative interactions between Fer1 in the enhancer and in the vicinity stabilize the expression. The genetic study revealed that the interaction between TFs and chromatin is complex. The chromatin temporarily opens when methyltransferases trimethylate the histones, and this is likely done by means of a complex that methyltransferase forms with Acj6 or Pdm3.

Here, we show that statistical thermodynamical theory provides a suitable framework for a mathematical model which is broader in scope than previously proposed qualitative models and which can describe the Or59b cluster-driven expression regulation in a quantitative manner.

Even though microscopically a very fast chain of dynamical events lead to Fer1 binding (TFs bind Homeobox and Pou domains, temporarily open the chromatin, detach and let Fer1 bind Ebox), in our model the cause-effect interaction of Acj6 or Pdm3 with Fer1 is described in a static way, as usually done in equilibrium models. For the same reason, and to keep the model to a treatable size, the temporary chromatin remodeling associated to binding/unbinding events is not described explicitly.

The mathematical framework is built assembling our in vivo experimental evidence on the regulation of the Or59b gene. The previous demonstrated regulatory interactions can be arranged in 48 different configurations states, denoted *σ*_*k*_, *k* = 1, …, 48, shown in Figures A-B of S1 Text. To each of these states is associated a non normalized probability whose sum gives the total partition function of the system. In turn, this can be used to compute the probability of RNAp binding, hereafter denoted $P_{\text{binding}}^{\text{Or}59b}\left(R-\text{TATAbox}\right)$, see Methods and S1 Text for the details. In our equilibrium model, $P_{\text{binding}}^{\text{Or}59b}\left(R-\text{TATAbox}\right)$ can be identified with the observable of the system, i.e., with the gene expression driven by the Or59b cluster, measured through a GFP fused to the TATA box.

As an example of application of our thermodynamical model, we show in the paper that it can correctly predict the regulation of the Or59b cluster in presence of an altered chromatin state, induced by a homozygous (i.e., null) mutation of su(var)3-9, the enzyme that trimethylates H3K9. The model is fitted based on experiments performed in normal chromatin conditions and in presence of heterozygous (i.e., single-allele) mutation in su(var)3-9. We reasoned that if the heterozygous su(var)3-9 mutant has the effect of rendering the DNA more accessible to TFs (because of the decreased H3K9 trymethylation), a homozygous su(var)3-9 mutant ought to render this process more marked. In fact, this prediction of the model is validated in our new experiments. The main suggestion we get is that a chromatin change is likely to have a significant impact in the regulation of OR expression also in *Drosophila*.

## Results

In order to investigate how the Or59b cluster regulates expression and how the TFs binding generates robust class-specific OR expression, a set of experiments involving mutant species and sites, altered TF concentration, and trimethylation of the chromatin, was performed in [26], see Table 1 and Table A of S1 Text for a summary.

**Table 1:** Truth table of the expression patterns of the Or59b cluster experiments. In the four left columns the mutation table for the cluster motifs Acj6Hox, Pdm3Hox, Pou and Ebox is shown: 0 corresponds to mutated motif and 1 to unaltered motif. The 3 rightmost columns represent the expression driven by the Or59b cluster in Or59b receptors, in our model identified with $P_{\text{binding}}^{\text{Or}59b}\left(R-\text{TATAbox}\right)$. Values are between 0 (total loss) and 1 (very strong expression), see also Fig 1(B) and Table A of S1 Text. The 3 columns correspond to chromatin in its normal state (“closed”, column C), heterozygous mutation of su(var)3-9 (“open”, column H) and homozygous mutation of su(var)3-9 (“more open”, column N). Yellow cells represent configurations which have beed directly experimented in [26], green cells are configurations tested in an indirect way in [26], orange and blue cells are novel direct and indirect experiments. Gray cells correspond to experiments with mutated Ebox, which can all be marked as total loss. When a direct/indirect experiment is missing the cell is left white. The ranges [*ℓ*, *u*] = [upper bound, lower bound] are given according to our quantification of the GFP reporter fused to the TATA box. More details of this quantification are given in Table A of S1 Text. For E8, E12, E14 and E16, GFP expression on selected flies is shown in Fig 5(A)–5(C), and in Fig 1(C). For missing experiments a maximal range is chosen, i.e., [*ℓ*, *u*] = [0, 1] (except for E2 which always leads to loss of expression).

Code	Acj6Hox	Pdm3Hox	Pou	Ebox	Expression driven by the Or59b cluster, and [*ℓ*, *u*]		
					C normal chromatin state (Data from [26])	H heterozygous su(var)3-9 mutant (Data from [26])	N homozygous su(var)3-9 mutant
E1	0	0	0	0	[0, 0.1]	[0, 0.1]	[0, 0.1]
E2	0	0	0	1	[0, 0.1]	[0, 0.1]	[0, 0.1]
E3	0	0	1	0	[0, 0.1]	[0, 0.1]	[0, 0.1]
E4	0	0	1	1	[0.2, 0.4]	[0, 1]	[0.2, 0.5]
E5	0	1	0	0	[0, 0.1]	[0, 0.1]	[0, 0.1]
E6	0	1	0	1	[0, 0.2]	[0, 1]	[0, 0.4]
E7	0	1	1	0	[0, 0.1]	[0, 0.1]	[0, 0.1]
E8	0	1	1	1	[0.4, 0.5]	[0, 0.2]	[0, 0.1]
E9	1	0	0	0	[0, 0.1]	[0, 0.1]	[0, 0.1]
E10	1	0	0	1	[0.1, 0.2]	[0, 1]	[0, 1]
E11		0		0	[0, 0.1]	[0, 0.1]	[0, 0.1]
E12	1	0	1	1	[0.6, 1]	[0.6, 1]	[0.6, 1]
E13	1	1	0	0	[0, 0.1]	[0, 0.1]	[0, 0.1]
E14	1	1	0	1	[0, 0.1]	[0, 0.2]	[0.1, 0.2]
E15	1	1	1	0	[0, 0.1]	[0, 0.1]	[0, 0.1]
E16	1	1	1	1	[0.1, 0.4]	[0, 0.5]	[0.4, 0.5]

For the Or59b cluster, see Fig 1(A), each of the 4 binding motifs can be mutated or be kept unchanged, which generates 2^4^ possibilities represented as the rows of a truth table in Table 1 and Table A of S1 Text. In these tables, mutated motifs in the cluster take the value 0, while 1 accounts for non-mutated motifs. Furthermore, the chromatin can be in its normal state (“closed”, column C in Table 1 and Table A of S1 Text), or in an altered state induced by heterozygous mutant su(var)3-9 (“open”, column H in Table 1 and Table A of S1 Text) or by homozygous mutant su(var)3-9 (“more open”, column N in Table 1 and Table A of S1 Text).

The empirical observable of the system is the number of GFP-expressing OSNs in the whole-mount brain stainings collected for the various mutant combinations, as reported in Table A of S1 Text and Fig 1(B). Only expression of OSNs projected on the DM4 glomerulus is considered. Ectopic expression is disregarded throughout the paper. In Table 1 this experimental evidence is quantified into values between 0 (total loss) and 1 (very strong expression) by normalizing the countings of GFP-expressing OSNs with respect to the maximum of such counts (i.e., 150 OSNs). After this normalization, for each combination of mutants (and each chromatin state) we obtain an interval [*ℓ*, *u*], reported in Table 1.

Combining the binary values of the 4 binding motifs with the 3 chromatin states, we obtain 16 × 3 = 48 possible different experiments (not to be confused with the 48 configuration states *σ*_*k*_). For those combinations for which experimental evidence is available, the resulting expression pattern is given in Table 1.

### Experimental results in normal chromatin state (column C)

Let us briefly recapitulate the results of the experiments of [26] for the normal chromatin state (column C in Table 1). GFP expression driven by the intact Or59b cluster (row E16 in Table 1 and Table A of S1 Text) corresponds to an expression similar to that of the wild-type fly. Mutation of the Ebox motif (row E15) caused total loss of expression, thus indicating that bHLH proteins are needed to activate transcription. From this and related experiments [26], we can infer that all odd rows in Table 1 (shown in gray) correspond to total loss. Mutation of the Pou motif (E14) resulted in near-loss of expression, whereas mutation of Acj6Hox resulted in an expression slightly higher than in the intact Or59b cluster (i.e., expression in EC8 slightly higher that in EC16, see Table 1), and mutation of Pdm3Hox in a very strong expression (i.e., expression in EC12 much stronger than in EC16).

Motifs that have been mutated result in much lower binding strength, which means that rarely a TF can bind to them. A similar effect (decreased likelihood of binding) can be obtained reducing the concentration of the TF, see Eq (1). For the purpose of compiling our truth table, experiments with low TF expression and experiments with mutation of a binding site are treated equivalently (the fact that Or59b cluster contains a single copy of each site makes this association possible). In particular we considered an experiment with knockout of Acj6 (Acj6^6^ males) in conjunction with Pdm3Hox mutation as a proxy for a double Homeobox mutation (Acj6Hox + Pdm3Hox, row E4 in Table 1); an experiment with Acj6^6^ males and mutated Pou as a double mutation Acj6Hox + Pou (row E6); and an experiment with knockdown of Pdm3 (Pdm3-IR) and Pou mutation as a double mutation Pdm3Hox + Pou (row E10), see [26] and S1 Text for the details of these experiments.

### Experimental results in heterozygous su(var)3-9 mutant (column H)

The heterozygous mutation of su(var)3-9 combined with mutation of the specific binding sites produced a different set of expression patterns with respect to the normal chromatin state, reviewed in column H of Table 1 and Fig 1(B). In particular, in a heterozygous mutant su(var)3-9 background, the result of mutating the Acj6Hox motif (E8) was to weaken the expression with respect to the normal chromatin state, while instead mutation of Pdm3Hox (E12) did not result in any appreciable difference, suggesting that the epigenetic state influences the action of these two TF in different ways. Moreover, when only Pou was mutated (E14), a weakly rescued expression took the place of near-complete loss. The mutation of Ebox in this context caused no difference, leading to total loss of expression as before. No information is available for the indirect experiments (rows E4, E6, E10). Notice further (see Table A of S1 Text) how in presence of heterozygous mutation of su(var)3-9 different replicates for the intact cluster case (row E16) produced widely different results, adding to the uncertainty of the system (and of our model).

### Model fitting for columns C and H

The columns C and H were used to fit numerical values to the parameters of our model. The details of the model are described in the Methods section and S1 Text. The binding energies *q*_*j*_, the cooperative and competitive interaction coefficients *w*_*jn*_, and the epigenetic factors *h*_*m*_ are the tuning variables of the model. For the parameter fitting, suitable ranges of values with biological significance and coherency constraints have been imposed (listed in Tables 2, 3 and 4). Random search in the resulting parameter space is then performed as described in the Methods. Reproducing the expression intervals of all the experiments of these two columns in our model is already a challenging task. In particular, it appears to be impossible to fit simultaneously the two columns C and H with identical epigenetic parameters, meaning that changes due to chromatin state must be explicitly incorporated in the model. We therefore assume that the epigenetic parameters *h*_*m*_ can vary passing from normal chromatin state to heterozygous su(var)3-9 mutant, while the parameters describing the binding strengths, *q*_*j*_, and the molecular interactions, *w*_*jn*_, remain constant across all epigenetic conditions. The fitted values for the parameters are reported in Fig. C of S1 Text and in Table 4.

**Table 2:** Binding parameters: Names, meaning and numerical ranges. Parameters describing the TF-DNA bindings used in the model. In the cases marked with *, the extra constraint $q_{A}=q_{A_{1}}q_{A_{2}}$ (or $q_{B}=q_{B_{1}}q_{B_{2}}$) is imposed on the numerical value of the parameters.

Name	Meaning	Numerical range
*q*_*R*_	$\frac{P_{\text{binding}}(R-\text{TATAbox})}{1-P_{\text{binding}}(R-\text{TATAbox})}$	(0.002–0.03)
*q*_*A*_	$q_{A}=q_{A_{1}}q_{A_{2}}$	(0.1–2500)
$q_{A_{1}}$	$\frac{P_{\text{binding}}(A_{1}-{\text{Hox}}_{A_{1}})}{1-P_{\text{binding}}(A_{1}-{\text{Hox}}_{A_{1}})}$	(0.1–2500)*
$q_{A_{2}}$	$\frac{P_{\text{binding}}(A_{2}-\text{Pou})}{1-P_{\text{binding}}(A_{2}-\text{Pou})}$	(0.1–2500) *
*q*_*B*_	$q_{B}=q_{B_{1}}q_{B_{2}}$	(0.1–2500)
$q_{B_{1}}$	$\frac{P_{\text{binding}}(B_{1}-{\text{Hox}}_{B_{1}})}{1-P_{\text{binding}}(B_{1}-{\text{Hox}}_{B_{1}})}$	(0.1–2500) *
$q_{B_{2}}$	$\frac{P_{\text{binding}}(B_{2}-\text{Pou})}{1-P_{\text{binding}}(B_{2}-\text{Pou})}$	(0.1–2500) *
*q*_*C*_	$\frac{P_{\text{binding}}(C-\text{Ebox})}{1-P_{\text{binding}}(C-\text{Ebox})}$	(0.1–2500)

**Table 3:** Interaction parameters: Names, meaning and numerical ranges. Parameters describing TF-TF and TF-RNAp interactions in the model.

Name	Meaning	Numerical range
$w_{A_{1}A_{2}}$	Cooperativity coefficient for double binding of Acj6	(10–100)
$w_{B_{1}B_{2}}$	Cooperativity coefficient for double binding of Pdm3	(10–100)
$w_{A_{1}B_{2}}$	Competitivity coefficient for Acj6 bound to Hox and Pdm3 bound to Pou	(0.0002–0.001)
$w_{A_{2}B_{1}}$	Competitivity coefficient for Pdm3 bound to Hox and Acj6 bound to Pou	(0.0002–0.001)
*w*_*CP*_	Cooperativity coefficient between Fer1 bound to Ebox and RNAp	(30–100)

**Table 4:** Epigenetic parameters: Names, meanings and numerical values. Parameters describing the epigenetic factors included in the model. The values represent the mean of a normal distribution of standard deviation equal to mean/10, see Fig. D of S1 Text.

Name	Meaning	Mean value		
		C	H	N
*h*_1_	Effect on Fer1 bound to Ebox, when neither Acj6 nor Pdm3 is bound to the entire cluster	1	0.9	0.8
*h*_2_	Effect on Fer1 bound to Ebox, when neither Acj6 nor Pdm3 is bound to the Pou domain, but at least one of them is bound to its Homeobox domain	0.00007	0.00008	0.0001
*h*_3_	Extra competition between Fer1 bound to Ebox and Acj6 or Pdm3 bound to the Pou domain	0.0002	0.00035	0.0005
*h*_*A*_	Altered effect of the cooperativity coefficient $w_{A_{1}A_{2}}$ on Fer1 binding to Ebox	30	100	150
*h*_*B*_	Altered effect of the cooperativity coefficient $w_{B_{1}B_{2}}$ on Fer1 binding to Ebox	5	0.1	0.05

**Fig 2:**
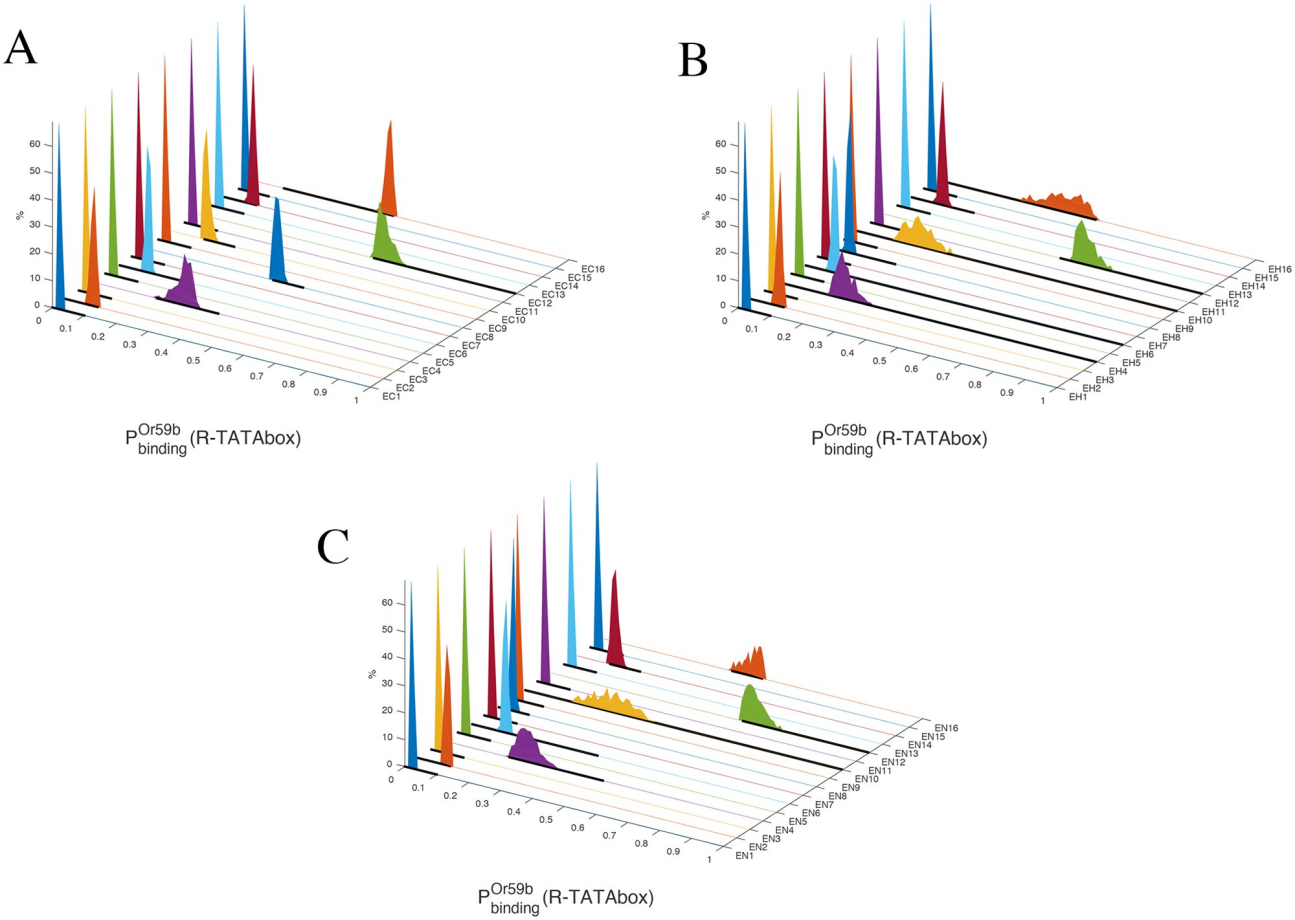
(A): Probability distribution of RNAp binding (i.e., $P_{\text{binding}}^{\text{Or}59b}\left(R-\text{TATAbox}\right)$) for the normal (“closed”) chromatin case (column C of Table 1) in the 16 mutations of the truth table (Table 1). The horizontal black lines represent the admissible expression intervals of the gene, as reported in Table 1 and Fig 1(B). The histograms show only the samples which respect all constraints. (B): $P_{\text{binding}}^{\text{Or}59b}\left(R-\text{TATAbox}\right)$ for the heterozygous mutant su(var)3-9 “open chromatin” case (column H of Table 1). (C): $P_{\text{binding}}^{\text{Or}59b}\left(R-\text{TATAbox}\right)$ for the homozygous mutant su(var)3-9 “more open chromatin” case (column N of Table 1). See also Fig 3 for a specific sample realization from these histograms.

All five epigenetic parameters *h*_*m*_ must vary in order to describe the expression changes when passing from C to H, see Table 4 and Fig. D of S1 Text. Even after tuning *h*_*m*_ as best as we could, only a small fraction (around 0.5%) of the (filtered, see Methods) samples satisfies all constraints imposed on the 13 parameters *q*_*j*_ and *w*_*jn*_ of the model and at the same time fits all the intervals of expression of the experiments (listed in Table 1). See Fig 2(A) and 2(B) for the distribution of Or59b expression values predicted by the model (i.e., the probability distribution of RNAp binding $P_{\text{binding}}^{\text{Or}59b}\left(R-\text{TATAbox}\right)$, see Methods) in the 16 rows of the truth table in columns C and H.

### Validation: Experimental results in homozygous su(var)3-9 mutant (column N)

In order to validate both the pattern of expression observed in [26] and our model predictions, we performed new experiments in homozygous mutant su(var)3-9 background (column N in Table 1 and Table A of S1 Text). The rationale of this choice is that we expect the chromatin to be “more open” than in the heterozygous mutant su(var)3-9 case, hence the trend established when passing from column C to H in Table 1 should continue and become more pronounced in column N. In fact, if we look at the single mutant rows E8, E12 and E14, we observe that indeed the new experiments confirm this hypothesis: for E8 the expression is weakened even further, for E12 it remains essentially unchanged (a very strong expression), while for E14 it grows, see Fig 1(B). An expression stronger than in normal chromatin background is also obtained for the intact cluster case (E16). The two indirect experiments which we could perform (Acj6^6^ males + Pdm3Hox mutation, here identified with E4, and Acj6^6^ males + Pou mutation, identified with E6) both seem to indicate a higher expression than in normal chromatin, although the data also have a higher variance.

All these results are coherent with our interpretation of homozygous su(var)3-9 mutants as “more open” chromatin states, in which the promoter region is generally more accessible and transcription generally favored.

### Model validation, up to epigenetic retuning

To validate the model predictions we keep the same values of the *q*_*j*_ and *w*_*jn*_ parameters computed for the columns C and H, and allow variations only in the epigenetic parameters *h*_*m*_, but respecting the trend established in passing from column C to H: *h*_2_, *h*_3_ and *h*_*A*_ must increase, while *h*_1_ and *h*_*B*_ must decrease, see Table 4. By properly tuning the values of *h*_*m*_, the model is indeed able to reproduce the entire set of experiments of our truth table, in the sense that $P_{\text{binding}}^{\text{Or}59b}(R-\text{TATAbox})$ is within the empirical [lower bound, upper bound] intervals established in Table 1 for all cases, see Fig 2(C). After retuning of the epigenetic parameters, the fraction of samples fitting all experimental data is still in the order of 0.5% of the number of (filtered) samples.

### Analysis of the parameter fitting

**Fig 3:**
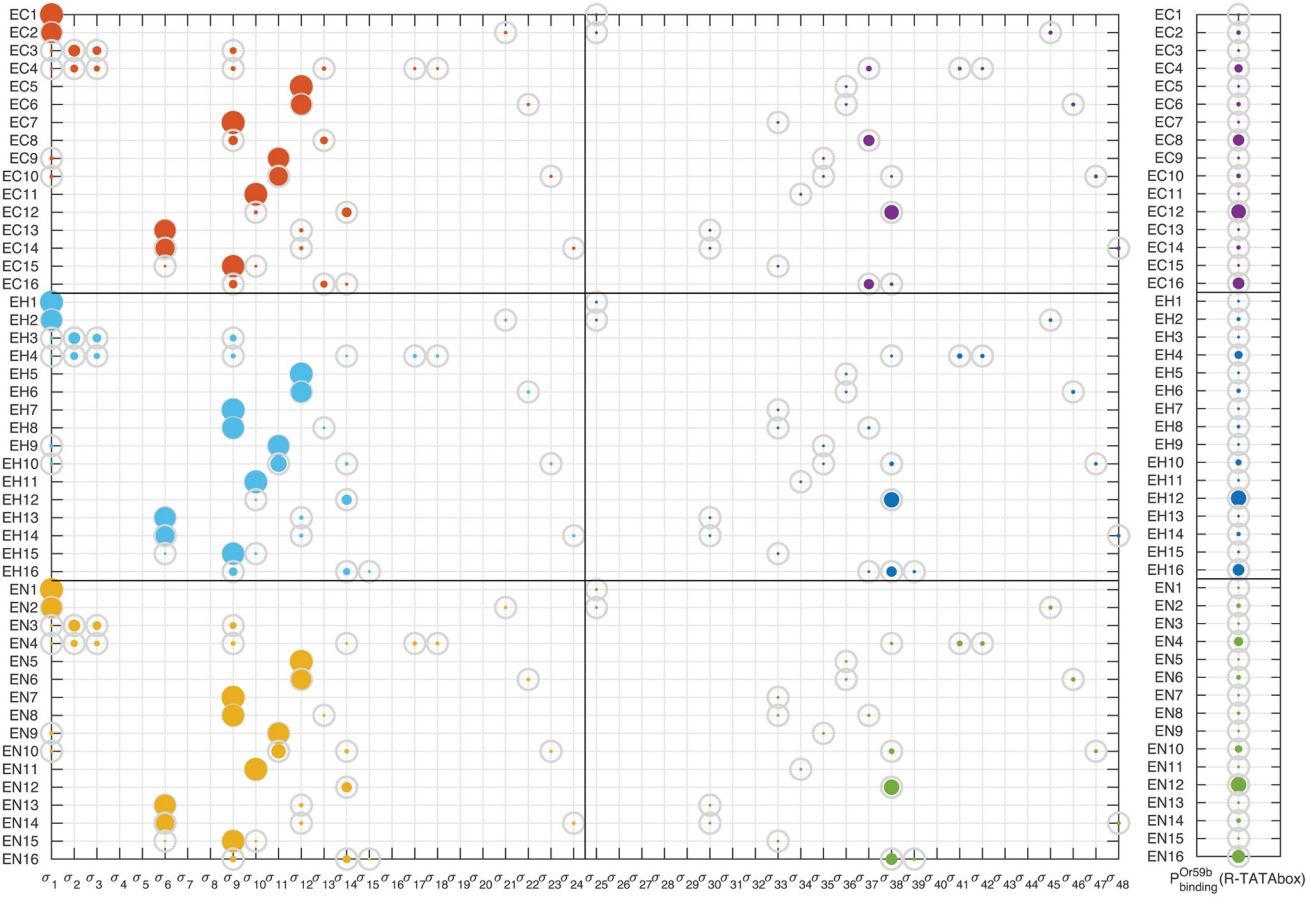
Statistical weights of the *σ*_*k*_ configurations for one sample. Normalized statistical weights *P*(*σ*_*k*_) = *p*_*k*_/*Z*_*tot*_ of the 48 possible configurations (horizontal axis) for the 16 × 3 mutations of the truth table (vertical axis) in one choice of parameter values that fits all the interval constraints of the truth table (Table 1). For each row the weights *P*(*σ*_*k*_) must sum to 1. The size of a dot is proportional to the weight. The gray circles correspond to the unity. The left (resp. right) half of the table corresponds to states for which RNAp is not (resp. is) bound to the TATAbox, see Figs. A-B of S1 Text. $P_{\text{binding}}^{\text{Or}59b}\left(R-\text{TATAbox}\right)$ (i.e., sum of the right half of the table) is represented in the rightmost panel.

Details of the sampling in parameter space are provided in the Methods and S1 Text. For the feasible parameter sets (i.e., values of *q*_*j*_, *w*_*jn*_ and *h*_*m*_ such that $P_{\text{binding}}^{\text{Or}59b}(R-\text{TATAbox})$ fulfills all constraints of Table 1), the distribution of the resulting $P_{\text{binding}}^{\text{Or}59b}(R-\text{TATAbox})$ in each of the 16 rows of the truth table for the three cases C, H and N is shown in Fig 2(A)–2(C). For one of the samples, the contribution of the 48 configurations *σ*_*k*_ to $P_{\text{binding}}^{\text{Or}59b}\left(R-\text{TATAbox}\right)$ is shown in Fig 3. For the ensemble of samples fitting the entire truth table, the empirical distributions of the probabilities *P*(*σ*_*k*_) in the various rows of the truth table are shown in Figs. E-L of S1 Text.

**Fig 4:**
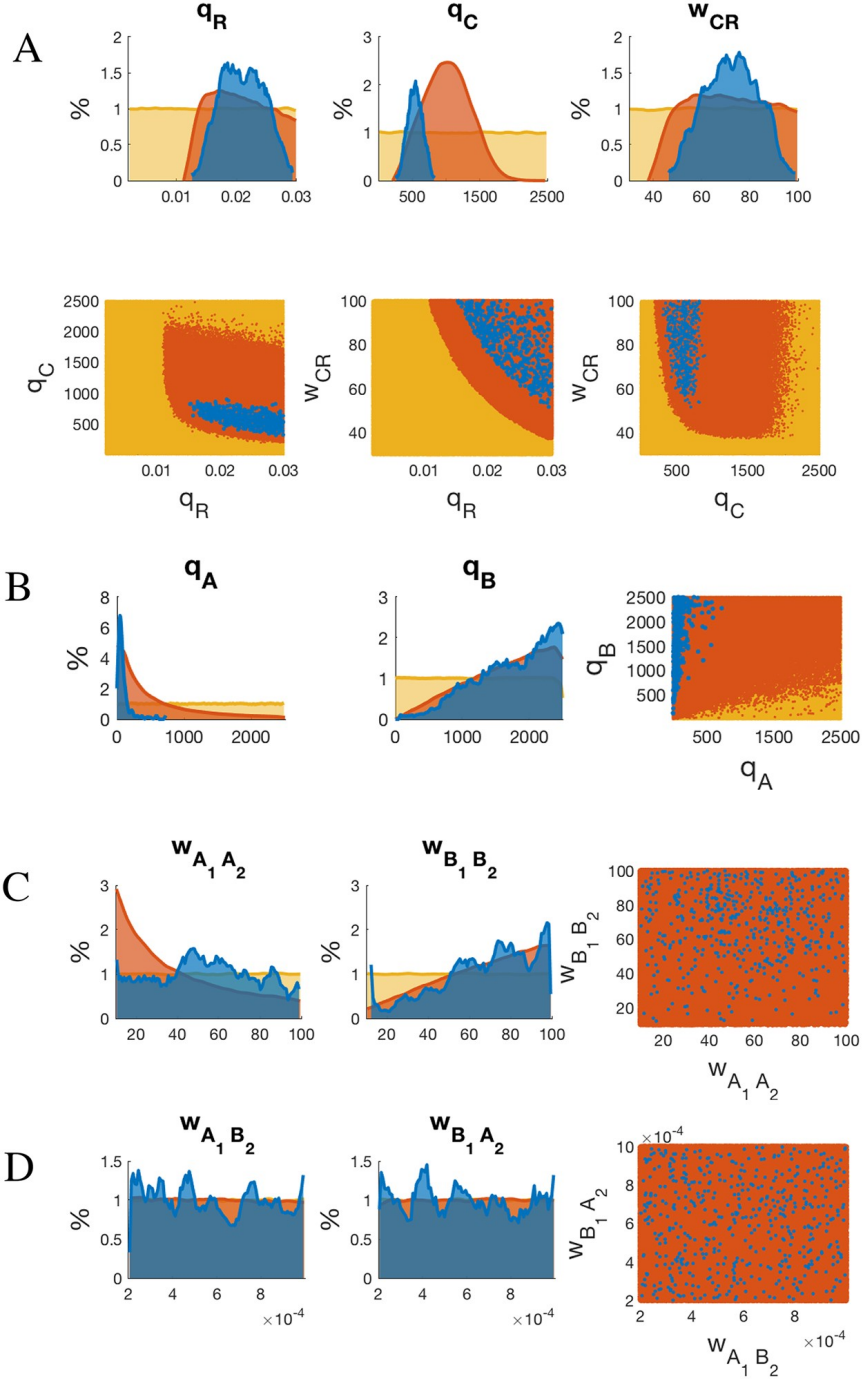
Analysis of the parameter fitting. (A): Top row: sample histograms of the parameters *q*_*R*_, *q*_*C*_ and *w*_*C*_. Yellow represents the entire sample population (uniform distribution), orange the samples for which the distances Φ^*C*^ and Φ^*H*^ are below the threshold *τ* = 0.05, and blue the samples fitting the entire truth table. Lower row: correlation between pairs of parameters in the 3 cases (same color code). For all 3 parameters, the orange histograms are no longer uniform, but restricted to smaller ranges. Such ranges concentrate even further for the feasible samples (blue), in particular the interval for *q*_*C*_ becomes quite tight. The correlation plots indicate that the boundaries between parameters subsets are well-defined and sharp. In particular, both *q*_*R*_ and *w*_*CR*_ have to be big enough in order to fulfill the entire truth table (i.e., blue points are in the top right corner). Notice how instead the binding affinity *q*_*C*_ cannot be big. (B): Sample histograms of the parameters *q*_*A*_ and *q*_*B*_, and their correlation. Notice the sharp difference in the two histograms: *q*_*A*_ ≪ *q*_*B*_ for feasible samples. (C): Sample histograms of the interaction coefficients $w_{A_{1}A_{2}}$ and $w_{B_{1}B_{2}}$. The two orange histograms have a neat difference, which is however only partially reflected in the feasible samples (blue). (D): Sample histograms of the interaction coefficients $w_{A_{1}B_{2}}$ and $w_{B_{1}A_{2}}$. No clear trend appears.

If we look at the distribution of the parameter values, we obtain a few significant relationships. First and foremost, feasible samples appear only when *q*_*C*_ assumes values in a precisely defined interval, see Fig 4(A). This is coherent with other experiments reported in [26], showing that overexpression of Fer1 in normal chromatin state does not lead to higher Or59b expression (higher concentration of a TF is associated to higher *q*_*j*_, according to Eq (1)). Also *q*_*R*_ and *w*_*CR*_ are restricted, although less drastically. It is also worth observing the stark contrast in the binding affinities between feasible *q*_*A*_ and *q*_*B*_, with the latter always much bigger than the former. The weak binding affinity *q*_*A*_ is compensated by a strong epigenetic coefficient *h*_*A*_ and viceversa for the pair *q*_*B*_ and *h*_*B*_. Furthermore, *h*_*A*_ increases when chromatin opens while *h*_*B*_ decreases, meaning that although unstable in its interaction with the DNA, Acj6 bound with both its domains to the DNA is likely to play a stronger role as enhancer of Fer1 binding than Pdm3 when chromatin opens.

## Discussion

The combinatorial complexity of the regulation in eukaryotic organisms like *Drosophila* is so high that understanding in detail what drives gene expression remains an elusive task, and a case-by-case analysis is often the only possible solution. In our system, to complicate further the picture is the fact that the specificity of the regulatory action may be lost when high-throughput techniques such as genome-wide transcriptomics, TF-DNA binding and chromatin accessibility are used, as they would not distinguish between class-specific and ectopic contributions. For the Or59b gene, in this paper we have developed a realistic biochemical first principles model based on statistical thermodynamics principles, suitable for unraveling the regulatory mechanisms behind transcription [9, 12, 13, 15, 16, 27]. Although this class of models has been used in broadly different contexts in recent times, [8, 10, 11, 18, 28], it was originally developed for studying prokaryotic gene regulation [15, 16]. A crucial prerequisite for applying it to our eukaryotic gene regulation is the abundance and variety of perturbative experiments performed in previous studies for this system [24, 26]. Since time-series and concentration profiles are not available, equilibrium probabilities must be used to predict expression. Given that we need to distinguish class-specific expression from ectopic expression, only a manual assessment of the transcription level induced by the Or59b cluster is possible, obtained by counting the number of OSN in the correct glomerulus, estimated through a GFP reporter, see Table A of S1 Text. The resulting expression level is described by an interval, representing the min and max of such counts in multiple flies. Currently, this is the only measurement available for our system. A common source of information that is used in thermodynamical models to reduce the number of free parameters is the computation of binding affinities for TF-DNA motifs pairs based on sequence [8, 18]. However, since our binding sites are short and non-consensus, any such computation would be subject to a large uncertainty, uncertainty which would propagate to the rest of the model. We prefer to treat the binding affinities *q*_*j*_ as free parameters in our model. Nontheless, it is worth remarking that our measurements are produced in a cohort of independent, “truly perturbative” experiments, which provide a significative amount of insight into the functioning of the Or59b cluster regulation. The model has a total of 18 free parameters (more properly, 28 parameters, if we count the five epigenetic parameters *h*_*m*_ three times), while the number of experiments in Table 1 is 19 (actually we could say ∼ 40 if we consider that all gray cells in Table 1 are known to lead to total loss), meaning that the ratio between experiments and parameters in unusually high for a model of this type.

Nucleosome-mediated accessibility of the TFs to the DNA is a well-documented phenomenon in *Drosophila* [29, 30], and so is the cross-talk between the organization of DNA in chromatin and the spatial arrangement of the binding sites [31]. Histones methylation can either increase or decrease gene expression, depending on which precise amino acids in the histones are methylated, and on the amount of methyl groups that are bound. Methylation events that weaken chemical attractions between histone tails and DNA enable uncoiling from nucleosomes, favoring access to DNA for regulators and RNAp. In our case, changes in H3K9 trimethylation indicate that the state of chromatin affects significantly the regulation of Or59b cluster function. In particular, we have shown in [26] that the use of a mutant su(var)3-9, the enzyme that trimethylates H3K9, results in different patterns of expression with respect to the normal chromatin state. Two variants of this mutation can be used: a heterozygous mutant su(var)3-9 (columns H in Table 1), used in [26], and a homozygous mutant su(var)3-9 (column N in Table 1), used in this study. Our hypothesis that the second mutant leads to a “more open” chromatin state than the first one is validated by the data we obtained. In particular, the trend observed in the behavior of the three main single site mutants of the Or59b cluster (E8, E12, and E14) in passing from the epigenetic condition C to H is confirmed by our new experiments in column N of Table 1. Remarkably, if we allow retuning of the epigenetic parameters but keep binding affinities and regulatory interactions fixed, also a model fitted on the first two epigenetic conditions is predicting well the behavior of the system in the third epigenetic condition (columns N), thereby suggesting that a model-based analysis may provide reasonable insight into the combinatorial regulation induced by the Or59b cluster, and on how this changes with the epigenetic background.

**Fig 5:**
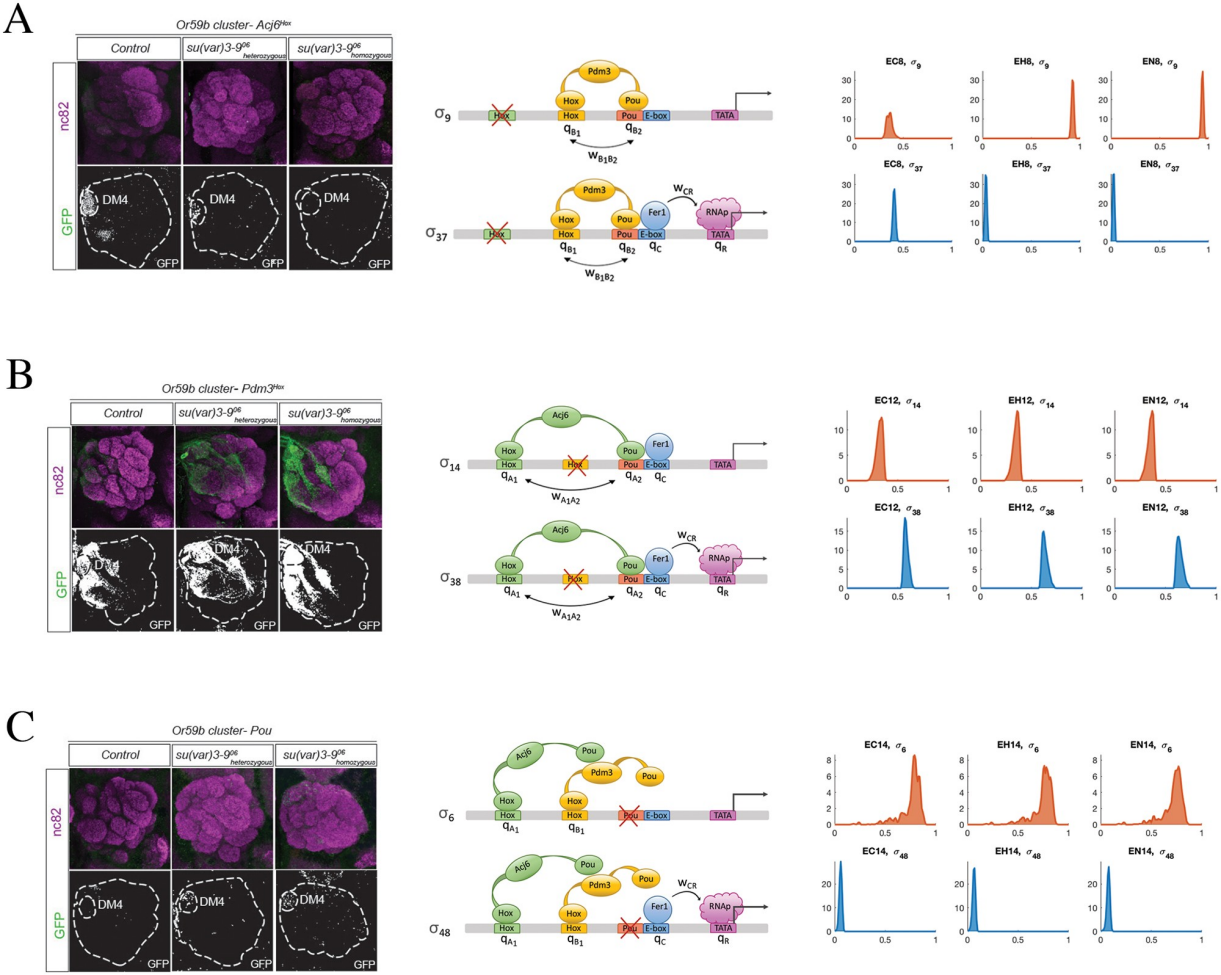
Single binding site mutants and their expression. (A): Mutation of Acj6Hox (i.e., E8 in Table 1). Left panel: GFP expression decreases passing from normal chromatin state (EC8) to heterozygous su(var)3-9 mutant (EH8) and to homozygous su(var)3-9 mutant (EN8). Middle panel: in our model, the two configuration states that contribute the most in this case are *σ*_9_ and *σ*_37_. Right panel: the corresponding distributions of *P*(*σ*_9_) (no Or59b expression) and *P*(*σ*_37_) (expression, but very weak) are reported. See Fig. H of S1 Text for all 48 probability histograms. (B): Mutation of Pdm3Hox (i.e., E12 in Table 1). Left panel: GFP expression is very high on all 3 epigenetic conditions (ectopic expression is not considered in the paper). Middle panel: the on-state is *σ*_38_ and the main off-state is *σ*_14_. Right panel: the on-state has a high probability: *P*(*σ*_38_). See Fig. J of S1 Text for complete histograms of all *σ*_*k*_. (C): Mutation of Pou motif (i.e., E14 in Table 1). Left panel: GFP expression increases slightly passing from normal chromatin state (EC14) to heterozygous su(var)3-9 mutant (EH14) and to homozygous su(var)3-9 mutant (EN14). Middle panel: the main on-state is *σ*_48_ and the main off-state is *σ*_6_. Right panel: the probability of the on-state, i.e. *P*(*σ*_38_) slightly increases passing from EC14 to EH14 and to EN14. See Fig. K of S1 Text for complete histograms of all *σ*_*k*_.

It is plausible to assume that mutation in one Homeobox site enables a stronger binding of the other TF to the DNA because of the reduced spatial competition. In normal chromatin state, such mechanism should favor transcription through a chain of synergistic actions: double binding of Acj6 or Pdm3 enabling recruitment of Fer1, in turn inducing RNAp binding. This is only partially true in our experimental data: while in E12 expression is strong, it is low in E8, sign that the two TFs Acj6 and Pdm3 act with different modalities when they have limited interference from other TFs. It is interesting to look at what happens in altered chromatin background in these two cases. While in E8 expression decreases when chromatin becomes open, in E12 we observe a similar strong expression across all epigenetic conditions. In our model, the behavior of E8 is attributed to only a couple of configuration states, *σ*_9_ and *σ*_37_, both corresponding to Pdm3 being bound to the DNA with both of its domains, as expected, see Fig 5(A). The state *σ*_37_, which presents in addition Fer1 bound to Ebox, becomes less probable as the chromatin opens, in favor of *σ*_9_ which lacks Fer1 binding (and does not lead to transcription). The model therefore suggests that double binding of Pdm3 becomes stronger as the chromatin becomes more open, and hampers Fer1 binding, likely through spatial competition. A similar effect is not shown by Acj6. In E12, the two dominant configurations (*σ*_14_ and *σ*_38_) are still with Acj6 doubly bound to both Homeobox and Pou domains, see Fig 5(B). However, the balance here remains significantly towards *σ*_38_ even as the chromatin opens, i.e., double binding of Acj6 still helps Fer1 binding to Ebox and drives transcription. The interpretation that we can give of this difference is that doubly bound Pdm3 is an obstacle to Fer1 binding in open chromatin. On the contrary, double binding of Acj6 seems to favor Fer1 binding, regardless of chromatin state, and, in fact, Fer1 is bound even in the (low-probability) no-expression state *σ*_14_. This happen in spite of a smaller binding energy for doubly bound Acj6 (parameter *q*_*A*_) than for doubly bound Pdm3 (parameter *q*_*B*_), see Fig 4(A) (and Methods for a description of these parameters—low *q*_*A*_ value means lower “effective” binding energy of Acj6 bound to both Homeobox and Pou domains). While the cooperative interactions $w_{A_{1}A_{2}}$ and $w_{B_{1}B_{2}}$ representing double binding have distributions of values with no clear trend, see Fig 4(C), the model clearly attributes the different behavior of E8 and E12 to the epigenetic factors: *h*_*A*_ ≫ *h*_*B*_, see Table 4. Recall that the role of *h*_*A*_ and *h*_*B*_ is to epigenetically remodulate the cooperativity coefficients $w_{A_{1}A_{2}}$ and $w_{B_{1}B_{2}}$ in configurations in which Fer1 is bound to Ebox. The most plausible explanation for the diverging difference between E8 and E12 is a diverging strength of the cooperativity actions.

The fundamental role of Pou as driver for Fer1 binding is confirmed in E14. With closed chromatin, expression is nearly lost (no TF has a stable—double motif—binding, hence rarely Fer1 can access the Ebox site). However, when chromatin becomes less densely packed around the DNA, Fer1 binding increases slightly, see Fig 5(C). Our model predicts this expression to be induced mainly by the configurations *σ*_48_, i.e., binding of Acj6 and Pdm3 to the respective Homeobox domains favoring Fer1 binding.

Also the description suggested by our model for the intact cluster case E16 is coherent with the picture delineated above. In fact, in our model, expression in normal chromatin in E16 is mostly due to *σ*_37_, i.e., to Pdm3 doubly bound to the DNA and helping Fer1 binding. However, with su(var)3-9 mutants, the most important state for transcription becomes instead *σ*_38_, i.e., Acj6 doubly bound to DNA, see Fig 1(C). In other words, when the chromatin becomes less densely packed a doubly bound Pdm3 changes from being an helper of transcription to being an obstacle, while the importance of doubly bound Acj6 as an expression driver is increased. This picture is in agreement with our deductions for the cases E8 and E12 above. For E16, notice how in the H column the experiments produced two different phenotypes: loss of expression and “normal expression”, see Table A of S1 Text. The prediction of the model is consistently for the latter, see Fig 2(B).

When we combine these results with E4 (interpreted as mutation on both Homeobox sites), the strong asymmetry between *q*_*A*_ and *q*_*B*_ shown in Fig 4(B) reflects in the different regulatory importance of Acj6 and Pdm3 when only binding to Pou can happen. In Fig. F of S1 Text, in fact, the configuration *σ*_41_ (Pdm3 bound to Pou) is more important than *σ*_42_ (Acj6 bound to Pou). How much this indirect experiment can be trusted as an accurate proxy for a double Homebox mutant is however unclear. We cannot exclude that the binding to the Pou domain may play a more significant role than the one attributed here in describing the altered phenotypes in response to a changing chromatin background.

It is worth stressing that fitting the values of the binding affinities *q*_*j*_ and interaction factors *w*_*jn*_ for the columns C and H is already impossible without introducing epigenetic parameters with values that change passing from C to H. Indirectly, this suggests that the TF-TF regulatory mechanisms included in the paper are not redundant, and that our model is not an overfitting of a simpler behavior. Combining this with the fact that *h*_*m*_ must change in passing from C to H, we expect that a correct prediction of the new data for the homozygous su(var)3-9 mutant (column N) cannot happen unless we retune the epigenetic parameters to the new background. Because of this retuning, we cannot claim to have a complete validation of the model prediction, but only a partial validation up to epigenetic adjustment.

Finally, it is also worth stressing that even disregarding completely the model, the new experiments in column N confirm basically all trends observed between columns C and H. This fact is itself of independent value, because it provides evidence in support of a basic assumption made in the paper, namely that the various epigenetic backgrounds lead to a progressive “opening” of the chromatin. The model we use is essentially describing how the balance between the different regulatory mechanisms shifts in response to an alteration of the chromatin packaging.

## Materials and methods

### Methods

This paper proposes a model for the regulation of the Or59b cluster based on statistical thermodynamics [9, 12–18, 27, 28]. For our system, the overall regulation can be decomposed into three distinct classes of interactions: (a) the interactions between TFs and the genomic sequence (TF-DNA), (b) the interactions among the TFs (TF-TF) and with the RNA polymerase (TF-RNAp), and (c) the interactions with the epigenome. These three classes are considered for building the model, based on the known TFs regulatory functions. Following [32] and [16], we assume that the level of gene expression is proportional to the rate of transcription initiation, that in turn depends on the equilibrium probability of RNAp binding the promoter of interest. The model assumes that the molecules involved bind to the DNA at thermodynamic equilibrium, and computes the probability of RNAp occupancy using TF binding affinities and interaction strengths in equilibrium states.

#### Binding reactions

The TF-DNA interactions addressed by the model are the binding of three transcription factors Acj6, Pdm3 and Fer1 to four binding sites Acj6Hox, Pdm3Hox, Pou and Ebox. Let us denote Acj6, Pdm3, Fer1 and RNAp as A, B, C and R respectively. If *A*_1_ (resp. *B*_1_) represents the domain of *A* (resp. *B*) that binds to the Homeobox site, and *A*_2_ (resp. *B*_2_) refers to the domain of *A* (resp. *B*) that binds to the Pou site, the possible TF-DNA binding reactions that can take place are:

$$\begin{array}{c} {\text{A}}_{1}+{\text{Hox}}_{{\text{A}}_{1}}\overset{k_{1}}{\underset{k_{-1}}{⇌}}{\text{A}}_{1}-{\text{Hox}}_{{\text{A}}_{1}}\\ {\text{A}}_{2}+\text{Pou}\overset{k_{2}}{\underset{k_{-2}}{⇌}}{\text{A}}_{2}-\text{Pou}\\ {\text{B}}_{1}+{\text{Hox}}_{{\text{B}}_{1}}\overset{k_{3}}{\underset{k_{-3}}{⇌}}{\text{B}}_{1}-{\text{Hox}}_{{\text{B}}_{1}}\\ {\text{B}}_{2}+\text{Pou}\overset{k_{4}}{\underset{k_{-4}}{⇌}}{\text{B}}_{2}-\text{Pou}\\ \text{C}+\text{Ebox}\overset{k_{5}}{\underset{k_{-5}}{⇌}}\text{C}-\text{Ebox}\\ \text{R}+\text{TATAbox}\overset{k_{6}}{\underset{k_{-6}}{⇌}}\text{R}-\text{TATAbox} \end{array}$$

where Hox_A_1__, Hox_B_1__, Pou, Ebox and TATAbox are the specific binding sites in the DNA for *A*, *B*, *C* and *R*, and the right hand side contains the TF-DNA complexes.

At equilibrium, the concentration of the species remains constant. We denote the equilibrium dissociation constants of the species from the DNA: $K_{A_{1}}=\frac{k_{-1}}{k_{1}}$, $K_{A_{2}}=\frac{k_{-2}}{k_{2}}$, $K_{B_{1}}=\frac{k_{-3}}{k_{3}}$, $K_{B_{2}}=\frac{k_{-4}}{k_{4}}$, $K_{C}=\frac{k_{-5}}{k_{5}}$ and $K_{R}=\frac{k_{-6}}{k_{6}}$.

The probability that a binding site $i=\{{\text{Hox}}_{A_{1}},{\text{Hox}}_{B_{1}},\text{Pou},\text{Ebox},\text{TATAbox}\}$ is occupied by a ligand *j* = {*A*_1_, *A*_2_, *B*_1_, *B*_2_, *C*, *R*} can be obtained through the Hill equations shown below. These equations use the concentration of the substrates [*A*_1_], [*A*_2_], [*B*_1_], [*B*_2_], [*C*], [*R*], and the values of the dissociation constants $K_{A_{1}}$, $K_{A_{2}}$, $K_{B_{1}}$, $K_{B_{2}}$, *K*_*C*_ and *K*_*R*_. The latter are naturally interpreted as the concentration of the ligand needed in order to have a 1/2 probability of the receptor being occupied. We denote the ratio between the probability of each site being bound vs unbound by the corresponding molecule as *q*_*j*_, see Table 2. Then, for *A*_1_, *A*_2_, *B*_1_, *B*_2_, *C* and *R* these ratios are $q_{A_{1}}$, $q_{A_{2}}$, $q_{B_{1}}$, $q_{B_{2}}$, *q*_*C*_ and *q*_*R*_, and we can write

$$\begin{array}{ll} P_{\text{binding}}\left({\text{A}}_{1}-{\text{Hox}}_{{\text{A}}_{1}}\right) & =\frac{\left[A_{1}\right]}{K_{A_{1}}+\left[A_{1}\right]}=\frac{q_{A_{1}}}{1+q_{A_{1}}}\\ P_{\text{binding}}\left({\text{A}}_{2}-\text{Pou}\right) & =\frac{\left[A_{2}\right]}{K_{A_{2}}+\left[A_{2}\right]}=\frac{q_{A_{2}}}{1+q_{A_{2}}}\\ P_{\text{binding}}\left({\text{B}}_{1}-{\text{Hox}}_{{\text{B}}_{1}}\right) & =\frac{\left[B_{1}\right]}{K_{B_{1}}+\left[B_{1}\right]}=\frac{q_{B_{1}}}{1+q_{B_{1}}}\\ P_{\text{binding}}\left({\text{B}}_{2}-\text{Pou}\right) & =\frac{\left[B_{2}\right]}{K_{B_{2}}+\left[B_{2}\right]}=\frac{q_{B_{2}}}{1+q_{B_{2}}}\\ P_{\text{binding}}(\text{C}-\text{Ebox}) & =\frac{\left[C\right]}{K_{C}+[C]}=\frac{q_{C}}{1+q_{C}}\\ P_{\text{binding}}(\text{R}-\text{TATAbox}) & =\frac{\left[R\right]}{K_{R}+[R]}=\frac{q_{R}}{1+q_{R}}. \end{array}$$

From these expressions, we can also obtain the *q*_*j*_ terms as ratios between the concentrations and dissociation constants as

$$\begin{array}{c} \begin{array}{cc} q_{A1} & =\frac{\left[A_{1}\right]}{K_{A1}}\\ q_{A2} & =\frac{\left[A_{2}\right]}{K_{A2}}\\ q_{B1} & =\frac{\left[B_{1}\right]}{K_{B1}}\\ q_{B2} & =\frac{\left[B_{2}\right]}{K_{B2}}\\ q_{C} & =\frac{\left[C\right]}{K_{C}}\\ q_{R} & =\frac{\left[R\right]}{K_{R}}. \end{array} \end{array}$$ (1)

More details on these derivations are provided in the S1 Text.

#### Description of the interaction factors

If a bound ligand *j* interacts with another bound ligand *n* with *n* ≠ *j*, the interaction term *w*_*jn*_ is modeled as

$$\begin{array}{c} w_{jn}\{\begin{array}{cc} >1 & \text{if}\;\text{interaction}\;\text{is}\;\text{cooperative}\\ =1 & \text{if}\;\text{no}\;\text{interaction}\;\text{occurs}\\ <1 & \text{if}\;\text{interaction}\;\text{is}\;\text{competitive}. \end{array} \end{array}$$ (2)

Interactions among molecules can be classified into TF-RNAp interactions and TF-TF interactions. In the first group only the positive direct interaction of Fer1 with RNAp, denoted *w*_*CR*_, is considered, as Fer1 has been demonstrated to be an activator very likely involved in the recruitment of RNAp [26]. In fact, the phenotype for Ebox mutation is total loss (row E15 in Table 1).

To the second group belong interactions of both cooperative and competitive nature. These are the cooperative interactions of the two-domain Homeobox-Pou proteins, denoted $w_{A_{1}A_{2}}$ and $w_{B_{1}B_{2}}$, and the competitive effect of a TF attached to Homeobox on the other TF attached to Pou, denoted $w_{A_{1}B_{2}}$ (when A is attached to the Homeobox site and competes with B bound to the Pou site) and $w_{B_{1}A_{2}}$ (when B is attached to the Homeobox site and competes with A bound to the Pou site).

Therefore, the parameters *w*_*CR*_, $w_{A_{1}A_{2}}$ and $w_{B_{1}B_{2}}$ take values greater than the unit, since they contribute positively (directly or indirectly) to the initiation of transcription. This is translated into a higher value of the statistical weight for the corresponding molecular configurations, thus affecting the overall RNAp binding probability. On the contrary, the parameters $w_{A_{1}B_{2}}$ and $w_{B_{1}A_{2}}$ take values between 0 and 1, as they make less probable the configuration in which they appear.

#### Statistical thermodynamic model of gene expression

The DNA template and all the molecules that take part in the regulation of transcription lead to 48 possible molecular states, i.e., distinct configurations in which the system can be arranged, denoted *σ*_*k*_ with *k* = 1, …, 48. A state is a configuration of the TFs and of the corresponding specific binding sites. In this system we have four TFs (*A*, *B*, *C*, *R*), two of them with two distinct domains (*A*_1_, *A*_2_, *B*_1_, *B*_2_), and five binding sites (Hox_A_1__, Hox_B_1__, Pou, Ebox, TATAbox). The 48 states *σ*_*k*_, shown in Figures A-B of S1 Text, represent all admissible combinations of TF-DNA binding and TF-TF or TF-RNAp interactions. Each state *σ*_*k*_ is given a statistical weight, or partial partition function, *p*_*k*_ that is calculated from the interaction factors among bound molecules *w*_*jn*_ and from the *q*_*j*_ terms given above. Additional factors are introduced in *p*_*k*_ accounting for the epigenetic interactions (*h*_*m*_) and will be explained later in detail.

In summary, the partial partition function *p*_*k*_ is the product of contributions of all occupied sites and all the interactions implied by the configuration *σ*_*k*_:

$$\begin{array}{c} p_{k}=p(\sigma_{k})=\prod_{j}q_{j}\prod_{n}w_{jn}\prod_{m}h_{m} \end{array}$$ (3)

with *k* = 1, …, 48. See S1 Text for a derivation of these terms from first principles, and Eq. (I)-(J) of S1 Text for the explicit expression of the *p*_*k*_ terms.

The total partition function is equal to the sum of the statistical weights of all the possible molecular configurations in which the system can be, that is $Z_{tot}=\sum_{k=1}^{48}p_{k}$. The equilibrium probability of a certain configuration is obtained as the ratio between the statistical weight of the configuration and the total partition function, which acts as a normalization constant: $P(\sigma_{k})=\frac{p_{k}}{Z_{tot}}$.

The observable of the system is the probability of Or59b cluster driven expression, represented in the model as the probability of RNA polymerase binding to the TATAbox, denoted $P_{\text{binding}}^{\text{Or}59b}(R-\text{TATAbox})$. Unlike *P*_binding_(*R* − TATAbox), this probability is now formulated in terms of the overall regulatory structure considered: it is the sum of all configurations *σ*_*k*_ in which the RNAp is bound to the promoter, divided by the total partition function *Z*_*tot*_, i.e.:

$$\begin{array}{c} P_{\text{binding}}^{\text{Or}59b}(R-\text{TATAbox})=\frac{\sum_{k=25}^{48}p_{k}}{\sum_{k=1}^{48}p_{k}}. \end{array}$$ (4)

From Eq (3), the statistical weight of each state *σ*_*k*_ is the product of the *q*_*j*_ terms of the ligand molecules that are present in that particular state, of the interactions among them *w*_*jn*_ and of the epigenetic factor *h*_*m*_, hence also $P_{\text{binding}}^{\text{Or}59b}(R-\text{TATAbox})$ is a function of *q*_*j*_, *w*_*jn*_ and *h*_*m*_.

#### Description of the epigenetic factors

The third type of interactions included in the model, *h*_*m*_, are of epigenetic nature. They are needed to describe the different behavior of the chromatin in the su(var)3-9 mutations. In our model, the binding affinities *q*_*j*_ and the interaction factors *w*_*jn*_ describe independent processes seen “in isolation”. We assume that the epigenetic parameters do not alter these quantities, but can modify the probabilities of the states *σ*_*k*_ in which these terms appear, according to Eq (3).

Under our assumption, when the chromatin is closed, Fer1 can normally bind Ebox only if there is a TF attached to the Pou site. However, with an su(var)3-9 mutant, a TF bound to Pou is no longer strictly necessary for Fer1 binding. To describe the states in which Fer1 is bound to Ebox with no protein bound to Pou, the epigenetic interaction terms *h*_1_ and *h*_2_ are introduced. The parameter *h*_1_ appears in the configurations in which there is a Fer1 bound to the Ebox site with no Acj6 nor Pdm3 bound to the entire cluster (i.e. states *σ*_21_ and *σ*_45_ in Figure B of S1 Text, see also Eq. (I)-(J) of S1 Text). The parameter *h*_2_ appears when there is a Fer1 bound to Ebox with no Acj6 nor Pdm3 bound to the Pou site (i.e. states *σ*_22_, *σ*_23_, *σ*_24_ and *σ*_46_, *σ*_47_, *σ*_48_). The reason for treating these two cases differently is because one of these TFs attached to a Homeobox motif may be an obstacle to Fer1 binding. The states in which these terms appear are negligible in normal chromatin and intact cluster, but they become relevant when chromatin is trimethylated by mutant su(var)3-9 and cluster site mutations are considered (e.g. E6, E10, E14 in Table 1).

The modification of the chromatin that follows a su(var)3-9 mutation also impacts the cooperativity due to the interactions $w_{A_{1}A_{2}}$ and $w_{B_{1}B_{2}}$. Two epigenetic factors *h*_*A*_ and *h*_*B*_ are introduced to modulate the corresponding configurations, in particular those in which Fer1 is bound to Ebox (i.e., states *σ*_14_, *σ*_15_, *σ*_38_, *σ*_39_ for *h*_*A*_ and *σ*_13_, *σ*_16_, *σ*_37_, *σ*_40_ for *h*_*B*_). A final epigenetic interaction, denoted *h*_3_, can be introduced, to take into account the reduced concentration of methyltransferase in su(var)3-9 mutants. This in turn reduces the amount of Acj6 and Pdm3 captured in complexes with methyltransferase and can alter the frequency of the binding of these TFs to Pou, thereby moving the balance point in the spatial competition between Acj6/Pdm3 binding and Fer1 binding. The configuration potentially affected by this epigenetic term are *σ*_13÷20_ and *σ*_37÷44_, see Eq. (I)-(J) of S1 Text.

#### Effect of binding site mutations

We assume that mutations affecting a DNA binding site result in a residual binding affinity smaller by several order of magnitude, i.e., in the *q*_*A*_, *q*_*B*_ and *q*_*C*_ coefficients of our model the values of Table 2 are replaced by values in the range [10^−6^, 10^−5^]. The 16 mutations listed in Table 1 for each of the 3 epigenetic conditions C, H, and N give rise to a total of 48 possible experimental situations, denoted $\theta_{j}^{i}$, *i* = C, H, N, *j* = 1, …, 16. In the model, to each $\theta_{j}^{i}$ corresponds a different set of partial partition functions $p_{k,j}^{i}=p\left(\sigma_{k},\theta_{j}^{i}\right)$, *k* = 1, …, 48, *j* = 1, …, 16, *i* = C, H, N, obtained by replacing the *q*_*j*_ parameters with the residual binding affinities. Consequently, we have also 48 different values for the model output $P_{\text{binding}}^{\text{Or}59\text{b}}(\text{R}-\text{TATAbox},\theta_{j}^{i})$, *j* = 1, …, 16, *i* = C, H, N.

#### Constraints on the epigenetic parameters

As already mentioned, we assume that changes in the chromatin state do not alter the values of the binding affinities *q*_*j*_ and of the interactions factors *w*_*jn*_, meaning that the values of these parameters must remain constant in the three columns C, H, and N. Only the epigenetic parameters *h*_*m*_ are allowed to change when passing from one chromatin state to another. The general effect of changing the values of *h*_*m*_ is to alter the equilibrium probabilities of the states *σ*_*k*_ and hence the balance among the regulation mechanisms behind $P_{\text{binding}}^{\text{Or}59b}(R-\text{TATAbox})$. We make the assumption that the changes in *h*_*m*_ must be coherent across the three columns, i.e., if *h*_*m*_ increases (resp. decreases) passing from C to H it must increase (resp. decrease) also when passing from H to N.

#### Parameter fitting

Denote d(x,Y) the (Euclidean) set distance of a point *x* from a set $Y:d\left(x,Y\right)=\min_{y\in Y}{∥x-y∥}_{2}$. In particular, we are interested in sets that are intervals contained in [0, 1]: Y=[ℓ,u]⊆[0,1] (*ℓ* = lower bound, *u* = upper bound). The output of the model $P_{\text{binding}}^{\text{Or}59b}(R-\text{TATAbox},\theta_{j}^{i})$ is a function of the parameters *q*_*j*_, *w*_*jn*_ and *h*_*m*_. In order to fit numerical values for these parameters, the prediction error function that must be minimized is the following:

$$\Phi \left(q_{j},w_{jn},h_{m}\right)=\sum_{\begin{array}{l} i=\text{C},\;\text{H},\;\text{N}\\ j=1,\ldots ,16 \end{array}}d(P_{\text{binding}}^{\text{Or}59\text{b}}\left(\text{R}-\text{TATAbox},\theta_{j}^{i}\right),\left[l_{j}^{i},u_{j}^{i}\right]).$$ (5)

The bounds $\left[\ell_{j}^{i},\;u_{j}^{i}\right]$ are based on the available experimental data and are reported in Table 1. In particular, a set of parameters {*q*_*j*_, *w*_*jn*_, *h*_*m*_} is feasible if Φ(*q*_*j*_, *w*_*jn*_, *h*_*m*_) = 0, i.e., the predicted values of $P_{\text{binding}}^{\text{Or}59b}(R-\text{TATAbox})$ satisfy the bounds simultaneously for all experiments. The cost function in Eq (5) is highly nonlinear: it is a set distance involving a sum of products of the unknown parameters, see Eqs (3) and (4) (and Eq. (I)-(J) of S1 Text). We are not aware of any effective algorithm (for instance of gradient type) able to iteratively solve the minimization problem in Eq (5). We therefore resorted to a random sampling of the parameter space. The sample was uniform in the *q*_*j*_ and *w*_*jn*_ parameters, within the ranges given in Tables 2 and 3 (see S1 Text for a rationale behind these choices). We first looked at the normal chromatin state (column C), and selected values of {*q*_*j*_, *w*_*jn*_, *h*_*m*_} for which the distance in Eq (5) computed only on the column C (hereafter Φ^*C*^) is below a threshold *τ* = 0.05, see Fig. M(i) of S1 Text. For these parameter values we checked whether all ranges $\left[\ell_{j}^{i},\;u_{j}^{i}\right]$, *i* = *C*, *H*, *j* = 1, …, 16, could be fulfilled. Lack of success forced us to resort to epigenetic parameters *h*_*m*_ that vary with the chromatin state. In order to calibrate these epigenetic parameters, we selected values of *h*_*m*_ (hereafter $h_{m}^{C}$) leading to the correct phenotype in the C column alone (more properly, such that Φ^*C*^ < *τ*), and proceeded to vary again randomly the *h*_*m*_ in order to fit also the column H (obtaining a new set $h_{m}^{H}$). For each selection of $h_{m}^{C}$ and $h_{m}^{H}$ the actual value of the epigenetic parameter on a sample was drawn from a normal distribution centered at $h_{m}^{C}$ or $h_{m}^{H}$ and of standard deviation $h_{m}^{C}/10$ or $h_{m}^{H}/10$. Several batches of such quadruples $\left\{q_{j},\;w_{jn},\;h_{m}^{C},\;h_{m}^{H}\right\}$ were produced (each of ∼10^5^ samples), checking the values of RNAp binding probability for both columns C and H until we could identify values of $h_{m}^{\left\{C,H\right\}}$ for which both columns C and H have a sufficiently high fraction of samples below the distance threshold *τ* = 0.05, i.e., Φ^*C*^ < *τ* and Φ^*H*^ < *τ*. To achieve this, all 5 epigenetic parameters *h*_*m*_ had to be tuned. During this phase we also repeatedly reset all parameter values, to see if more parsimonious pairs $h_{m}^{C}$, $h_{m}^{H}$ could be found, without success. We stopped the procedure until a significant fraction of feasible parameter sets could be found (i.e., such that $P_{\text{binding}}^{\text{Or}59b}(R-\text{TATAbox},\theta_{j}^{i})\in \left[\ell_{j}^{i},\;u_{j}^{i}\right]$ for all *i* = C, H and *j* = 1, …, 16 in at least 0.5% of the samples with Φ^*C*^ < *τ* and Φ^*H*^ < *τ*).

The (partial) validation phase consisted in checking what happens in the remaining epigenetic state (homozygous su(var)3-9 mutant, column N). The parameters *q*_*j*_ and *w*_*jn*_ were kept constant, while values of the five epigenetic parameters $h_{\left\{1,\;2,\;3,\;A,\;B\right\}}^{N}$ were sought in order to fulfill the RNAp bounds on the N column (i.e., $P_{\text{binding}}^{\text{Or}59b}(R-\text{TATAbox},\theta_{j}^{N})\in \left[\ell_{j}^{N},\;u_{j}^{N}\right]$ for all *j* = 1, …, 16), with the following monotonicity constraints: $h_{\left\{2,3,A\right\}}^{C}<h_{\left\{2,3,A\right\}}^{H}<h_{\left\{2,3,A\right\}}^{N}$ and $h_{\left\{1,B\right\}}^{C}>h_{\left\{1,B\right\}}^{H}>h_{\left\{1,B\right\}}^{N}$, see Table 4 and Fig. D of S1 Text. As can be seen in Fig. M(ii) of S1 Text, after a proper tuning of $h_{m}^{N}$ more than 50% of the parameter sets leading to Φ^*C*^ < *τ* and Φ^*H*^ < *τ* also correspond to Φ^*N*^ < *τ*. Furthermore, the fraction of samples fitting all constraints exactly (i.e., $P_{\text{binding}}^{\text{Or}59b}(R-\text{TATAbox},\theta_{j}^{i})\in \left[\ell_{j}^{i},\;u_{j}^{i}\right]$ for all *i* = *C*, *H*, *N*, *j* = 1, …, 16, blue lines in Fig. M of S1 Text) is still fairly close to 0.5% of the total number of samples with Φ^*C*^ < *τ* and Φ^*H*^ < *τ*. The total number of samples drawn in the entire process was around 10^7^. The selected nominal values of *h*_*m*_ are reported in Table 4 and Fig. D of S1 Text, while the sample distributions of the feasible sets of the *q*_*j*_ and *w*_*jn*_ parameters are given in Fig 4 and Fig. C of S1 Text. In Fig. D of S1 Text, notice how the histograms of actual values of *h*_*m*_ for the feasible parameter sets are well distributed around the nominal values (shown in red), meaning that no local improvement in the fit is possible by (small) variations of the nominal *h*_*m*_.

### Materials

#### *Drosophila* stocks

The Pebbled-Gal4 (Peb-Gal4) was a kind gift from Liqun Luo (Stanford University, Stanford, CA, USA). The su(var)3-9^06^ mutant was a kind gift from Anita Öst (Linköping University, Linköping, Sweden). The following fly line were obtained from the Vienna *Drosophila* Center (VDRC; Vienna, Austria; http://stockcenter.vdrc.at): Fer1-IR, UAS-Dcr2. The following fly line was provided by the Bloomington *Drosophila* Stock Center (BDSC; Indiana University, Bloomington, IN, USA; http://flystocks.bio.indiana.edu): *w*^1118^. The following RNAi lines were obtained from the Transgenic RNAi Project (TRiP; Harvard Medical School, Boston, MA, USA; http://www.flyrnai.org): Fer1-IR (27737; 50672), Pdm3-IR (35726, 26749). The UAS-Acj6 fly was a kind gift from Dr. John Carlson (Carlson Lab / KBT 1142 Dept. of Molecular, Cellular, and Developmental Biology, Yale University, USA).

#### Cloning

All constructs were synthesized at Genescript and cloned into a transformation vector containing a synthetic TATA region fused to a single ORF that contained the mCD8 transmembrane domain, four tandem copies of GFP, and two *c-myc* epitope tags, as previously described [19]. The DNA constructs were injected into *w*^1118^ flies at BestGene, and 6 to 12 lines were analyzed per construct.

#### Immunofluorescence

Immunofluorescence was performed according to previously described methods [24]. The following primary antibodies were used: rabbit anti-GFP (1:2000, TP-401; Torrey Pines Biolabs) and mouse anti-nc82 (1:100; DSHB). Secondary antibodies were conjugated with Alexa Fluor 488 (1:500; Molecular Probes) and Rhodamine Red^™^-X, Succinimidyl Ester, 5-isomer (1:250; ThermoFisher Scientific). Confocal microscopy images were collected on an LSM 700 (Zeiss) and analyzed using an LSM Image Browser. The numbers of co-expressing Brp and GFP OSNs for different constructs were counted from the images. Adobe Photoshop CS4 (Adobe Systems) was used for image processing.

## Supporting information

S1 Text Supplementary methods, tables and figures. Description of the experiments.
(PDF)
