# Supplementary material for: Thermodynamic model of gene regulation for the Or59b olfactory receptor in Drosophila
Source: PLoS Comput Biol. 2019 Jan 17;15(1):e1006709. doi: 10.1371/journal.pcbi.1006709 (PMC6353224; doi:10.1371/journal.pcbi.1006709)
Supplement: S1 Text — Description of the experiments. (PDF) [file pcbi.1006709.s001.pdf]

Supplementary Information
Thermodynamic model of gene regulation for the Or59b olfactory receptor in Drosophila

A. González[1], S. Jafari[2], A. Zenere[1], M. Alenius[2] and C. Altafini[1*]
1. Division of Automatic Control, Dept. of Electrical Engineering,
2. Department of Clinical and Experimental Medicine,
Linköping University, Linköping, Sweden.

January 2, 2019

Contents

1 Supplementary Methods **1**
1.1 A recap of basic statistical thermodynamics models 1
1.2 Statistical thermodynamics applied to the Or59b cluster 3
1.3 Probabilities of σ_k and model parameters 15
1.4 Physical ranges for the model parameters 18
1.5 Random sampling of the parameter space 19

2 List of experiments **20**
2.1 Experiments concerning motifs mutation and chromatin trimethylation by heterozygous mutant su(var)3-9. 20
2.2 Experiments concerning motifs mutation and chromatin trimethylation by homozygous mutant su(var)3-9. 23

1 Supplementary Methods

1.1 A recap of basic statistical thermodynamics models

The material of this section is taken from e.g. [7]. See also [4, 3, 8, 9, 12].

Consider the idealized situation of an RNA polymerase (RNAp) binding to the DNA of a certain organism. The probability that a molecule is bound depends on the Gibbs free energy difference between the bound and unbound states $\Delta G = G(\text{bound}) - G(\text{unbound})$, which is negative when the "on" state has lower free energy

*Corresponding author: C. Altafini. Email: claudio.altafini@liu.se

than the “off ” state. For further explanation and details see *Appendix* and *Chapter 7* of [13].

If we are interested in transcription of a certain gene, the genome can be viewed as an ensemble of specific and non-specific binding sites. If N_{ns} is the number of non-specific sites, the RNAp can bind to any of these N_{ns} binding sites across the genome, or it may bind to one site of interest placed at the TATA box of the promoter of the target gene. The probability that this specific site is occupied by the RNAp is our main concern, as it gives an idea of the level of gene expression (the two quantities are proportional under thermodynamic equilibrium assumption). If we have a number R of RNA polymerase molecules, "states" in this case refer to the possible ways we can distribute our R polymerase molecules in the genome, and the probabilities of the states are described by a Boltzmann distribution. In particular, the unnormalized probability of a state is given by the number of ways the state can be realized, multiplied by the Boltzmann Factor that accounts for all the free energies. For simplicity, the state of the system is considered independent from the rest of the system, for instance the water that surrounds the molecules [13]. Thus, a first state in which all the R RNAp are bound to non-specific sites can be considered, and a second possible state in which one of the polymerases is bound to the promoter of interest while the remaining $R-1$ bind non-specifically. These two classes of states can be characterized as a distribution of polymerase molecules on the non-specific DNA. If X^S is the specific site of the promoter region where the RNAp can bind to, the binding reaction that can take place is

$$\mathrm{RNAp} + \mathrm{X^S} \underset{k_{-R}}{\overset{k_R}{\rightleftharpoons}} \mathrm{RNAp{-}X^S}.$$

Following the same notation as [7], the partial partition function of the first state, denoted $Z_{NS}\left(R, N_{ns}\right)$, is given by the product of the number of arrangements of the R molecules in N_{ns} non-specific sites and the Boltzmann weight. Note that the number of arrangements is the multiplicity of the different states that share the same Boltzmann factor. The notation ε_R^{NS} is used to characterize the binding energy for non-specific sites, although this is a simplistic assumption since there are different binding energies for the non-specific sites [9]. The partial partition function of the first state is then

$$Z_{NS}\left(R, N_{ns}\right) = \frac{N_{ns}!}{R!\left(N_{ns}-R\right)!} \cdot e^{-\beta R \varepsilon_R^{NS}}. \tag{A}$$

The partial partition function of the second group of states is denoted $Z_{NS}\left(R-1, N_{ns}\right) e^{-\beta\varepsilon_R^S}$ where ε_R^S is used to characterize the binding energy for the specific site in the promoter. The term $Z_{NS}\left(R-1, N_{ns}\right)$ is defined as the product of the number of arrangements of the $R-1$ molecules in N_{ns} non-specific sites times the Boltzmann weights and it is similar to (A), so that:

$$Z_{NS}\left(R-1, N_{ns}\right) e^{-\beta\varepsilon_R^S} = \frac{N_{ns}!}{(R-1)!\left(N_{ns}-(R-1)\right)!} \cdot e^{-\beta(R-1)\varepsilon_R^{NS}} \cdot e^{-\beta\varepsilon_R^S}. \tag{B}$$

The sum of the two partial partition functions is the total partition function:

$$Z\left(R, N_{ns}\right)=Z_{NS}\left(R, N_{ns}\right)+Z_{NS}\left(R-1, N_{ns}\right) e^{-\beta \varepsilon_R^S}. \tag{C}$$

The probability that RNAp binds to the promoter of interest is calculated as the ratio of the sum of configuration weights in which the RNAp is bound to this promoter, over the sum of all possible configuration weights, that is, the total partition function:

$$P_{\text{binding}}\left(R-X^S\right)=\frac{Z_{N_{ns}}\left(R-1, N_{ns}\right) e^{-\beta \varepsilon_R^S}}{Z_{N_{ns}}\left(R, N_{ns}\right)+Z_{N_{ns}}\left(R-1, N_{ns}\right) e^{-\beta \varepsilon_R^S}}. \tag{D}$$

Taking into account that the number of RNAp is much lower than the number of non-specific sites ($R \ll N_{ns}$), the combinatorial term can be simplified as $\frac{N_{ns}!}{(N_{ns}-R)!} \simeq (N_{ns})^R$. One can simplify equation (D) by multiplying the numerator and the denominator by $\frac{R!}{(N_{ns})^R e^{-\beta R \varepsilon_R^{NS}}}$. Equation (D) can then be rewritten as:

$$P_{\text{binding}}\left(P-X^S\right)=\frac{1}{1+\frac{N_{ns}}{R} \cdot e^{\beta\left(\varepsilon_R^S-\varepsilon_R^{NS}\right)}}. \tag{E}$$

Rather than the absolute value of a certain biding energy, what is really relevant for a particular molecule is the difference between the energy for a specific and a non-specific site. Hence, the true difference in the strength of a region is contained in the difference $\Delta\varepsilon=\varepsilon_R^S-\varepsilon_R^{NS}$ [4].

In the case of transcriptional regulation in the system of interest in this paper, we have more than two states, and so we have to consider all of them in the total partition function, with their respective energies and additional interactions.

1.2 Statistical thermodynamics applied to the Or59b cluster

The region of the DNA of *Drosophila melanogaster* called the Or59b cluster constitutes a system that can exist in several states. Each has a probability that is proportional to a Boltzmann Factor $e^{-\beta \cdot \varepsilon_i}$, where ε_i is the energy of that microstate and $\beta=\frac{1}{k_B T}$. The temperature T for biological systems is typically around $300K$, resulting in $k_B T \approx 0.6 kcal/mol \approx 2.5 kJ/mol$.

The states describe the binding of three transcription factors, Acj6, Pdm3 and Fer1, to four binding sites, Acj6Hox, Pdm3Hox, Pou and Ebox, plus the binding of RNAp to the TATA box of the Or59b gene. Acj6 and Pdm3 can bind to their respective Homeobox domain and to the same Pou domain. Similarly, Fer1 has a certain affinity for Ebox site. We denote Acj6, Pdm3, Fer1 and RNAp as A, B, C and R respectively. To simplify notation, these letters refer also to the number of molecules of these species, while square brackets $[\cdot]$ refer to their concentrations. In the case of Acj6 and Pdm3, both are regulatory proteins with two distinct binding domains. Thus, the notation A_1 and B_1 represents the domain that binds to the Homeobox site, while A_2 and B_2 refers to the domain that binds to the Pou site. Note that A_1 and A_2 are in number equal to the number of A transcription factors: $A_1=A_2=A$. Similarly, B_1 and B_2 are in number equal to the number of B

transcription factors: $B_1 = B_2 = B$. The total number of domains for each specie is $A' = A_1 + A_2 = 2A$ and $B' = B_1 + B_2 = 2B$. Thus, if A_1 (resp. B_1) represents the domain that binds to the Homeobox site, and A_2 (resp. B_2) refers to the domain that binds to the Pou site, then the possible binding reactions that can take place are

$$\mathrm{A_1} + \mathrm{Hox_{A_1}} \underset{k_{-1}}{\overset{k_1}{\rightleftharpoons}} \mathrm{A_1{-}Hox_{A_1}}$$

$$\mathrm{A_2} + \mathrm{Pou} \underset{k_{-2}}{\overset{k_2}{\rightleftharpoons}} \mathrm{A_2{-}Pou}$$

$$\mathrm{B_1} + \mathrm{Hox_{B_1}} \underset{k_{-3}}{\overset{k_3}{\rightleftharpoons}} \mathrm{B_1{-}Hox_{B_1}}$$

$$\mathrm{B_2} + \mathrm{Pou} \underset{k_{-4}}{\overset{k_4}{\rightleftharpoons}} \mathrm{B_2{-}Pou}$$

$$\mathrm{C} + \mathrm{Ebox} \underset{k_{-5}}{\overset{k_5}{\rightleftharpoons}} \mathrm{C{-}Ebox}$$

$$\mathrm{R} + \mathrm{TATAbox} \underset{k_{-6}}{\overset{k_6}{\rightleftharpoons}} \mathrm{R{-}TATAbox}$$

where $\mathrm{Hox_{A_1}}$, $\mathrm{Hox_{B_1}}$, Pou, Ebox and TATAbox are the specific binding sites in the DNA for A, B, C and R.

According to mass-action kinetics, the system of ODEs for the previous stoichiometric reactions is given as:

$$\begin{aligned}
[\dot{A_1}] &= k_{-1}\,[\mathrm{A_1} - \mathrm{Hox_{A_1}}] - k_1\,[\mathrm{A_1}]\,[\mathrm{A_1} - \mathrm{Hox_{A_1}}] \\
[\dot{A_2}] &= k_{-2}\,[\mathrm{A_2} - \mathrm{Pou}] - k_2\,[\mathrm{A_2}]\,[\mathrm{A_2} - \mathrm{Pou}] \\
[\dot{B_1}] &= k_{-3}\,[\mathrm{B} - \mathrm{Hox_B}] - k_3\,[\mathrm{B_1}]\,[\mathrm{B} - \mathrm{Hox_{B_1}}] \\
[\dot{B_2}] &= k_{-4}\,[\mathrm{B_2} - \mathrm{Pou}] - k_4\,[\mathrm{B_2}]\,[\mathrm{B_2} - \mathrm{Pou}] \\
[\dot{C}] &= k_{-5}\,[\mathrm{C} - \mathrm{Ebox}] - k_5\,[\mathrm{C}]\,[\mathrm{C} - \mathrm{Ebox}] \\
[\dot{R}] &= k_{-6}\,[\mathrm{P} - \mathrm{TATAbox}] - k_6\,[\mathrm{R}]\,[\mathrm{R} - \mathrm{TATAbox}]\,.
\end{aligned} \tag{F}$$

At the equilibrium, the concentration of the species remains constant:

$$[\dot{A_1}] = [\dot{A_2}] = [\dot{B_1}] = [\dot{B_2}] = [\dot{C}] = [\dot{R}] = 0. \tag{G}$$

Denoting the equilibrium dissociation constants of the species from the DNA $K_{A_1} = \frac{k_{-1}}{k_1}$, $K_{A_2} = \frac{k_{-2}}{k_2}$, $K_{B_1} = \frac{k_{-3}}{k_3}$, $K_{B_2} = \frac{k_{-4}}{k_4}$, $K_C = \frac{k_{-5}}{k_5}$ and $K_R = \frac{k_{-6}}{k_6}$, the reactions are described by the dissociation constants as:

$$K_{A_1} = \frac{[\mathrm{A_1}]\,[\mathrm{Hox_{A_1}}]}{[\mathrm{A_1} - \mathrm{Hox_{A_1}}]}$$

$$K_{A_2} = \frac{[\mathrm{A_2}]\,[\mathrm{Pou}]}{[\mathrm{A_2} - \mathrm{Pou}]}$$

$$K_{B_1} = \frac{[\mathrm{B_1}]\,[\mathrm{Hox_{B_1}}]}{[\mathrm{B_1} - \mathrm{Hox_{B_1}}]}$$

$$K_{B_2} = \frac{[\mathrm{B_2}]\,[\mathrm{Pou}]}{[\mathrm{B_2} - \mathrm{Pou}]}$$

$$K_C = \frac{[\mathrm{C}]\,[\mathrm{Ebox}]}{[\mathrm{C} - \mathrm{Ebox}]}$$

$$K_R = \frac{[\mathrm{R}]\,[\mathrm{TATAbox}]}{[\mathrm{R} - \mathrm{TATAbox}]}.$$

The probability that a binding site $i = \{\mathrm{Hox_{A_1}}, \mathrm{Hox_{B_1}}, \mathrm{Pou}, \mathrm{Ebox}, \mathrm{TATAbox}\}$ is occupied by a ligand $j = \{A_1, A_2, B_1, B_2, C, R\}$ can be expressed as the fraction of occupied sites. Representing the total number of binding sites as the sum of empty sites plus those occupied by the ligand, we can write:

$$P_{\mathrm{binding}}\left(\mathrm{A_1} - \mathrm{Hox_{A_1}}\right) = \frac{[\mathrm{A_1} - \mathrm{Hox_{A_1}}]}{[\mathrm{Hox_{A_1}}] + [\mathrm{A_1} - \mathrm{Hox_{A_1}}]}$$

$$P_{\mathrm{binding}}\left(\mathrm{A_2} - \mathrm{Pou}\right) = \frac{[\mathrm{A_2} - \mathrm{Pou}]}{[\mathrm{Pou}] + [\mathrm{A_2} - \mathrm{Pou}]}$$

$$P_{\mathrm{binding}}\left(\mathrm{B_1} - \mathrm{Hox_{B_1}}\right) = \frac{[\mathrm{B_1} - \mathrm{Hox_{B_1}}]}{[\mathrm{Hox_{B_1}}] + [\mathrm{B_1} - \mathrm{Hox_{B_1}}]}$$

$$P_{\mathrm{binding}}\left(\mathrm{B_2} - \mathrm{Pou}\right) = \frac{[\mathrm{B_2} - \mathrm{Pou}]}{[\mathrm{Pou}] + [\mathrm{B_2} - \mathrm{Pou}]}$$

$$P_{\mathrm{binding}}\left(\mathrm{C} - \mathrm{Ebox}\right) = \frac{[\mathrm{C} - \mathrm{Ebox}]}{[\mathrm{Ebox}] + [\mathrm{C} - \mathrm{Ebox}]}$$

$$P_{\mathrm{binding}}\left(\mathrm{R} - \mathrm{TATAbox}\right) = \frac{[\mathrm{R} - \mathrm{TATAbox}]}{[\mathrm{TATAbox}] + [\mathrm{R} - \mathrm{TATAbox}]}.$$

Using the concentrations of the substrates $[\mathrm{A}_1]$, $[\mathrm{A}_2]$, $[\mathrm{B}_1]$, $[\mathrm{B}_2]$, [C], [R], and the values of the dissociation constants, these probabilities at equilibrium assume the form of Hill equations:

$$P_{\text{binding}}\left(\mathrm{A}_1 - \mathrm{Hox}_{\mathrm{A}_1}\right) = \frac{\frac{[\mathrm{A}_1]}{K_{A_1}}}{1 + \frac{[\mathrm{A}_1]}{K_{A_1}}} = \frac{[\mathrm{A}_1]}{K_{A_1} + [\mathrm{A}_1]}$$

$$P_{\text{binding}}\left(\mathrm{A}_2 - \mathrm{Pou}\right) = \frac{\frac{[\mathrm{A}_2]}{K_{A_2}}}{1 + \frac{[\mathrm{A}_2]}{K_{A_2}}} = \frac{[\mathrm{A}_2]}{K_{A_2} + [\mathrm{A}_2]}$$

$$P_{\text{binding}}\left(\mathrm{B}_1 - \mathrm{Hox}_{\mathrm{B}_1}\right) = \frac{\frac{[\mathrm{B}_1]}{K_{B_1}}}{1 + \frac{[\mathrm{B}_1]}{K_{B_1}}} = \frac{[\mathrm{B}_1]}{K_{B_1} + [\mathrm{B}_1]}$$

$$P_{\text{binding}}\left(\mathrm{B}_2 - \mathrm{Pou}\right) = \frac{\frac{[\mathrm{B}_2]}{K_{B_2}}}{1 + \frac{[\mathrm{B}_2]}{K_{B_2}}} = \frac{[\mathrm{B}_2]}{K_{B_2} + [\mathrm{B}_2]}$$

$$P_{\text{binding}}\left(\mathrm{C} - \mathrm{Ebox}\right) = \frac{\frac{[\mathrm{C}]}{K_C}}{1 + \frac{[\mathrm{C}]}{K_C}} = \frac{[\mathrm{C}]}{K_C + [\mathrm{C}]}$$

$$P_{\text{binding}}\left(\mathrm{R} - \mathrm{TATAbox}\right) = \frac{\frac{[\mathrm{R}]}{K_R}}{1 + \frac{[\mathrm{R}]}{K_R}} = \frac{[\mathrm{R}]}{K_R + [\mathrm{R}]}.$$

In the expressions above, the dissociation constants K_{A_1}, K_{A_2}, K_{B_1}, K_{B_2}, K_C and K_R are naturally interpreted as the concentration of the ligand needed in order to have a $1/2$ probability of the receptor being occupied. We denote the ratio between the probability of each site of being bound v.s. unbound by the corresponding molecule as q_j. Then, for A_1, A_2, B_1, B_2, C and R these ratios are q_{A_1}, q_{A_2}, q_{B_1}, q_{B_2}, q_C and q_R. The probability that a molecule is bound to a specific region can be

described using these ratios as:

$$P_{\text{binding}}(\mathrm{A_1} - \mathrm{Hox_{A_1}}) = \frac{q_{A_1}}{1+q_{A_1}}$$
$$P_{\text{binding}}(\mathrm{A_2} - \mathrm{Pou}) = \frac{q_{A_2}}{1+q_{A_2}}$$
$$P_{\text{binding}}(\mathrm{B_1} - \mathrm{Hox_{B_1}}) = \frac{q_{B_1}}{1+q_{B_1}}$$
$$P_{\text{binding}}(\mathrm{B_2} - \mathrm{Pou}) = \frac{q_{B_2}}{1+q_{B_2}}$$
$$P_{\text{binding}}(\mathrm{C} - \mathrm{Ebox}) = \frac{q_{C}}{1+q_{C}}$$
$$P_{\text{binding}}(\mathrm{R} - \mathrm{TATAbox}) = \frac{q_{R}}{1+q_{R}}$$

where q_{A_1}, q_{A_2}, q_{B_1}, q_{B_2}, q_C and q_R can also be written as the ratios between the concentrations and dissociation constants:

$$q_{A_1} = \frac{[A_1]}{K_{A_1}}$$
$$q_{A_2} = \frac{[A_2]}{K_{A_2}}$$
$$q_{B_1} = \frac{[B_1]}{K_{B_1}}$$
$$q_{B_2} = \frac{[B_2]}{K_{B_2}}$$
$$q_{C} = \frac{[C]}{K_{C}}$$
$$q_{R} = \frac{[R]}{K_{R}}.$$

The DNA template and the TFs that take part in the regulation of transcription considered in this work lead to 48 possible molecular configurations, or distinct ways in which the system can be arranged, denoted σ_k, $k = 1, ..., 48$. A state is a configuration of the TFs and of the corresponding specific binding sites. For the four species (A, B, C, R), two of them with two distinct domains (A_1, A_2, B_1, B_2), and the five binding sites ($\mathrm{Hox_{A_1}}$, $\mathrm{Hox_{B_1}}$, Pou, Ebox, TATAbox), all 48 configurations σ_k are illustrated in Figures A and B.

Consider R molecules of RNAp, A molecules of Acj6, B molecules of Pdm3, C molecules of Fer1, and N_{ns} non-specific binding sites that they can be distributed on. For each molecule one can consider a first state in which all the A, B, C, or R are bound to non-specific sites, and a second possible state in which one of them is bound to the Or59b cluster while the remaining $A-1$, $B-1$, $C-1$, or $R-1$ bind nonspecifically. The total number of microstates available to the system is $\frac{N_{ns}!}{R!A!B!C!(N_{ns}-(R+A+B+C))}$. There are N_{ns} possible different places where one molecule

can bind to. When one molecule binds to DNA there will be $N_{ns} - 1$ choices for the remaining ones, and so on. The multiplicities account for the number of different ways of arranging $R!A!B!C!$ (or $(R-1)!A!B!C!$ if one R molecule is already bound) molecules in the DNA template.

Taking into account the occupancy distribution of the molecules on the target DNA sequence, one can assign probabilities to each of the molecular configurations σ_k. Each state σ_k is given a statistical weight or partial partition function p_k that is calculated from the interaction energies among bound molecules (coefficient J_{jn}), and from the interaction energy between each molecule and a specific region in the DNA, as a measure of the strength of the binding sites that they occupy in the configuration (ε_j^S). Additional factors might be introduced in p_k accounting for the epigenetic interactions (H_m) and will be explained later in detail. In brief, the three distinct classes of interactions the overall regulation can be decomposed into for our system are: (a) the interactions between TFs and the genomic sequence (TF-DNA), (b) the interactions among the TFs (TF-TF) and with the RNA polymerase (TF-RNAp), and (c) the interactions with the epigenome.

(a): TF-DNA. The binding energies of each TF and RNAp for specific and non-specific sites are denoted $\varepsilon^S_{A_1}, \varepsilon^S_{B_1}, \varepsilon^S_{A_2}, \varepsilon^S_{B_2}, \varepsilon^S_C, \varepsilon^S_R$ and $\varepsilon^{NS}_{A_1}, \varepsilon^{NS}_{B_2}, \varepsilon^{NS}_{A_1}, \varepsilon^{NS}_{B_2}, \varepsilon^{NS}_C, \varepsilon^{NS}_R$ respectively.

(b): TF-TF, TF-RNAp. Proteins with two domains such as Acj6 and Pdm3 are more stable when both domains are bound to the DNA. The parameter J_{jn} is a measure of the cooperativity and implies that when both sites are occupied, the energy is more than the sum of the individual binding energies [6, 10, 7]. Therefore, when either Acj6 or Pdm3 occupies the two motifs, an extra term, denoted respectively $J_{A_1A_2}$ and $J_{B_1B_2}$, is introduced. We assume that whenever the Pou and a Homeobox site are occupied at the same time by the same type of TF they are bound by the same protein. In other words, when an Acj6 or a Pdm3 is found attached to its Homeobox motif, this already bound TF is in any case the one that can bind Pou by means of its Pou domain, instead of a new free TF. So, everytime a Homeobox site and Pou site are bound by a TF of the same nature the J_{jn} term is taken into account.

The repressive effect on Pou from Acj6 or Pdm3 when one of them is bound to a Homeobox site and the other TF is in Pou, is denoted $\varepsilon_{A_1B_2}$ (Acj6 bound to Homeobox competes for the space when Pdm3 is bound to Pou) and $\varepsilon_{B_1A_2}$ (Pdm3 bound to Homeobox competes for the space when Acj6 is bound to Pou). The interaction coefficient of Fer1 with the RNAp is given by the activation energy ε_{CR}.

(c): Epigenetic interactions. The third type of interactions included in the model are of epigenetic nature. They are needed to describe the different behavior of the chromatin in the su(var)3-9 mutations. Under our assumption, when the chromatin is closed (normal chromatin state) Fer1 can bind Ebox only when there is a TF attached to the Pou site. However, with a su(var)3-9 mutant a TF bound to Pou is not strictly necessary for Fer1 binding. To describe the states in which Fer1 is bound to Ebox with no protein bound to Pou, the

epigenetic terms H_1 and H_2 are introduced. The parameter H_1 is used when there is a Fer1 bound to Ebox with no Acj6 nor Pdm3 bound to the entire cluster (i.e. states σ_{21} and σ_{45}). The parameter H_2 is used when there is a Fer1 bound to Ebox with no Acj6 nor Pdm3 bound to Pou (i.e. states σ_{22}, σ_{23}, σ_{24} and $\sigma_{46}, \sigma_{47}, \sigma_{48}$). The reason for treating these two cases differently is because one of these TFs attached to a Homeobox motif could hamper Fer1 binding. The states in which these terms appear are negligible in normal chromatin, but they become relevant when chromatin is trimethylated by mutant su(var)3-9.

The modification of the chromatin that follows a su(var)3-9 mutation also impacts the cooperativity coefficients $w_{A_1A_2}$ and $w_{B_1B_2}$. Two epigenetic factors h_A and h_B are introduced to modulate these terms, in particular their effect on transcription when Fer1 is bound to Ebox (i.e., states σ_{14}, σ_{15}, σ_{38}, σ_{39} for h_A and σ_{13}, σ_{16}, σ_{37}, σ_{40} for h_B).

A final epigenetic interaction might be of relevance. When chromatin is trimethylated by mutant su(var)3-9, the concentration of methyltransferases in the cell is almost halved, so more Pdm3 and Acj6 is available in the cell, as there are less methyltransferases to form the complex that opens the chromatin. Consequently, Acj6 or Pdm3 may be more frequently attached to Pou, and their detachment for forming the methyltransferase-TF complex could happen less frequently. Then, TFs bound to Pou may induce stronger physical competition for Fer1. This effect caused by the availability of Acj6 and Pdm3 is expressed as a term H_3 and linked to the use of mutant su(var)3-9 in the experiments. When a mutant is used, the spatial competition between Acj6 and Pdm3 with Fer1 may become significant. Although sometimes decreasing the level of the enzymes that trimethylate the histones implies a complete change in the regulatory balance of the cell and TFs behavior, only the cases in which TFs maintain their roles are addressed in this work.

Taking into account all these factors, the partial partition functions p_k or unnormalized statistical weights of each state σ_k can be written as

$$
\begin{aligned}
p_1 &= Z\left(R, A_1, A_2, B_1, B_2, C\right) \\
p_2 &= Z\left(R, A_1, A_2, B_1, B_2-1, C\right) \cdot e^{-\beta \varepsilon^S_{B_2}} \\
p_3 &= Z\left(R, A_1, A_2-1, B_1, B_2, C\right) \cdot e^{-\beta \varepsilon^S_{A_2}} \\
p_4 &= Z\left(R, A_1-1, A_2, B_1-1, B_2-1, C\right) \cdot e^{-\beta\left(\varepsilon^S_{A_1}+\varepsilon^S_{B_1}+\varepsilon^S_{B_2}+J_{B_1B_2}+\varepsilon_{A_1B_2}\right)} \\
p_5 &= Z\left(R, A_1-1, A_2-1, B_1-1, B_2, C\right) \cdot e^{-\beta\left(\varepsilon^S_{A_1}+\varepsilon^S_{A_2}+J_{A_1A_2}+\varepsilon^S_{B_1}+\varepsilon_{B_1A_2}\right)} \\
p_6 &= Z\left(R, A_1-1, A_2, B_1-1, B_2, C\right) \cdot e^{-\beta\left(\varepsilon^S_{A_1}+\varepsilon^S_{B_1}\right)} \\
p_7 &= Z\left(R, A_1, A_2-1, B_1-1, B_2, C\right) \cdot e^{-\beta\left(\varepsilon^S_{B_1}+\varepsilon^S_{A_2}+\varepsilon_{B_1A_2}\right)} \\
p_8 &= Z\left(R, A_1-1, A_2, B_1, B_2-1, C\right) \cdot e^{-\beta\left(\varepsilon^S_{A_1}+\varepsilon^S_{B_2}+\varepsilon_{A_1B_2}\right)} \\
p_9 &= Z\left(R, A_1, A_2, B_1-1, B_2-1, C\right) \cdot e^{-\beta\left(\varepsilon^S_{B_1}+\varepsilon^S_{B_2}+J_{B_1B_2}\right)} \\
p_{10} &= Z\left(R, A_1-1, A_2-1, B_1, B_2, C\right) \cdot e^{-\beta\left(\varepsilon^S_{A_1}+\varepsilon^S_{A_2}+J_{A_1A_2}\right)} \\
p_{11} &= Z\left(R, A_1-1, A_2, B_1, B_2, C\right) \cdot e^{-\beta \varepsilon^S_{A_1}} \\
p_{12} &= Z\left(R, A_1, A_2, B_1-1, B_2, C\right) \cdot e^{-\beta \varepsilon^S_{B_1}} \\
p_{13} &= Z\left(R, A_1, A_2, B_1-1, B_2-1, C-1\right) \cdot e^{-\beta\left(\varepsilon^S_{B_1}+\varepsilon^S_{B_2}+J_{B_1B_2}+\varepsilon^S_{C}+H_3+H_B\right)} \\
p_{14} &= Z\left(R, A_1-1, A_2-1, B_1, B_2, C-1\right) \cdot e^{-\beta\left(\varepsilon^S_{A_1}+\varepsilon^S_{A_2}+J_{A_1A_2}+\varepsilon^S_{C}+H_3+H_A\right)} \\
p_{15} &= Z\left(R, A_1-1, A_2-1, B_1-1, B_2, C-1\right) \cdot e^{-\beta\left(\varepsilon^S_{A_1}+\varepsilon^S_{A_2}+J_{A_1A_2}+\varepsilon^S_{B_1}+\varepsilon^S_{C}+\varepsilon_{B_1A_2}+H_3+H_A\right)} \\
p_{16} &= Z\left(R, A_1-1, A_2, B_1-1, B_2-1, C-1\right) \cdot e^{-\beta\left(\varepsilon^S_{A_1}+\varepsilon^S_{B_1}+J_{B_1B_2}+\varepsilon^S_{B_2}+\varepsilon^S_{C}+\varepsilon_{A_1B_2}+H_3+H_B\right)} \\
p_{17} &= Z\left(R, A_1, A_2, B_1, B_2-1, C-1\right) \cdot e^{-\beta\left(\varepsilon^S_{B_2}+\varepsilon^S_{C}+H_3\right)} \\
p_{18} &= Z\left(R, A_1, A_2-1, B_1, B_2, C-1\right) \cdot e^{-\beta\left(\varepsilon^S_{A_2}+\varepsilon^S_{C}+H_3\right)} \\
p_{19} &= Z\left(R, A_1-1, A_2, B_1, B_2-1, C-1\right) \cdot e^{-\beta\left(\varepsilon^S_{A_1}+\varepsilon^S_{B_2}+\varepsilon^S_{C}+\varepsilon_{A_1B_2}+H_3\right)} \\
p_{20} &= Z\left(R, A_1, A_2-1, B_1-1, B_2, C-1\right) \cdot e^{-\beta\left(\varepsilon^S_{B_1}+\varepsilon^S_{A_2}+\varepsilon^S_{C}+\varepsilon_{B_1A_2}+H_3\right)} \\
p_{21} &= Z\left(R, A_1, A_2, B_1, B_2, C-1\right) \cdot e^{-\beta\left(\varepsilon^S_{C}+H_1\right)} \\
p_{22} &= Z\left(R, A_1, A_2, B_1-1, B_2, C-1\right) \cdot e^{-\beta\left(\varepsilon^S_{B_1}+\varepsilon^S_{C}+H_2\right)} \\
p_{23} &= Z\left(R, A_1-1, A_2, B_1, B_2, C-1\right) \cdot e^{-\beta\left(\varepsilon^S_{A_1}+\varepsilon^S_{C}+H_2\right)} \\
p_{24} &= Z\left(R, A_1-1, A_2, B_1-1, B_2, C-1\right) \cdot e^{-\beta\left(\varepsilon^S_{A_1}+\varepsilon^S_{B_1}+\varepsilon^S_{C}+H_2\right)}
\end{aligned}
$$

$$p_{25} = Z\left(R-1, A_1, A_2, B_1, B_2, C\right) \cdot e^{-\beta \varepsilon_R^S}$$
$$p_{26} = Z\left(R-1, A_1, A_2, B_1, B_2-1, C\right) \cdot e^{-\beta\left(\varepsilon_{B_2}^S + \varepsilon_R^S\right)}$$
$$p_{27} = Z\left(R-1, A_1, A_2-1, B_1, B_2, C\right) \cdot e^{-\beta\left(\varepsilon_{A_2}^S + \varepsilon_R^S\right)}$$
$$p_{28} = Z\left(R-1, A_1-1, A_2, B_1-1, B_2-1, C\right) \cdot e^{-\beta\left(\varepsilon_{A_1}^S + \varepsilon_{B_1}^S + \varepsilon_{B_2}^S + J_{B_1 B_2} + \varepsilon_R^S + \varepsilon_{A_1 B_2}\right)}$$
$$p_{29} = Z\left(R-1, A_1-1, A_2-1, B_1-1, B_2, C\right) \cdot e^{-\beta\left(\varepsilon_{A_1}^S + \varepsilon_{A_2}^S + J_{A_1 A_2} + \varepsilon_{B_1}^S + \varepsilon_R^S + \varepsilon_{B_1 A_2}\right)}$$
$$p_{30} = Z\left(R-1, A_1-1, A_2, B_1-1, B_2, C\right) \cdot e^{-\beta\left(\varepsilon_{A_1}^S + \varepsilon_{B_1}^S + \varepsilon_R^S\right)}$$
$$p_{31} = Z\left(R-1, A_1, A_2-1, B_1-1, B_2, C\right) \cdot e^{-\beta\left(\varepsilon_{B_1}^S + \varepsilon_{A_2}^S + \varepsilon_R^S + \varepsilon_{B_1 A_2}\right)}$$
$$p_{32} = Z\left(R-1, A_1-1, A_2, B_1, B_2-1, C\right) \cdot e^{-\beta\left(\varepsilon_{A_1}^S + \varepsilon_{B_2}^S + \varepsilon_R^S + \varepsilon_{A_1 B_2}\right)}$$
$$p_{33} = Z\left(R-1, A_1, A_2, B_1-1, B_2-1, C\right) \cdot e^{-\beta\left(\varepsilon_{B_1}^S + \varepsilon_{B_2}^S + J_{B_1 B_2} + \varepsilon_R^S\right)}$$
$$p_{34} = Z\left(R-1, A_1-1, A_2-1, B_1, B_2, C\right) \cdot e^{-\beta\left(\varepsilon_{A_1}^S + \varepsilon_{A_2}^S + J_{A_1 A_2} + \varepsilon_R^S\right)}$$
$$p_{35} = Z\left(R-1, A_1-1, A_2, B_1, B_2, C\right) \cdot e^{-\beta\left(\varepsilon_{A_1}^S + \varepsilon_R^S\right)}$$
$$p_{36} = Z\left(R-1, A_1, A_2, B_1-1, B_2, C\right) \cdot e^{-\beta\left(\varepsilon_{B_1}^S + \varepsilon_R^S\right)}$$
$$p_{37} = Z\left(R-1, A_1, A_2, B_1-1, B_2-1, C-1\right) \cdot e^{-\beta\left(\varepsilon_{B_1}^S + \varepsilon_{B_2}^S + J_{B_1 B_2} + \varepsilon_C^S + \varepsilon_R^S + \varepsilon_{CR} + H_3 + H_B\right)}$$
$$p_{38} = Z\left(R-1, A_1-1, A_2-1, B_1, B_2, C-1\right) \cdot e^{-\beta\left(\varepsilon_{A_1}^S + \varepsilon_{A_2}^S + J_{A_1 A_2} + \varepsilon_C^S + \varepsilon_R^S + \varepsilon_{CR} + H_3 + H_A\right)}$$
$$p_{39} = Z\left(R-1, A_1-1, A_2-1, B_1-1, B_2, C-1\right) \cdot e^{-\beta\left(\varepsilon_{A_1}^S + \varepsilon_{A_2}^S + J_{A_1 A_2} + \varepsilon_{B_1}^S + \varepsilon_C^S + \varepsilon_R^S + \varepsilon_{CR} + \varepsilon_{B_1 A_2} + H_3 + H_A\right)}$$
$$p_{40} = Z\left(R-1, A_1-1, A_2, B_1-1, B_2-1, C-1\right) \cdot e^{-\beta\left(\varepsilon_{A_1}^S + \varepsilon_{B_1}^S + J_{B_1 B_2} + \varepsilon_{B_2}^S + \varepsilon_C^S + \varepsilon_R^S + \varepsilon_{CR} + \varepsilon_{A_1 B_2} + H_3 + H_B\right)}$$
$$p_{41} = Z\left(R-1, A_1, A_2, B_1, B_2-1, C-1\right) \cdot e^{-\beta\left(\varepsilon_{B_2}^S + \varepsilon_C^S + \varepsilon_R^S + \varepsilon_{CR} + H_3\right)}$$
$$p_{42} = Z\left(R-1, A_1, A_2-1, B_1, B_2, C-1\right) \cdot e^{-\beta\left(\varepsilon_{A_2}^S + \varepsilon_C^S + \varepsilon_R^S + \varepsilon_{CR} + H_3\right)}$$
$$p_{43} = Z\left(R-1, A_1-1, A_2, B_1-1, B_2, C-1\right) \cdot e^{-\beta\left(\varepsilon_{A_1}^S + \varepsilon_{B_2}^S + \varepsilon_C^S + \varepsilon_R^S + \varepsilon_{CR} + \varepsilon_{A_1 B_2} + H_3\right)}$$
$$p_{44} = Z\left(R-1, A_1, A_2-1, B_1-1, B_2, C-1\right) \cdot e^{-\beta\left(\varepsilon_{B_1}^S + \varepsilon_{A_2}^S + \varepsilon_C^S + \varepsilon_R^S + \varepsilon_{CR} + \varepsilon_{B_1 A_2} + H_3\right)}$$
$$p_{45} = Z\left(R-1, A_1, A_2, B_1, B_2, C-1\right) \cdot e^{-\beta\left(\varepsilon_C^S + \varepsilon_R^S + \varepsilon_{CR} + H_1\right)}$$
$$p_{46} = Z\left(R-1, A_1, A_2, B_1-1, B_2, C-1\right) \cdot e^{-\beta\left(\varepsilon_{B_1}^S + \varepsilon_C^S + \varepsilon_R^S + \varepsilon_{CR} + H_2\right)}$$
$$p_{47} = Z\left(R-1, A_1-1, A_2, B_1, B_2, C-1\right) \cdot e^{-\beta\left(\varepsilon_{A_1}^S + \varepsilon_C^S + \varepsilon_R^S + \varepsilon_{CR} + H_2\right)}$$
$$p_{48} = Z\left(R-1, A_1-1, A_2, B_1-1, B_2, C-1\right) \cdot e^{-\beta\left(\varepsilon_{A_1}^S + \varepsilon_{B_1}^S + \varepsilon_C^S + \varepsilon_R^S + \varepsilon_{CR} + H_2\right)}.$$

As long as $N_{ns} \gg (R + A' + B' + C)$, the total number of microstates can be simplified using the approximation $\frac{N_{ns}!}{R!A_1!A_2!B_1!B_2!C!(N_{ns}-(R+A'+B'+C))} \approx N_{ns}^{(R+A'+B'+C)}$. For the statistical weights listed above, the distribution of molecules on the nonspecific DNA are characterized by the Z terms. The energies correspond to the difference between specific and nonspecific binding for RNA polymerase, Acj6, Pdm3 and Fer1, under the assumption of a homogeneous nonspecific background, i..e, that binding energies differences among nonspecific sites can be neglected. Under such assumption, the Z term for the configuration where there are no molecules bound to DNA is:

$$Z\left(R, A_1, A_2, B_1, B_2, C\right) = \frac{N_{ns}!}{R!A_1!A_2!B_1!B_2!C!\left(N_{ns} - P - A' - B' - C\right)!} \cdot e^{-\beta\left(P \cdot \varepsilon_R^{NS} + A' \cdot \varepsilon_A^{NS} + B' \cdot \varepsilon_B^{NS} + C \cdot \varepsilon_C^{NS}\right)}.$$

Since $N_{ns} \gg (R + A' + B' + C)$, this is approximated as

$$Z(R, A_1, A_2, B_1, B_2, C) = \frac{N_{ns}^{\left(R+A'+B'+C\right)}}{R!A_1!A_2!B_1!B_2!C!} \cdot e^{-\beta\left(P\cdot\varepsilon_R^{NS}+A'\cdot\varepsilon_A^{NS}+B'\cdot\varepsilon_B^{NS}+C\cdot\varepsilon_C^{NS}\right)}.$$

Analogously, for the remaining configurations:

$$Z(R, A_1, A_2, B_1, B_2 - 1, C) = \frac{N_{ns}^{\left(R+A'+B_1+B_2-1+C\right)}}{R!A_1!A_2!B_1!\,(B_2-1)!C!} \cdot e^{-\beta\left(P\cdot\varepsilon_R^{NS}+A'\cdot\varepsilon_A^{NS}+B_1\cdot\varepsilon_B^{NS}+(B_2-1)\cdot\varepsilon_B^{NS}+C\cdot\varepsilon_C^{NS}\right)}$$

$\vdots$

$$Z(R-1, A_1-1, A_2, B_1-1, B_2, C-1) = \frac{N_{ns}^{(R-1+A_1-1+A_2,B_1-1+B_2+C-1)}}{(R-1)!\,(A-1)!\,(B-1)!\,(C-1)!} \cdot e^{S_{48}}$$

with

$$S_{48} = -\beta\left((R-1)\cdot\varepsilon_R^{NS} + (A_1-1)\cdot\varepsilon_A^{NS} + A_2\cdot\varepsilon_A^{NS} + (B_1-1)\cdot\varepsilon_B^{NS} + B_2\cdot\varepsilon_B^{NS} + (C-1)\cdot\varepsilon_C^{NS}\right).$$

If we normalize each statistical weight by p_1, the new statistical weights are:

$$
\begin{aligned}
p_1 &= 1\\
p_2 &= \frac{B_2}{N_{ns}} \cdot e^{-\beta \Delta\varepsilon_{B_2}}\\
p_3 &= \frac{A_2}{N_{ns}} \cdot e^{-\beta \Delta\varepsilon_{A_2}}\\
p_4 &= \frac{A_1 \cdot B_1 \cdot B_2}{N_{ns}^3} \cdot e^{-\beta\left(\Delta\varepsilon_{A_1}+\Delta\varepsilon_{B_1}+\Delta\varepsilon_{B_2}+J_{B_1B_2}+\varepsilon_{A_1B_2}\right)}\\
p_5 &= \frac{A_1 \cdot A_2 \cdot B_1}{N_{ns}^3} \cdot e^{-\beta\left(\Delta\varepsilon_{A_1}+\Delta\varepsilon_{A_2}+\Delta\varepsilon_{B_1}+J_{A_1A_2}+\varepsilon_{B_1A_2}\right)}\\
p_6 &= \frac{A_1 \cdot B_1}{N_{ns}^2} \cdot e^{-\beta\cdot\left(\Delta\varepsilon_{A_1}+\Delta\varepsilon_{B_1}\right)}\\
p_7 &= \frac{B_1 \cdot A_2}{N_{ns}^2} \cdot e^{-\beta\left(\Delta\varepsilon_{B_1}+\Delta\varepsilon_{A_2}+\varepsilon_{B_1A_2}\right)}\\
p_8 &= \frac{A_1 \cdot B_2}{N_{ns}^2} \cdot e^{-\beta\left(\Delta\varepsilon_{A_1}+\Delta\varepsilon_{B_2}+\varepsilon_{A_1B_2}\right)}\\
p_9 &= \frac{B_1 \cdot B_2}{N_{ns}^2} \cdot e^{-\beta\left(\Delta\varepsilon_{B_1}+\Delta\varepsilon_{B_2}+J_{B_1B_2}\right)}\\
p_{10} &= \frac{A_1 \cdot A_2}{N_{ns}^2} \cdot e^{-\beta\left(\Delta\varepsilon_{A_1}+\Delta\varepsilon_{A_2}+J_{A_1A_2}\right)}\\
p_{11} &= \frac{A_1}{N_{ns}} \cdot e^{-\beta \Delta\varepsilon_{A_1}}\\
p_{12} &= \frac{B_2}{N_{ns}} \cdot e^{-\beta \Delta\varepsilon_{B_1}}\\
p_{13} &= \frac{B_1 \cdot B_2 \cdot C}{N_{ns}^3} \cdot e^{-\beta\left(\Delta\varepsilon_{B_1}+\Delta\varepsilon_{B_2}+J_{B_1B_2}+\Delta\varepsilon_C+H_3+H_B\right)}\\
p_{14} &= \frac{A_1 \cdot A_2 \cdot C}{N_{ns}^3} \cdot e^{-\beta\left(\Delta\varepsilon_{A_1}+\Delta\varepsilon_{A_2}+J_{A_1A_2}+\Delta\varepsilon_C+H_3+H_A\right)}\\
p_{15} &= \frac{A_1 \cdot A_2 \cdot B_1 \cdot C}{N_{ns}^4} \cdot e^{-\beta\left(\Delta\varepsilon_{A_1}+\Delta\varepsilon_{A_2}+J_{A_1A_2}+\Delta\varepsilon_{B_1}+\Delta\varepsilon_C+\varepsilon_{B_1A_2}+H_3+H_A\right)}\\
p_{16} &= \frac{A_1 \cdot B_1 \cdot B_2 \cdot C}{N_{ns}^4} \cdot e^{-\beta\left(\Delta\varepsilon_{A_1}+\Delta\varepsilon_{B_1}+\Delta\varepsilon_{B_2}+J_{B_1B_2}+\Delta\varepsilon_C+\varepsilon_{A_1B_2}+H_3+H_B\right)}\\
p_{17} &= \frac{B_2 \cdot C}{N_{ns}^2} \cdot e^{-\beta\left(\Delta\varepsilon_C+\Delta\varepsilon_{B_2}+H_3\right)}\\
p_{18} &= \frac{A_2 \cdot C}{N_{ns}^2} \cdot e^{-\beta\left(\Delta\varepsilon_C+\Delta\varepsilon_{A_2}+H_3\right)}\\
p_{19} &= \frac{A_1 \cdot B_2 \cdot C}{N_{ns}^3} \cdot e^{-\beta\left(\Delta\varepsilon_C+\Delta\varepsilon_{A_1}+\Delta\varepsilon_{B_2}+\varepsilon_{A_1B_2}+H_3\right)}\\
p_{20} &= \frac{A_2 \cdot B_1 \cdot C}{N_{ns}^3} \cdot e^{-\beta\left(\Delta\varepsilon_C+\Delta\varepsilon_{B_1}+\Delta\varepsilon_{A_2}+\varepsilon_{B_1A_2}+H_3\right)}\\
p_{21} &= \frac{C}{N_{ns}} \cdot e^{-\beta(\Delta\varepsilon_C+H_1)}\\
p_{22} &= \frac{B_1 \cdot C}{N_{ns}^2} \cdot e^{-\beta\left(\Delta\varepsilon_C+\Delta\varepsilon_{B_1}+H_2\right)}\\
p_{23} &= \frac{A_1 \cdot C}{N_{ns}^2} \cdot e^{-\beta\left(\Delta\varepsilon_C+\Delta\varepsilon_{A_1}+H_2\right)}\\
p_{24} &= \frac{A_1 \cdot B_1 \cdot C}{N_{ns}^3} \cdot e^{-\beta\left(\Delta\varepsilon_C+\Delta\varepsilon_{A_1}+\Delta\varepsilon_{B_1}+H_2\right)}
\end{aligned}
$$

$$p_{25} = \frac{R}{N_{ns}} \cdot e^{-\beta \Delta\varepsilon_R}$$
$$p_{26} = \frac{B_2 \cdot R}{N_{ns}^2} \cdot e^{-\beta\left(\Delta\varepsilon_{B_2}+\Delta\varepsilon_R\right)}$$
$$p_{27} = \frac{A_2 \cdot R}{N_{ns}^2} \cdot e^{-\beta\left(\Delta\varepsilon_{A_2}+\Delta\varepsilon_R\right)}$$
$$p_{28} = \frac{A_1 \cdot B_1 \cdot B_2 \cdot R}{N_{ns}^4} \cdot e^{-\beta\left(\Delta\varepsilon_{A_1}+\Delta\varepsilon_{B_1}+\Delta\varepsilon_{B_2}+J_{B_1B_2}+\varepsilon_{A_1B_2}+\Delta\varepsilon_R\right)}$$
$$p_{29} = \frac{A_1 \cdot A_2 \cdot B_1 \cdot R}{N_{ns}^4} \cdot e^{-\beta\left(\Delta\varepsilon_{A_1}+\Delta\varepsilon_{A_2}+\Delta\varepsilon_{B_1}+J_{A_1A_2}+\varepsilon_{B_1A_2}+\Delta\varepsilon_R\right)}$$
$$p_{30} = \frac{A_1 \cdot B_1 \cdot R}{N_{ns}^3} \cdot e^{-\beta\cdot\left(\Delta\varepsilon_{A_1}+\Delta\varepsilon_{B_1}+\Delta\varepsilon_R\right)}$$
$$p_{31} = \frac{B_1 \cdot A_2 \cdot R}{N_{ns}^3} \cdot e^{-\beta\left(\Delta\varepsilon_{B_1}+\Delta\varepsilon_{A_2}+\varepsilon_{B_1A_2}+\Delta\varepsilon_R\right)}$$
$$p_{32} = \frac{A_1 \cdot B_2 \cdot R}{N_{ns}^3} \cdot e^{-\beta\left(\Delta\varepsilon_{A_1}+\Delta\varepsilon_{B_2}+\varepsilon_{A_1B_2}+\Delta\varepsilon_R\right)}$$
$$p_{33} = \frac{B_1 \cdot B_2 \cdot R}{N_{ns}^3} \cdot e^{-\beta\left(\Delta\varepsilon_{B_1}+\Delta\varepsilon_{B_2}+J_{B_1B_2}+\Delta\varepsilon_R\right)}$$
$$p_{34} = \frac{A_1 \cdot A_2 \cdot R}{N_{ns}^3} \cdot e^{-\beta\left(\Delta\varepsilon_{A_1}+\Delta\varepsilon_{A_2}+J_{A_1A_2}+\Delta\varepsilon_R\right)}$$
$$p_{35} = \frac{A_1 \cdot R}{N_{ns}^2} \cdot e^{-\beta\left(\Delta\varepsilon_{A_1}+\Delta\varepsilon_R\right)}$$
$$p_{36} = \frac{B_1 \cdot R}{N_{ns}^2} \cdot e^{-\beta\left(\Delta\varepsilon_{B_1}+\Delta\varepsilon_R\right)}$$
$$p_{37} = \frac{B_1 \cdot B_2 \cdot C \cdot R}{N_{ns}^4} \cdot e^{-\beta\left(\Delta\varepsilon_{B_1}+\Delta\varepsilon_{B_2}+J_{B_1B_2}+\Delta\varepsilon_C+\Delta\varepsilon_R+\varepsilon_{CR}+H_3+H_B\right)}$$
$$p_{38} = \frac{A_1 \cdot A_2 \cdot C \cdot R}{N_{ns}^4} \cdot e^{-\beta\left(\Delta\varepsilon_{A_1}+\Delta\varepsilon_{A_2}+J_{A_1A_2}+\Delta\varepsilon_C+\Delta\varepsilon_R+\varepsilon_{CR}+H_3+H_A\right)}$$
$$p_{39} = \frac{A_1 \cdot A_2 \cdot B_1 \cdot C \cdot R}{N_{ns}^5} \cdot e^{-\beta\left(\Delta\varepsilon_{A_1}+\Delta\varepsilon_{A_2}+J_{A_1A_2}+\Delta\varepsilon_{B_1}+\Delta\varepsilon_C+\varepsilon_{B_1A_2}+\Delta\varepsilon_R+\varepsilon_{CR}+H_3+H_A\right)}$$
$$p_{40} = \frac{A_1 \cdot B_1 \cdot B_2 \cdot C \cdot R}{N_{ns}^5} \cdot e^{-\beta\left(\Delta\varepsilon_{A_1}+\Delta\varepsilon_{B_1}+\Delta\varepsilon_{B_2}+J_{B_1B_2}+\Delta\varepsilon_C+\varepsilon_{A_1B_2}+\Delta\varepsilon_R+\varepsilon_{CR}+H_3+H_B\right)}$$
$$p_{41} = \frac{B_2 \cdot C \cdot R}{N_{ns}^3} \cdot e^{-\beta\left(\Delta\varepsilon_C+\Delta\varepsilon_{B_2}+H_3+\varepsilon_{CR}+\Delta\varepsilon_R\right)}$$
$$p_{42} = \frac{A_2 \cdot C \cdot R}{N_{ns}^3} \cdot e^{-\beta\left(\Delta\varepsilon_C+\Delta\varepsilon_{A_2}+H_3+\varepsilon_{CR}+\Delta\varepsilon_R\right)}$$
$$p_{43} = \frac{A_1 \cdot B_2 \cdot C \cdot R}{N_{ns}^4} \cdot e^{-\beta\left(\Delta\varepsilon_C+\Delta\varepsilon_{A_1}+\Delta\varepsilon_{B_2}+\varepsilon_{A_1B_2}+H_3+\Delta\varepsilon_R+\varepsilon_{CR}\right)}$$
$$p_{44} = \frac{A_2 \cdot B_1 \cdot C \cdot R}{N_{ns}^4} \cdot e^{-\beta\left(\Delta\varepsilon_C+\Delta\varepsilon_{B_1}+\Delta\varepsilon_{A_2}+\varepsilon_{B_1A_2}+H_3+\Delta\varepsilon_R+\varepsilon_{CR}\right)}$$
$$p_{45} = \frac{C \cdot R}{N_{ns}^2} \cdot e^{-\beta(\Delta\varepsilon_C+\Delta\varepsilon_R+\varepsilon_{CR}+H_1)}$$
$$p_{46} = \frac{B_1 \cdot C \cdot R}{N_{ns}^3} \cdot e^{-\beta\left(\Delta\varepsilon_C+\Delta\varepsilon_{B_1}+\Delta\varepsilon_R+\varepsilon_{CR}+H_2\right)}$$
$$p_{47} = \frac{A_1 \cdot C \cdot R}{N_{ns}^3} \cdot e^{-\beta\left(\Delta\varepsilon_C+\Delta\varepsilon_{A_1}+\Delta\varepsilon_R+\varepsilon_{CR}+H_2\right)}$$
$$p_{48} = \frac{A_1 \cdot B_1 \cdot C \cdot R}{N_{ns}^4} \cdot e^{-\beta\left(\Delta\varepsilon_C+\Delta\varepsilon_{A_1}+\Delta\varepsilon_{B_1}+\Delta\varepsilon_R+\varepsilon_{CR}+H_2\right)}$$

1.3 Probabilities of σ_k and model parameters

To simplify the expressions of the p_k and the parameter fitting, it is convenient to re-express the relative statistical weight p_k in terms of novel parameters, incorporating the exponentials of the energies.

The energies correspond to the difference between specific and nonspecific binding for RNA polymerase, Acj6, Pdm3 and Fer1, under the assumption of a homogeneous nonspecific background. The q_j terms in the model are equivalent to the energies as follows:

$$
\begin{aligned}
q_R &= \frac{R}{N_{ns}} \cdot e^{-\beta\Delta\varepsilon_R} \\
q_{A_1} &= \frac{A_1}{N_{ns}} \cdot e^{-\beta\Delta\varepsilon_{A_1}} \\
q_{A_2} &= \frac{A_2}{N_{ns}} \cdot e^{-\beta\Delta\varepsilon_{A_2}} \\
q_{B_1} &= \frac{B_1}{N_{ns}} \cdot e^{-\beta\Delta\varepsilon_{B_1}} \\
q_{B_2} &= \frac{B_2}{N_{ns}} \cdot e^{-\beta\Delta\varepsilon_{B_2}} \\
q_C &= \frac{C}{N_{ns}} \cdot e^{-\beta\Delta\varepsilon_C}
\end{aligned}
$$

while for the TF-TF and TF-RNAp interaction terms we have

$$
\begin{aligned}
w_{A_1A_2} &= e^{-\beta J_{A_1A_2}} \\
w_{B_1B_2} &= e^{-\beta J_{B_1B_2}} \\
w_{A_1B_2} &= e^{-\beta\varepsilon_{A_1B_2}} \\
w_{B_1A_2} &= e^{-\beta\varepsilon_{B_1A_2}} \\
w_{CR} &= e^{-\beta\varepsilon_{CR}}
\end{aligned}
\tag{H}
$$

and for the epigenetic terms

$$
\begin{aligned}
h_1 &= e^{-\beta H_1} \\
h_2 &= e^{-\beta H_2} \\
h_3 &= e^{-\beta H_3} \\
h_A &= e^{-\beta H_A} \\
h_B &= e^{-\beta H_B}.
\end{aligned}
$$

Expressing p_k directly in terms of q_j, w_{jn} and h_m yields:

$$
\begin{aligned}
p_1 &= 1 \\
p_2 &= q_{B_2} \\
p_3 &= q_{A_2} \\
p_4 &= q_{A_1} \cdot q_{B_1} \cdot q_{B_2} \cdot w_{B_1B_2} \cdot w_{A_1B_2} \\
p_5 &= q_{B_1} \cdot q_{A_1} \cdot q_{A_2} \cdot w_{A_1A_2} \cdot w_{B_1A_2} \\
p_6 &= q_{A_1} \cdot q_{B_1} \\
p_7 &= q_{B_1} \cdot q_{A_2} \cdot w_{B_1A_2} \\
p_8 &= q_{A_1} \cdot q_{B_2} \cdot w_{A_1B_2} \\
p_9 &= q_{B_1} \cdot q_{B_2} \cdot w_{B_1B_2} \\
p_{10} &= q_{A_1} \cdot q_{A_2} \cdot w_{A_1A_2} \\
p_{11} &= q_{A_1} \\
p_{12} &= q_{B_1} \\
p_{13} &= q_{B_1} \cdot q_{B_2} \cdot q_C \cdot w_{B_1B_2} \cdot h_3 \cdot h_B \\
p_{14} &= q_{A_1} \cdot q_{A_2} \cdot q_C \cdot w_{A_1A_2} \cdot h_3 \cdot h_A \\
p_{15} &= q_{A_1} \cdot q_{A_2} \cdot q_{B_1} \cdot q_C \cdot w_{A_1A_2} \cdot w_{B_1A_2} \cdot h_3 \cdot h_A \\
p_{16} &= q_{B_1} \cdot q_{B_2} \cdot q_{A_1} \cdot q_C \cdot w_{B_1B_2} \cdot w_{A_1B_2} \cdot h_3 \cdot h_B \\
p_{17} &= q_{B_2} \cdot q_C \cdot h_3 \\
p_{18} &= q_{A_2} \cdot q_C \cdot h_3 \\
p_{19} &= q_{A_1} \cdot q_{B_2} \cdot q_C \cdot w_{A_1B_2} \cdot h_3 \\
p_{20} &= q_{B_1} \cdot q_{A_2} \cdot q_C \cdot w_{B_1A_2} \cdot h_3 \\
p_{21} &= q_C \cdot h_1 \\
p_{22} &= q_{B_1} \cdot q_C \cdot h_2 \\
p_{23} &= q_{A_1} \cdot q_C \cdot h_2 \\
p_{24} &= q_{A_1} \cdot q_{B_1} \cdot q_C \cdot h_2
\end{aligned}
\tag{I}
$$

$$
\begin{aligned}
p_{25} &= q_R \\
p_{26} &= q_R \cdot q_{B_2} \\
p_{27} &= q_R \cdot q_{A_2} \\
p_{28} &= q_R \cdot q_{A_1} \cdot q_{B_1} \cdot q_{B_2} \cdot w_{B_1 B_2} \cdot w_{A_1 B_2} \\
p_{29} &= q_R \cdot q_{B_1} \cdot q_{A_1} \cdot q_{A_2} \cdot w_{A_1 A_2} \cdot w_{B_1 A_2} \\
p_{30} &= q_R \cdot q_{A_1} \cdot q_{B_1} \\
p_{31} &= q_R \cdot q_{B_1} \cdot q_{A_2} \cdot w_{B_1 A_2} \\
p_{32} &= q_R \cdot q_{A_1} \cdot q_{B_2} \cdot w_{A_1 B_2} \\
p_{33} &= q_R \cdot q_{B_1} \cdot q_{B_2} \cdot w_{B_1 B_2} \\
p_{34} &= q_R \cdot q_{A_1} \cdot q_{A_2} \cdot w_{A_1 A_2} \\
p_{35} &= q_R \cdot q_{A_1} \\
p_{36} &= q_R \cdot q_{B_1} \\
p_{37} &= q_R \cdot q_{B_1} \cdot q_{B_2} \cdot q_C \cdot w_{B_1 B_2} \cdot h_3 \cdot h_B \cdot w_{CR} \\
p_{38} &= q_R \cdot q_{A_1} \cdot q_{A_2} \cdot q_C \cdot w_{A_1 A_2} \cdot h_3 \cdot h_A \cdot w_{CR} \\
p_{39} &= q_R \cdot q_{A_1} \cdot q_{A_2} \cdot q_{B_1} \cdot q_C \cdot w_{A_1 A_2} \cdot w_{B_1 A_2} \cdot h_3 \cdot h_A \cdot w_{CR} \\
p_{40} &= q_R \cdot q_{B_1} \cdot q_{B_2} \cdot q_{A_1} \cdot q_C \cdot w_{B_1 B_2} \cdot w_{A_1 B_2} \cdot h_3 \cdot h_B \cdot w_{CR} \\
p_{41} &= q_R \cdot q_{B_2} \cdot q_C \cdot h_3 \cdot w_{CR} \\
p_{42} &= q_R \cdot q_{A_2} \cdot q_C \cdot h_3 \cdot w_{CR} \\
p_{43} &= q_R \cdot q_{A_1} \cdot q_{B_2} \cdot q_C \cdot w_{A_1 B_2} \cdot h_3 \cdot w_{CR} \\
p_{44} &= q_R \cdot q_{B_1} \cdot q_{A_2} \cdot q_C \cdot w_{B_1 A_2} \cdot h_3 \cdot w_{CR} \\
p_{45} &= q_R \cdot q_C \cdot w_{CR} \cdot h_1 \\
p_{46} &= q_R \cdot q_{B_1} \cdot q_C \cdot w_{CR} \cdot h_2 \\
p_{47} &= q_R \cdot q_{A_1} \cdot q_C \cdot w_{CR} \cdot h_2 \\
p_{48} &= q_R \cdot q_{A_1} \cdot q_{B_1} \cdot q_C \cdot w_{CR} \cdot h_2 .
\end{aligned} \tag{J}
$$

The total partition function for the Or59b cluster is the sum over the partial partition functions:

$$Z_{tot}\left(R, A, B, C, N_{ns}\right) = \sum_{k=1}^{48} p_k \tag{K}$$

where each partial partition function p_k is the product of contributions of all occupied sites and all the interactions implied by the configuration σ_k, compactly written as:

$$p_k = p\left(\sigma_k\right) = \prod_j q_j \prod_n w_{jn} \prod_m h_m \tag{L}$$

with $k = 1, ..., 48$ (the explicit expressions are given above in (I)-(J)), $j, n \in \{A_1,\ A_2,\ B_1,\ B_2,\ C,\ R\}$ (see (H) for the pairs of parameters considered in w_{jn}) and $m \in \{1,\ 2,\ 3,\ A,\ B\}$. Hence the normalized weights for the configuration states σ_k are

$$P(\sigma_k) = \frac{p_k}{Z_{tot}} = \frac{p_k}{\sum_{j=1}^{48} p_j}.$$

This is the quantity shown in Fig. 3 of the paper for a specific sample, and in Figs E-L for the ensembles of parameter samples considered in the analysis.

The probability of RNAp binding to the TATAbox, driven by the entire cluster Or59b, denoted $P^{\text{Or59b}}_{\text{binding}}(\text{R}-\text{TATAbox})$, is the sum of the configurations in which RNAp is bound divided by the partition function, as follows:

$$P^{\text{Or59b}}_{\text{binding}}(\text{R}-\text{TATAbox}) = \frac{\sum_{k=25}^{48} p_k}{\sum_{k=1}^{48} p_k}. \tag{M}$$

Notice that if we divide the numerator by itself and the denominator by the numerator, we get:

$$P^{\text{Or59b}}_{\text{binding}}(\text{R}-\text{TATAbox}) = \frac{1}{1 + \frac{\sum_{k=1}^{24} p_k}{\sum_{k=25}^{48} p_k}}. \tag{N}$$

Eq. (N) is the main equation used in the paper.

1.4 Physical ranges for the model parameters

For our system, neither the precise values of the TFs concentration profiles, nor the strength of the affinity of TFs for specific and non-specific binding sites are available. Coherent biological ranges for the parameter values can only be obtained indirectly.

Unlike *E.coli* cells, whose DNA length is about $5 \cdot 10^6$ bps and contains roughly $\sim 10^3$ RNA polymerases, the *Drosophila* genome contains $120 \cdot 10^6$ accessible bps and a much larger number of RNAp. To give an order of magnitude, in the literature the number of engaged RNAp II complexes in mouse is estimated to be $\sim 6 \cdot 10^4$ [5], and the number of RNAp II (Rpb3) molecules per haploid cell in budding yeast about $3 \cdot 10^4$ copies per cell [14].

In order to determine the parameter q_R, a range from 10^3 RNAp molecules up to $8 \cdot 10^4$ was used. Assuming that the RNAp binding probability is around 1% for an unregulated promoter, the values of q_R we obtain are between 0.002 and 0.03, interval which we impose in the sampling (see Table 2). For several transcription factors, the number of molecules in the nucleus is estimated to range from $2 \cdot 10^4$ to 10^6 in *Drosophila* [2]. It is commonly thought that the amount of RNAp is higher than any of the TFs. For this reason the range for the number of molecules of species A, B and C is set to $(10^3 - 5 \cdot 10^4)$. The values of the dissociation constants being unknown as well, a range between 20 and 10000 nM for each K_j is chosen. This gives us a range for q_{A_1}, q_{A_2}, q_{B_1}, q_{B_2} and q_C of $(0.1 - 2500)$. Recall that $q_A = q_{A_1} \cdot q_{A_2}$ and $q_B = q_{B_1} \cdot q_{B_2}$. A mutated DNA site has a residual binding energy, much lower than q_j. For our model we choose values $10^{-6} \div 10^{-5}$.

Considering the TF-TF interactions, these can be represented by interaction factors w_{jn} taking values within $(10^1 - 10^3)$, according to [12, 1]. In our model, the parameters $w_{A_1A_2}$ and $w_{B_1B_2}$ are set in the range of $(10 - 100)$, and w_{CR} in the range of $(30 - 100)$. The interaction factors $w_{A_1B_2}$ and $w_{B_1A_2}$ represent a negative action from the transcription factor attached to an Homeobox site over a TF bound to Pou site. For the sampling, these parameters take values ranging from $2 \cdot 10^{-4}$ to 10^{-3}. All parameter ranges are reported in Tables 2- 3.

The chromatin-related parameters need to be tuned correctly to achieve the expected transcriptional output, resulting in different values depending on whether the normal chromatin state, or the heterozygous mutant version of su(var)3-9, or the homozygous version is considered (see below for more details). For the parameter h_1, we choose values that decrease passing from closed chromatin experiments (column C in Table 1) to heterozygous mutant su(var)3-9 (column H in Table 1) and to homozygous mutant su(var)3-9 (column N in Table 1). The opposite order (increasing) is instead occurring for the parameters h_2 and h_3. Numerical values for the epigenetic parameters are reported in Table 4. In Table 1, when passing from C to H, there is a stark contrast between the behavior of E8 and E12. The gain in E8 weakens, while in E12 it stays strong. In both E8 and E12 one Homeobox site is mutated, hence the most plausible explanation for this difference is that the remaining cooperative interaction (Pdm3 bound to both Pdm3Hox and Pou for E8, and Acj6 bound to both Acj6Hox and Pou for E12) assumes values that diverge when chromatin is "opened". Notice that the trend is confirmed (and amplified) in our validation experiments with homozygous mutation in su(var)3-9 (column N in Table 1). The numerical solution we adopt to capture this behavior consists in increasing h_A and decreasing h_B as we pass from C to H and to N. Numerical values are given in Table 4.

Given the concentration of the species, the cooperative and non-cooperative factors and the binding strengths for all the DNA sites, from Eq. (L) the vector of nonnormalized partial probabilities p_k, $k = 1, \dots, 48$, can be computed. Consequently, from Eq. (N), also the overall probability $P^{\text{Or59b}}_{\text{binding}}(\text{R} - \text{TATAbox})$ of the RNAp binding to the promoter of interest can be computed. Changing the values of the parameters of the model, so do the p_k for the various sites mutations represented in Table 1 and hence so does $P^{\text{Or59b}}_{\text{binding}}(\text{R} - \text{TATAbox})$, which allows to look at empirical distributions, as we do e.g. in Fig. 2 of the paper.

1.5 Random sampling of the parameter space

The procedure consisted in producing a large quantity of batches of samples, each of size $\sim 10^5$, randomly choosing the binding affinities q_j and the interaction parameters w_{jn} within the values given in Tables 2-3, and progressively refining the epigenetic parameters. A first screening was aimed at finding sets $\{q_j,\, w_{jn},\, h^C_m\}$ (h^C_m = epigenetic parameters valid for the column C) so as to satisfy the following set distance threshold

$$\Phi^C(q_j,\, w_{jn},\, h^C_m) = \sum_{j=1,\dots,16} d\left(P^{\text{Or59b}}_{\text{binding}}\left(\text{R} - \text{TATAbox}, \theta^C_j\right), [\ell^C_j,\; u^C_j]\right) < \tau = 0.05 \tag{O}$$

where $d(x, \mathcal{Y})$ = set distance between x and the set $\mathcal{Y}$ (see Methods of the main paper) $P^{\text{Or59b}}_{\text{binding}}\left(\text{R} - \text{TATAbox}, \theta^C_j\right)$ is the RNAp probability in correspondence of the experiments in the column C (θ^C_j = indicator variable of the j-th row of the truth table in the C column) and $[\ell^C_j,\; u^C_j]$ is the empirical interval obtained from the j-th experiment in the column C, see Table 1. See Fig. M(A) for how an histogram of the distance Φ^C in one such batch (before any refinement) looks like. Subsequently, we tried to combine (O) with an analogous set distance constraint

on the H column. This was however impossible without altering the epigenetic parameters. Therefore we opted for an epigenetic retuning, i.e., allowed changes in the epigenetic parameters. Denoting h_m^H the new set, we produced novel random samples aiming at fulfilling simultaneously (O) and the following:

$$\Phi^H(q_j,\, w_{jn},\, h_m^H) = \sum_{j=1,\ldots,16} d\left(P^{\text{Or59b}}_{\text{binding}}\left(\text{R} - \text{TATAbox}, \theta_j^H\right), [\ell_j^H,\; u_j^H]\right) < \tau = 0.05 \tag{P}$$

This fitting phase relied exclusively on the C and H columns of Table 1, while the column N was kept for a further refinement of the results and for validation. When these samples are used to predict expression for the columns C and H of the truth table (Table 1), only a small fraction of samples (around 0.5% of a batch size) matches exactly the constraints of the entire table (blue vertical line in Fig M(B)). With a proper tuning of the epigenetic parameters, most samples fulfilling the constraints of columns C and H also satisfy those in the column N. The rationale behind the allowed changes in the values of the epigenetic parameters in passing from C and H to N is explained in the previous section.

2 List of experiments

A set of experiments involving mutant species and sites, knockdown or overexpression of the TF, and trimethylation of the chromatin, were done [11] in order to investigate how the Or59b cluster regulates expression and how cooperative TF function generates robust class-specific OR expression. The experiments are described in detail in [11] and briefly summarized below, together with some new experiments produced explicitly for this work.

2.1 Experiments concerning motifs mutation and chromatin trimethylation by heterozygous mutant su(var)3-9.

In order to detect the regulatory function of each TF, mutations in the different binding sites of the Or59b cluster were made for the constructs. These were tested in various combinations, and in the epigenetic state induced by heterozygous mutant su(var)3-9, as summarized in Table A, columns C and H.

Unlike [11], where the expression level was classified as "all or nothing" depending on whether it was above or below a certain threshold, a more fine-graded classification is needed in this work. Or59b cluster driven expression is quantified manually counting the number of GFP-expressing neurons in the region DM4 in the antenna and antennal lobe, where the axons of Or59b are projected, through a GFP reporter fused to the TATAbox driven by the Or59b cluster. Ectopic expression is disregarded.

The experiments performed in [11] are recapitulated in Table A, yellow and green cells. The total number of flies analyzed is reported, together with the number of flies having a nonzero GFP expression in DM4. For each fly, the number of OSNs is then reported (see also Fig. 1(B)). Overall we obtained OSN counts between 0 and 150, hence normalized values ("probabilities of Or59b expression") were obtained dividing all counts by 150. In each experiment the number of flies varied between 15 and 38. Given the broad difference in OSN countings across "replicates" (i.e., on different flies from the same experiment), we prefer to avoid quantifying GFP expression in terms of mean + std. deviation (or median and quartile) in the countings of OSNs. We opted instead for defining an interval $[\ell, u]$ in each experiment, corresponding to the (normalized) min and max count of OSNs. In this way we avoid biasing the data when multiple phenotypes appear, essentially corresponding to subsets of flies showing no expression (see e.g. EH16 in Table A). Furthermore, a description of the measured observable as an interval is more suitable for our modeling approach (which produces probability distribution for the model output).

The cases for which an experiment was performed are listed in Table A. Cells in grey correspond to cases in which we know already the outcome: mutation of the Ebox caused total loss of the expression, thus all "odd" rows of Table A can be safely marked as "loss". To obtain putative experimental values also for double-site mutations (not involving Fer1, i.e., for rows E4, E6 and E10 of Table A), we used other experiments combining single site mutation and knockdown/knockout of a TF. In particular, a very low level of Acj6, obtained through Acj6^6 males was used in conjunction with Pdm3Hox and Pou mutations (obtaining respectively the rows E4 and E6 in Table A), and a very low level of Pdm3, obtained through RNAi, was used in conjunction with Pou mutation (obtaining the row E10 of Table A). These experiments are clearly only a proxy for a true value of double mutant, meant to enrich Table A.

Sometimes in these experiments the whole conformation of the promoter and the chemical balance of the cell varies in a way that the TFs change their role according to different equilibrium of co-regulators and different splice forms of the same TF. For instance, when the heterozygous mutant su(var)3-9 was introduced without mutant motifs, 3 different phenotypes emerged, with some flies showing loss, others gain, and other gain plus ectopic expression, see Table B. This case corresponds to EH16 in Tables 1 and A.

Even after the indirect experiments are taken into account, the truth table has still several combinations for which an expression value is unknown. These are shown in white in Tables 1 and A.

Code	Acj6Hox	Pdm3Hox	Pou	Ebox	Expression driven by the Or59b cluster<br>**C**<br>normal chromatin state<br>(Data from [11])	 **H**<br>heterozygous<br>su(var)3-9 mutant<br>(Data from [11])	 **N**<br>homozygous su(var)3-9 mutant
E1	0	0	0	0	loss	loss	loss
E2	0	0	0	1	N. of expressing/total flies: 0/25<br>...<br>N. of OSNs: 0 0 0 0 0 0 0 0 0 0 0 0 0 0 0 0 0 0 0 0 0 0 0 0 0		
E3	0	0	1	0	loss	loss	loss
E4	0	0	1	1	N. of expressing/total flies: 14/14<br>...<br>N. of OSNs: 35 37 38 38 38 39 41 42 42 42 46 46 49 50<br>...<br>Indirect experiment (mutant Pdm3Hox + Acj6 Males)		N. of expressing/total flies: 18/18<br>...<br>N. of OSNs: 44 45 45 45 46 46 49 50 52 53 54 56 57 58 59 60 63 65<br>...<br>Indirect experiment (mutant Pdm3Hox + Acj6 Males).
E5	0	1	0	0	loss loss	loss	loss
E6	0	1	0	1	N. of expressing/total flies: 3/20<br>...<br>N. of OSNs: 0 0 0 0 0 0 0 0 0 0 0 0 0 0 0 0 0 15 17 20<br>...<br>Indirect experiment (mutated Pou + Acj6 Males)		N. of expressing/total flies: 15/20<br>...<br>N. of OSNs: 0 0 0 0 0 40 41 42 45 46 46 47 49 50 51 52 54 55 55 55<br>...<br>Indirect experiment (mutated Pou + Acj6 Males).
E7	0	1	1	0	loss	loss	loss
E8	0	1	1	1	N. of expressing/total flies: 35/35<br>...<br>N. of OSNs: 60 60 61 62 62 63 63 64 64 65 65 66 66 66 66 66 66 66 67 67 67 68 68 68 69 69 69 69 69 69 69 69 70 70 70	N. of expressing/total flies: 15/15<br>...<br>N. of OSNs: 10 10 11 14 14 15 15 15 16 16 17 18 18 19 20	N. of expressing/total flies: 12/15<br>...<br>N. of OSNs: 0 0 0 3 4 5 6 6 7 7 7 8 8 10 10
E9	1	0	0	0	loss	loss	loss
E10	1	0	0	1	N. of expressing/total flies: 8/8<br>...<br>N. of OSNs: 25 27 27 27 28 30 30 30<br>...<br>Indirect experiment (mutated Pou + Pdm3-IR)		
E11	1	0	1	0	loss	loss	loss
E12	1	0	1	1	N. of expressing/total flies: 23/23<br>...<br>N. of OSNs: 104 104 108 109 112 112 114 116 117 125 125 126 126 127 130 130 132 132 133 141 144 145 149	N. of expressing/total flies: 25/25<br>...<br>N. of OSNs: 100 101 101 107 108 111 111 112 112 112 118 121 123 125 126 127 129 131 131 133 135 141 145 148 150	N. of expressing/total flies: 23/23<br>...<br>N. of OSNs: 100 103 103 108 112 113 115 117 117 118 121 122 125 125 128 128 131 136 136 140 144 147 150
E13	1	1	0	0	loss	loss	loss
E14	1	1	0	1	N. of expressing/total flies: 3/25<br>...<br>N. of OSNs: 0 0 0 0 0 0 0 0 0 0 0 0 0 0 0 0 0 0 0 0 0 0 2 4 5	N. of expressing/total flies: 10/25<br>...<br>N. of OSNs: 0 0 0 0 0 0 0 0 0 0 0 0 0 0 0 5 7 10 10 11 12 14 15 18 25	N. of expressing/total flies: 20/20<br>...<br>N. of OSNs: 15 15 15 16 17 17 17 17 18 18 19 22 23 24 26 26 27 27 27 30
E15	1	1	1	0	N. of expressing/total flies: 0/20<br>...<br>N. of OSNs: 0 0 0 0 0 0 0 0 0 0 0 0 0 0 0 0 0 0 0 0	N. of expressing/total flies: 0/21<br>...<br>N. of OSNs: 0 0 0 0 0 0 0 0 0 0 0 0 0 0 0 0 0 0 0 0 0	N. of expressing/total flies: 0/15<br>...<br>N. of OSNs: 0 0 0 0 0 0 0 0 0 0 0 0 0 0 0
E16	1	1	1	1	N. of expressing/total flies: 38/38<br>...<br>N. of OSNs: 20 21 22 23 24 24 26 27 28 28 29 29 31 32 32 33 35 36 36 38 39 39 40 40 40 41 41 43 43 43 43 45 46 47 47 48 49 50	N. of expressing/total flies: 20/28<br>...<br>N. of OSNs: 0 0 0 0 0 0 0 0 20 21 25 25 25 28 29 29 30 30 30 33 34 35 35 36 36 38 39 39 40 40 40 60 68 73	N. of expressing/total flies: 25/25<br>...<br>N. of OSNs: 60 60 60 60 60 61 62 62 62 63 63 63 64 64 64 65 67 68 68 68 69 69 69 69 69

Table A: **Truth table of the expression phenotypes for the Or59b cluster experiments.** The number of neurons for which the Or59b cluster drives expression in the glomerulus DM4 was manually counted on whole brain stainings, through a GFP reporter fused with to the TATA box. For each mutant the total number of flies analyzed and the total number of expressing flies is reported. It is followed by the count of the number of OSNs in each fly. The color code is the same as in Table 1 of the paper. Indirect experiments (in green and blue) are indicated explicitly. Examples of single fly stainings are shown in Fig. 5(A-C) and Fig. 1(C) for E8, E12, E14 and EC16.

normal chromatin	heterozygous mutant su(var)3-9
1-OSN restricted-class expression	23% Loss<br>67% 1-OSN restricted-class expression<br>10% Ectopic expression

Table B: Case E16, C and H: percentages of flies population showing different expression patterns when heterozygous mutant su(var)3-9 is introduced. Data from [11].

2.2 Experiments concerning motifs mutation and chromatin trimethylation by homozygous mutant su(var)3-9.

Novel experiments with/without binding site mutations were performed in homozygous mutant su(var)3-9 background (see Materials section in the paper). These are listed in the column N of Tables 1 and A. Mutations of Acj6Hox, Pdm3Hox and Pou were tested in this background (rows E8, E12, and E14 of Tables 1 and A). The mutants Pdm3Hox and Pou were also tested with Acj6 knockout ($Acj6^6$ males). As done in the C column, these last two experiments are treated here as double site mutants: rows E4 and E6 in Tables 1 and A. Quantification of expression in all new experiments in column N was done as in columns C and H.

References

[1] G. K. Ackers, A. D. Johnson, and M. A. Shea. Quantitative model for gene regulation by lambda phage repressor. *Proc Natl Acad Sci USA*, 79(4):1129–33, 1982.

[2] Mark D. Biggin. Animal transcription networks as highly connected, quantitative continua, developmental cell . *Cell*, 21, Issue 4:611–626, 2011.

[3] Lacramioara Bintu, Nicolas E Buchler, Hernan G Garcia, Ulrich Gerland, Terence Hwa, Jan Kondev, Thomas Kuhlman, and Rob Phillips. Transcriptional regulation by the numbers: applications. *Current Opinion in Genetics & Development*, 15:125–35, 2005.

[4] Lacramioara Bintu, Nicolas E Buchler, Hernan G Garcia, Ulrich Gerland, Terence Hwa, Jan Kondev, Thomas Kuhlman, and Rob Phillips. Transcriptional regulation by the numbers: models. *Curr Opin in Genet & Dev*, 15:116–24, 2005.

[5] Jackson DA, Pombo A, and Iborra F. The balance sheet for transcription: an analysis of nuclear rna metabolism in mammalian cells. *FASEB Journal*, 14(2):242–54., 2000.

[6] Ken A. Dill and Sarina Bromberg. *Molecular driving forces: statistical thermodynamics in chemistry and biology.* New York, 2003.

[7] Hernan G. Garcia, Jan Kondev, Nigel Orme, Julie A. Theriot, and Rob Phillips. A first exposure to statistical mechanics for life. Technical report, arXiv:0708.1899, 2007.

[8] Hernan G. Garcia, Alvaro Sanchez, Thomas Kuhlman, Jan Kondev, and Rob Phillips. Transcription by the numbers redux: experiments and calculations that surprise. *Cell Biology*, 20:723–33, 2010.

[9] U. Gerland, J.D. Moroz, and T.Hwa. Physical constraints and functional characteristics of transcription factor-DNA interaction. *Proc Natl Acad Sci USA*, 99(19):12015–20, 2002.

[10] Terrell L. Hill. *Cooperativity theory in biochemistry: steady-state and equilibrium systems.* New York, 1985.

[11] Shadi Jafari and Mattias Alenius. Cis-regulatory mechanisms for robust olfactory sensory neuron class-restricted odorant receptor gene expression in Drosophila. *PLOS Genetics*, 11(3):e1005051, 2015.

[12] Buchler NE, Gerland U, and Hwa T. On schemes of combinatorial transcription logic. *Proc Natl Acad Sci U S A*, 100(9):5136–41, 2003.

[13] Kim Sneppen and Giovani Zocchi. *Physics in Molecular Biology.* Cambridge University Press, 2005.

[14] Borggrefe T, Davis R, Bareket-Samish A, and Kornberg RD. Quantitation of the RNA polymerase II transcription machinery in yeast. *J Biol Chem*, 276(50):47150–3 Table, 2001.

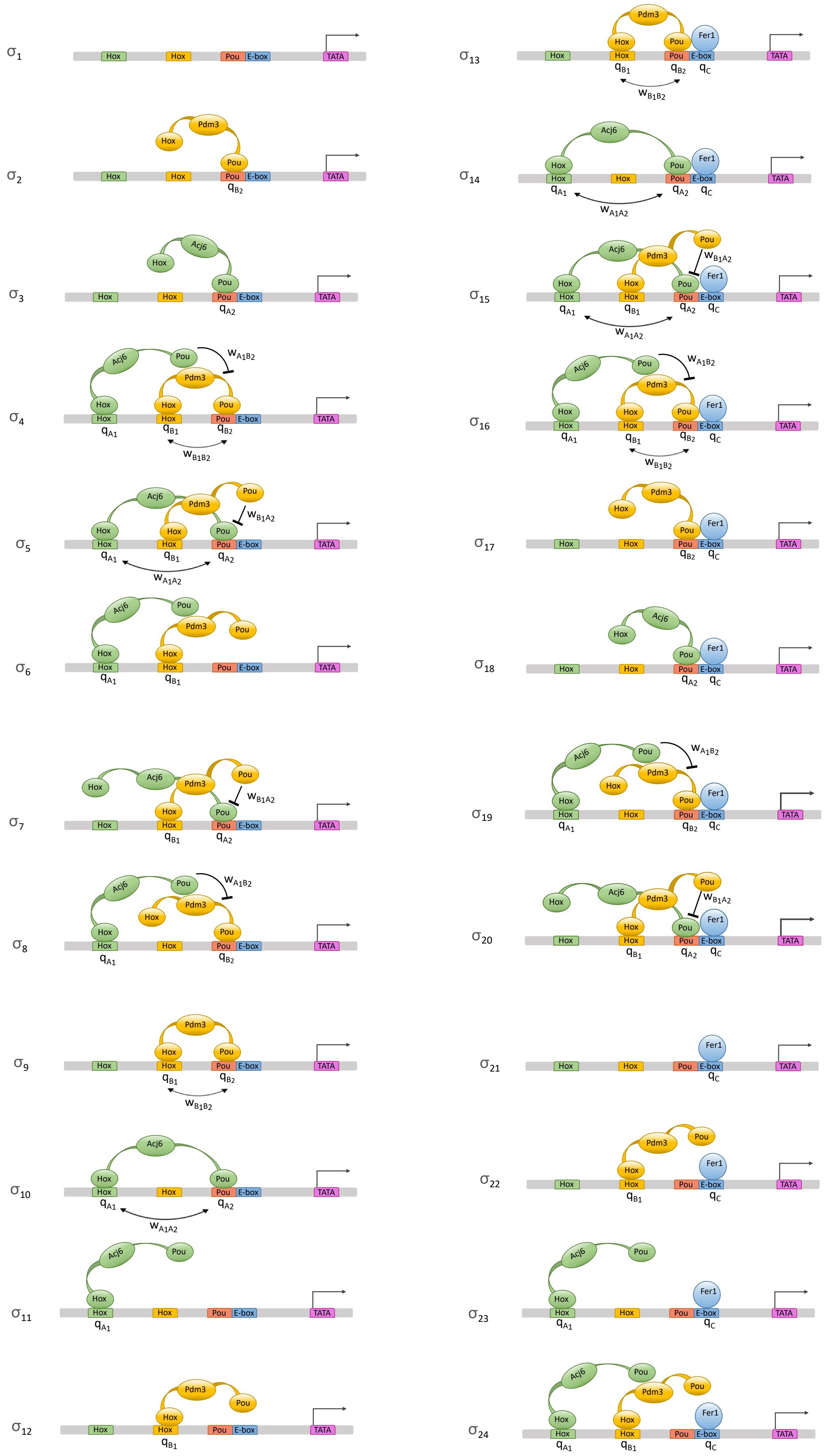

Figure A: List of configuration states σ_k for the Or59b cluster (part 1/2).

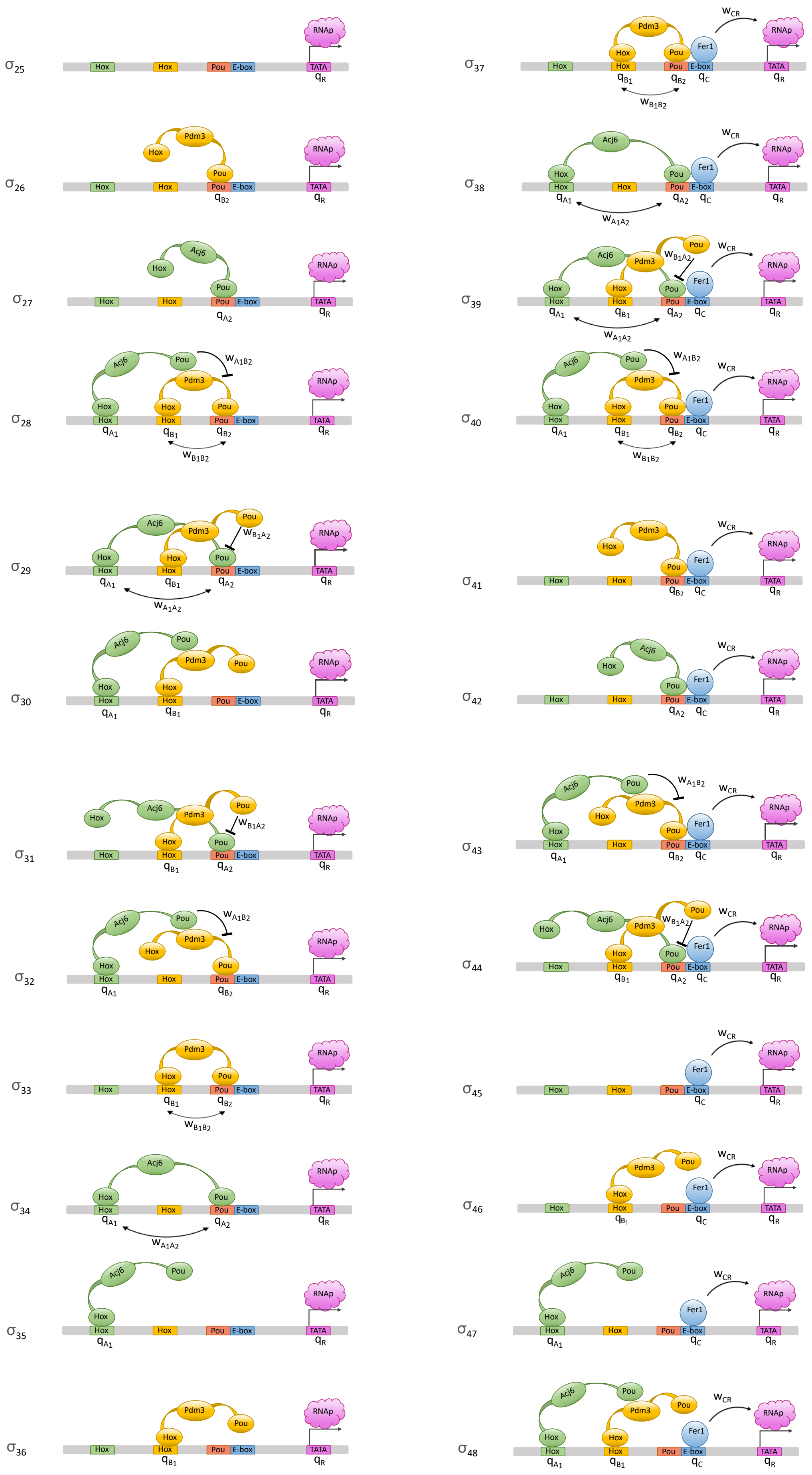

Figure B: List of configuration states σ_k for the Or59b cluster (part 2/2).

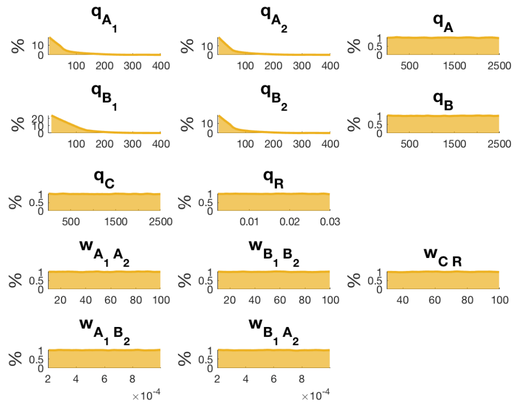

(i)

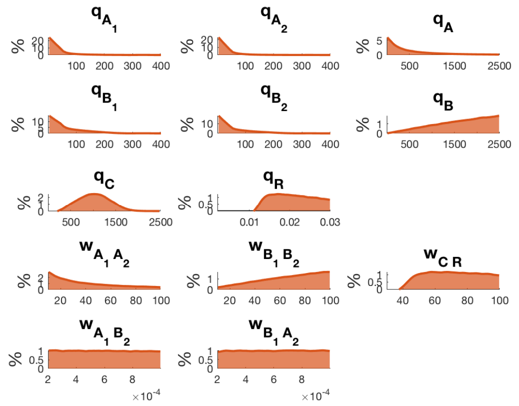

(ii)

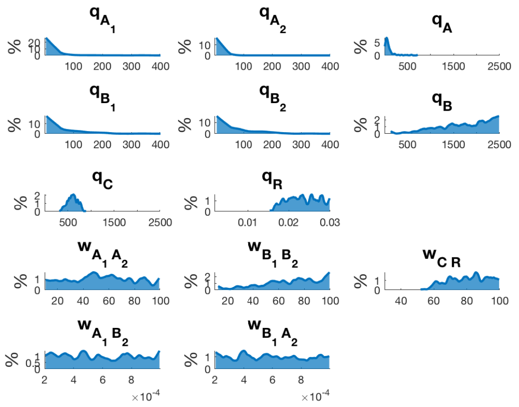

(iii)

Figure C: Histogram of the sample values of the parameters used in the model. (i): Entire sample population. In the first 3 rows are binding affinities, followed in the fourth row by the cooperative interactions and in the fifth row by the competitive interactions. All distributions are uniform, except for those parameters whose product must be a uniform distribution (i.e., the pairs $\{q_{A_1},\ q_{A_2}\}$ and $\{q_{B_1},\ q_{B_2}\}$). (ii): Subset of samples satisfying simultaneously (O) and (P). (iii): Sample subset giving a correct gain/loss prediction for the entire truth table (Table 1), in the 3 columns C, H and N. Passing from (A) to (B) the main qualitative changes are the asymmetry between the binding affinities q_A and q_B, and between the cooperativity coefficients $w_{A_1A_2}$ and $w_{B_1B_2}$. Also q_C, q_R and w_{CR} are no longer uniform. Passing from (B) to (C) the difference between q_A and q_B is emphasized, while that between $w_{A_1A_2}$ and $w_{B_1B_2}$ is reduced. The "supports" of q_C, q_R and w_{CR} all shrink (most notably for q_C). No specific effect is instead observed in the competitive interactions $w_{A_1B_2}$ and $w_{B_1A_2}$. See Fig. 4 for a more detailed analysis.

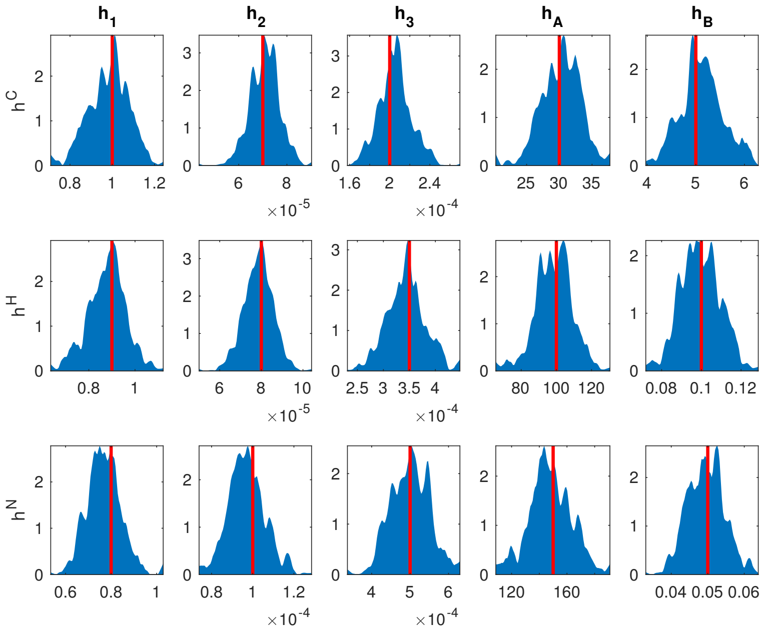

Figure D: Histograms of epigenetic parameters. For the around 10^3 samples matching all interval constraints of Table 1 on all 3 columns C, H, and N, the epigenetic parameters are normally distributed around the nominal values provided in Table 4 (red vertical lines).

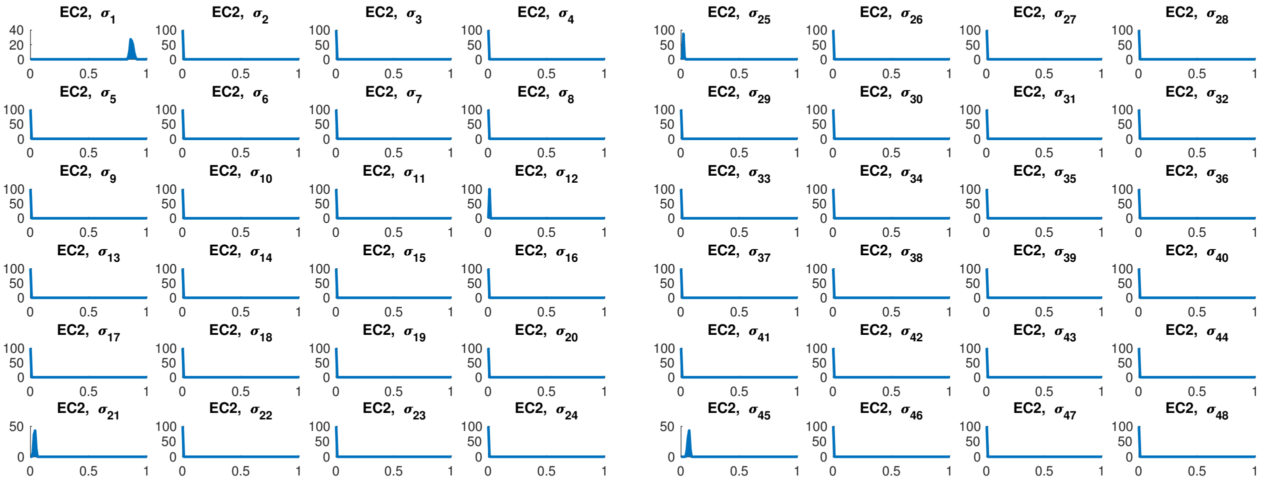

(i)

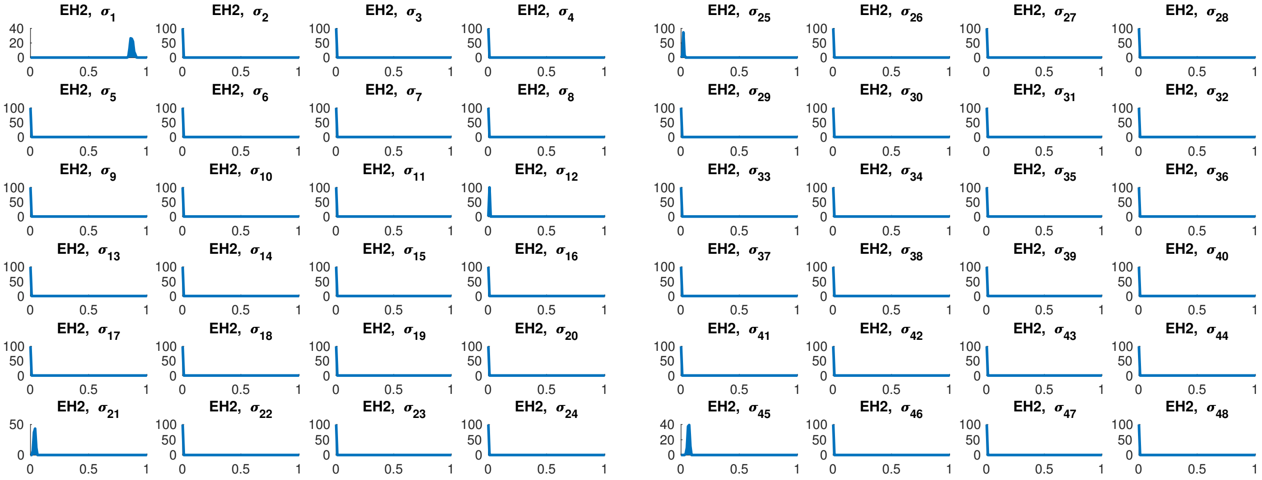

(ii)

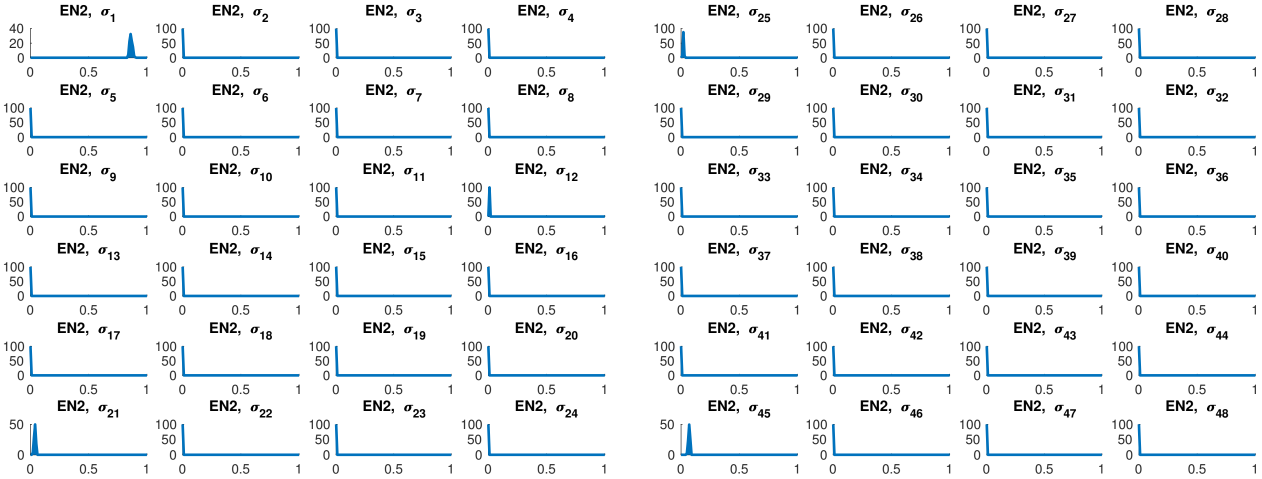

(iii)

Figure E: Probabilities $P(\sigma_k)$ for the triple mutation of Acj6Hox, Pdm3Hox and Pou (i.e., row E2 of Table 1) in open and closed chromatin. (i): probability distribution of the 48 σ_k states in normal chromatin (EC2 of Table 1) for the parameter sets correctly predicting gain/loss for the entire truth table. In the left panel RNAp is not bound to the TATAbox, in the right panel it is. (ii): probabilities of the same configurations for heterozygous su(var)3-9 mutant (i.e., EH2 in Table 1). (iii): probabilities of the same configurations for homozygous su(var)3-9 mutant (i.e., EN2 in Table 1).

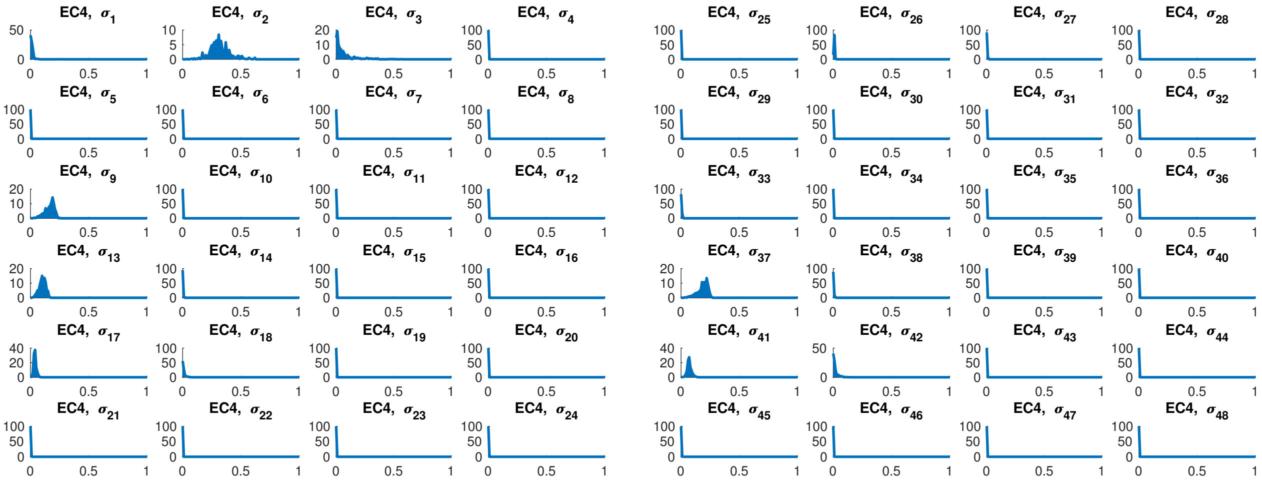

(i)

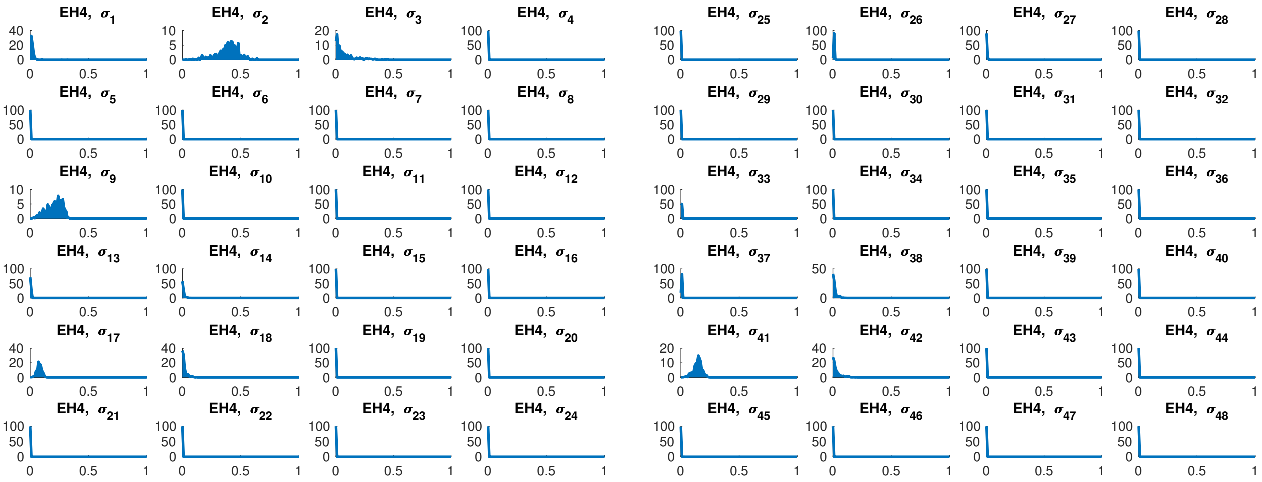

(ii)

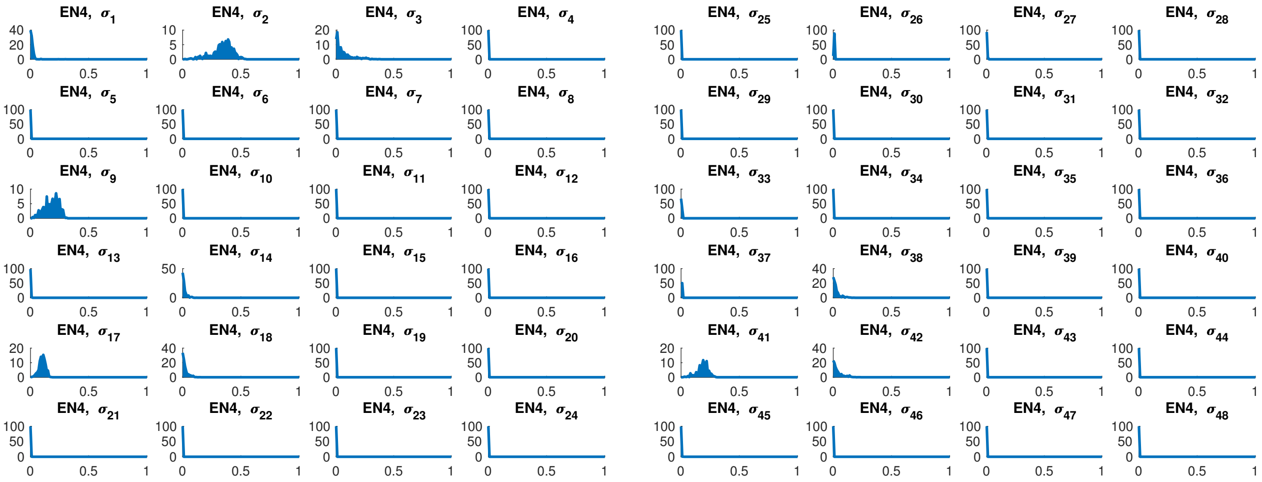

(iii)

Figure F: Probabilities $P(\sigma_k)$ for the double mutation of Acj6Hox and Pdm3Hox (i.e., row E4 of Table 1) in open and closed chromatin. (i): probability distribution of the 48 σ_k states in normal chromatin (EC4 of Table 1) for the parameter sets correctly predicting gain/loss for the entire truth table. In the left panel RNAp is not bound to the TATAbox, in the right panel it is. (ii): probabilities of the same configurations for heterozygous su(var)3-9 mutant (i.e., EH4 in Table 1). (iii): probabilities of the same configurations for homozygous su(var)3-9 mutant (i.e., EN4 in Table 1).

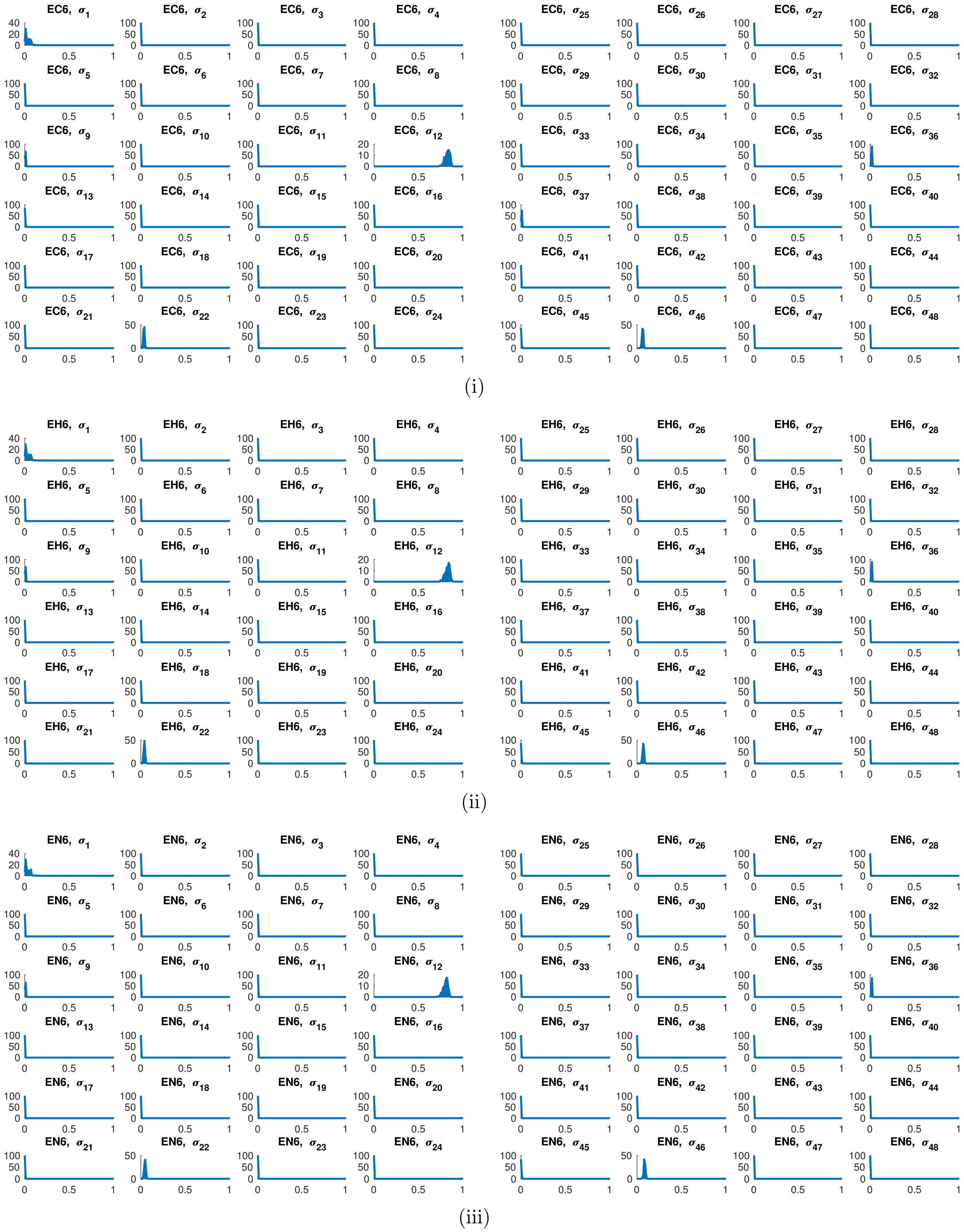

Figure G: Probabilities $P(\sigma_k)$ for the double mutation of Acj6Hox and Pou (i.e., row E6 of Table 1) in open and closed chromatin. (i): probability distribution of the 48 σ_k states in normal chromatin (EC6 of Table 1) for the parameter sets correctly predicting gain/loss for the entire truth table. In the left panel RNAp is not bound to the TATAbox, in the right panel it is. (ii): probabilities of the same configurations for heterozygous su(var)3-9 mutant (i.e., EH6 in Table 1). (iii): probabilities of the same configurations for homozygous su(var)3-9 mutant (i.e., EN6 in Table 1).

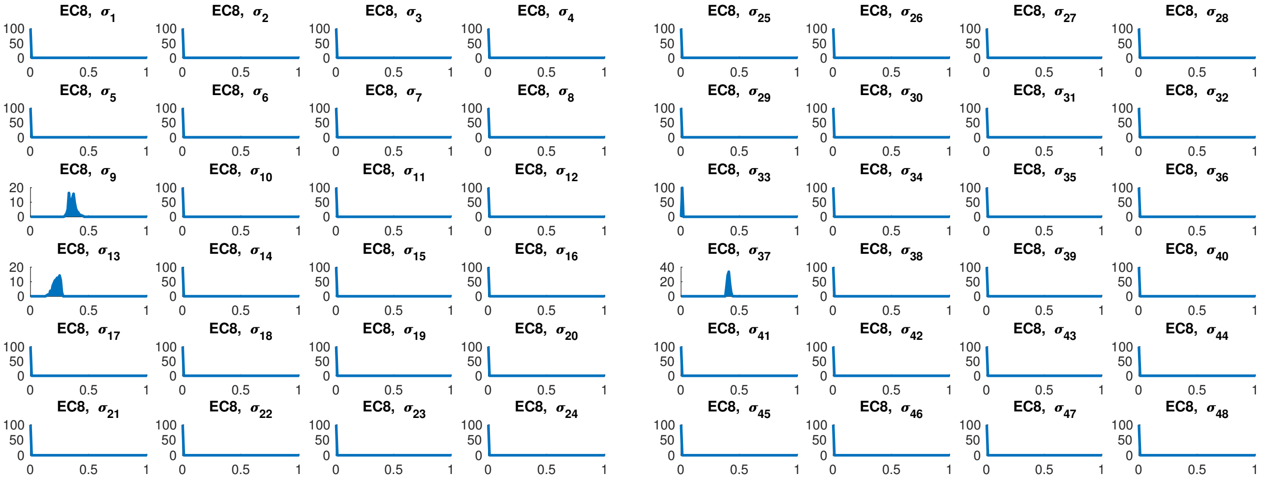

(i)

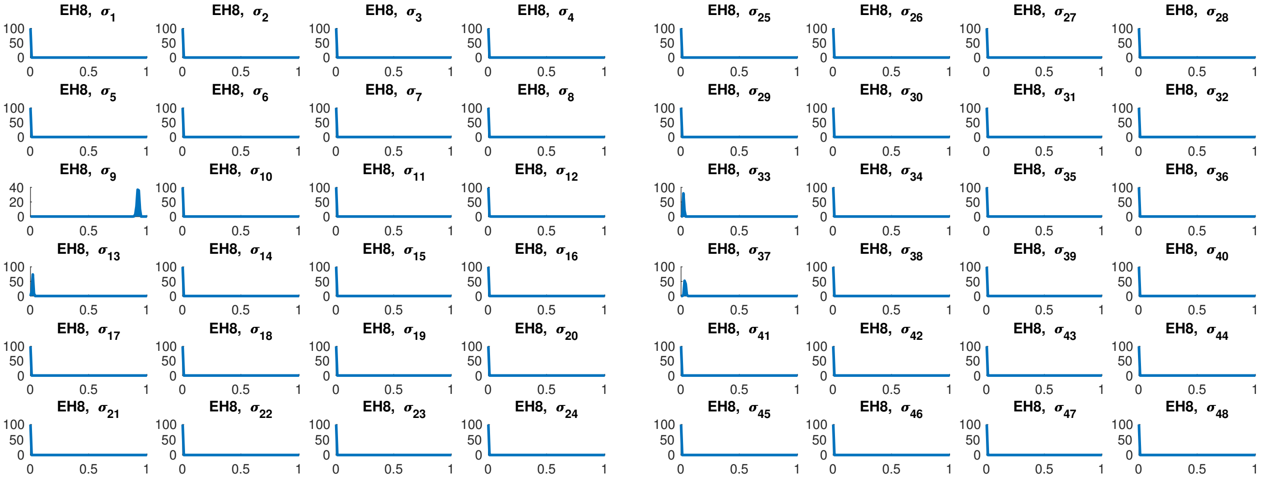

(ii)

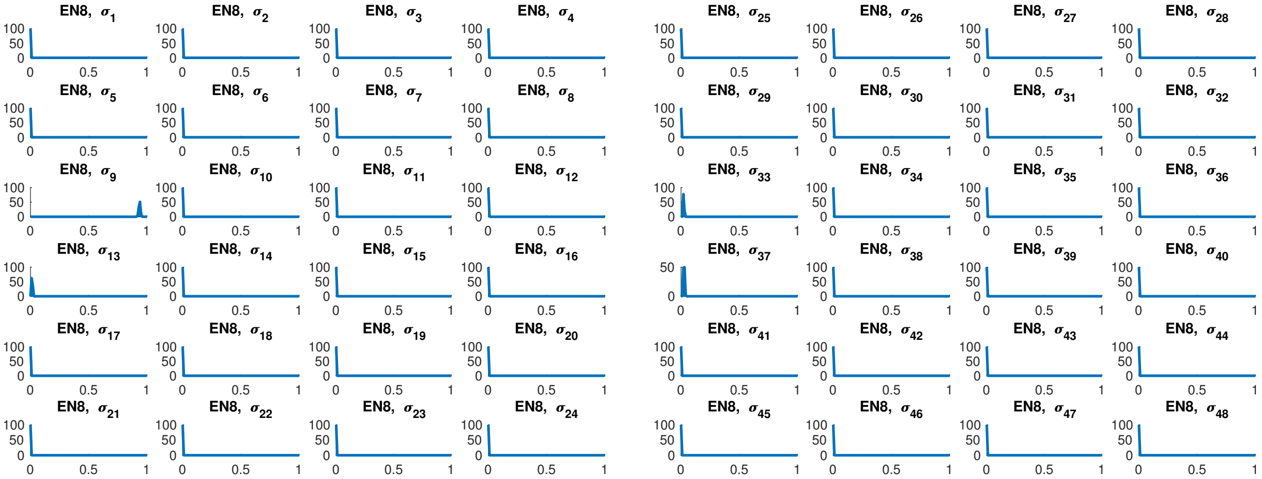

(iii)

Figure H: Probabilities $P(\sigma_k)$ for the Acj6Hox mutation (i.e., row E8 of Table 1) in open and closed chromatin. (i): probability distribution of the 48 σ_k states in normal chromatin (EC8 of Table 1) for the parameter sets correctly predicting gain/loss for the entire truth table. In the left panel RNAp is not bound to the TATAbox, in the right panel it is. (ii): probabilities of the same configurations for heterozygous su(var)3-9 mutant (i.e., EH8 in Table 1). (iii): probabilities of the same configurations for homozygous su(var)3-9 mutant (i.e., EN8 in Table 1).

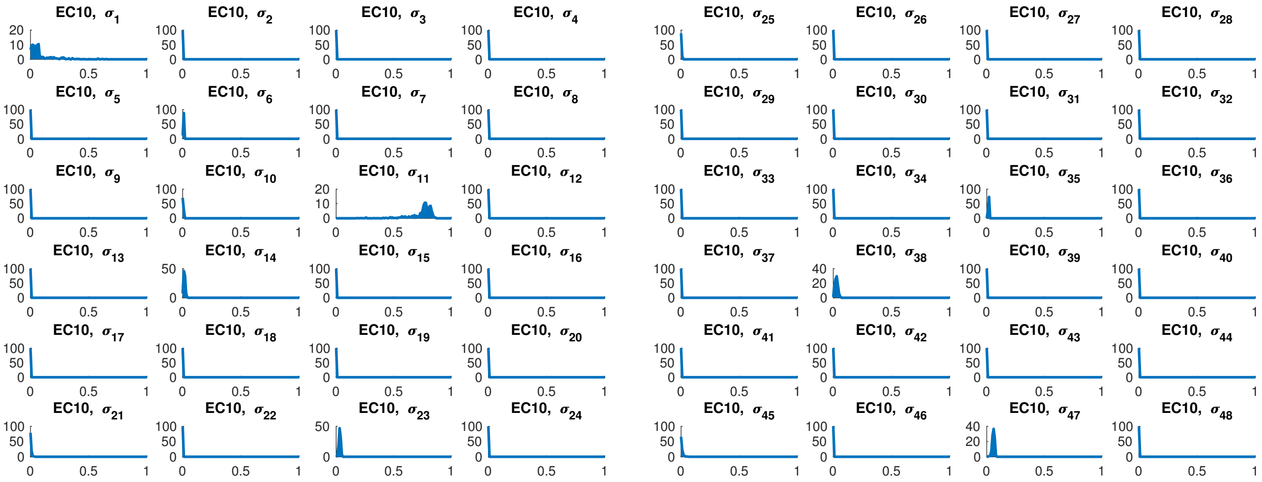

(i)

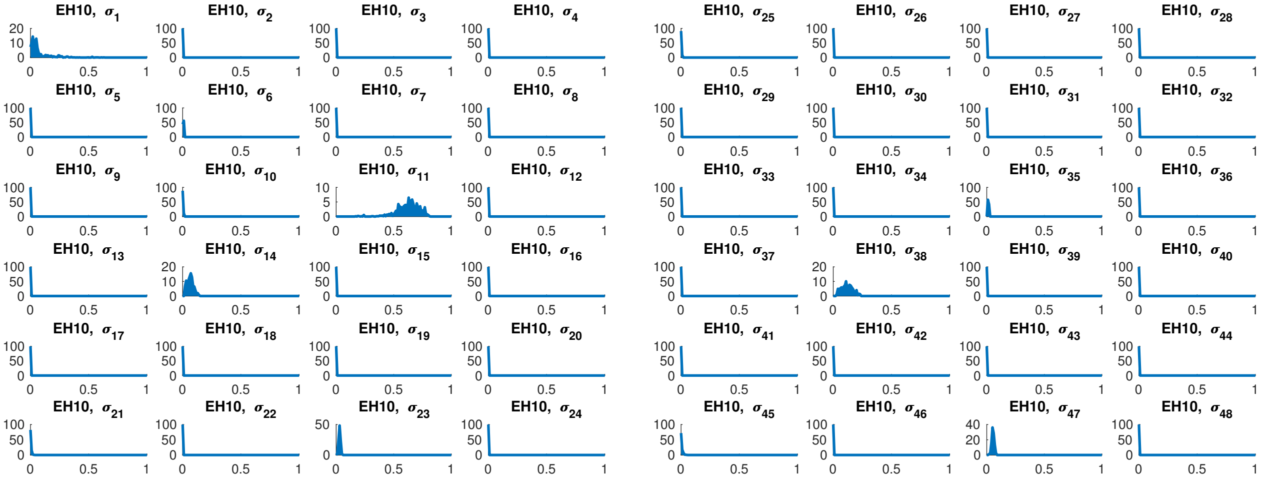

(ii)

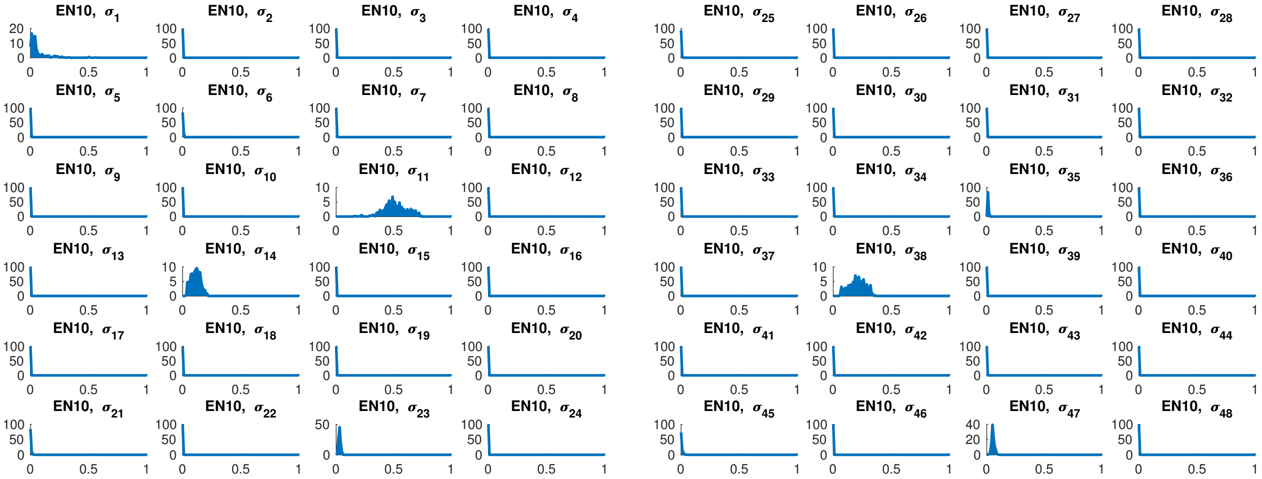

(iii)

Figure I: Probabilities $P(\sigma_k)$ for the double mutation of Pdm3Hox and Pou (i.e., row E10 of Table 1) in open and closed chromatin. (i): probability distribution of the 48 σ_k states in normal chromatin (EC10 of Table 1) for the parameter sets correctly predicting gain/loss for the entire truth table. In the left panel RNAp is not bound to the TATAbox, in the right panel it is. (ii): probabilities of the same configurations for heterozygous su(var)3-9 mutant (i.e., EH10 in Table 1). (iii): probabilities of the same configurations for homozygous su(var)3-9 mutant (i.e., EN10 in Table 1).

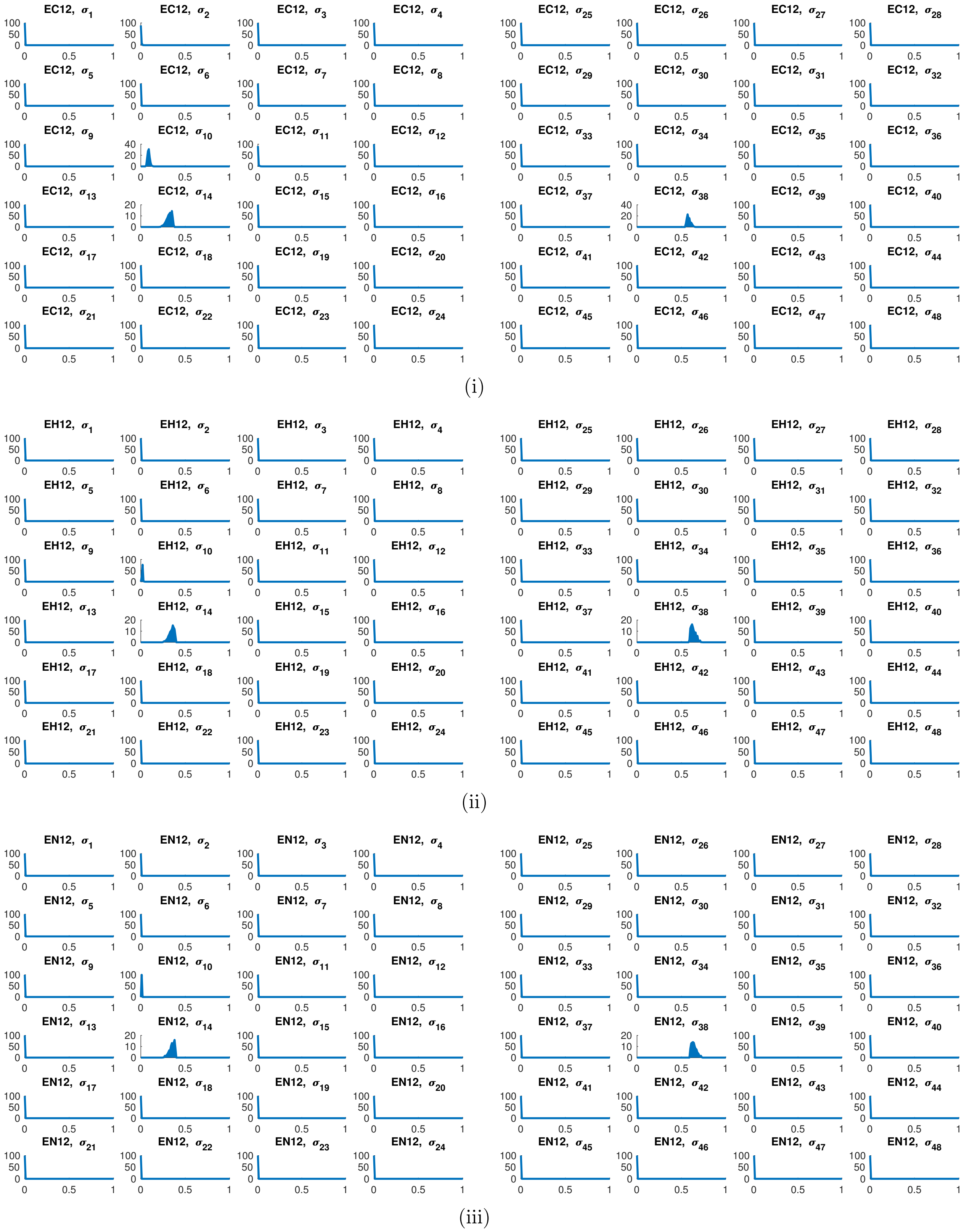

Figure J: Probabilities $P(\sigma_k)$ for the Pdm3Hox mutation (i.e., row E12 of Table 1) in open and closed chromatin. (i): probability distribution of the 48 σ_k states in normal chromatin (EC12 of Table 1) for the parameter sets correctly predicting gain/loss for the entire truth table. In the left panel RNAp is not bound to the TATAbox, in the right panel it is. (ii): probabilities of the same configurations for heterozygous su(var)3-9 mutant (i.e., EH12 in Table 1). (iii): probabilities of the same configurations for homozygous su(var)3-9 mutant (i.e., EN12 in Table 1).

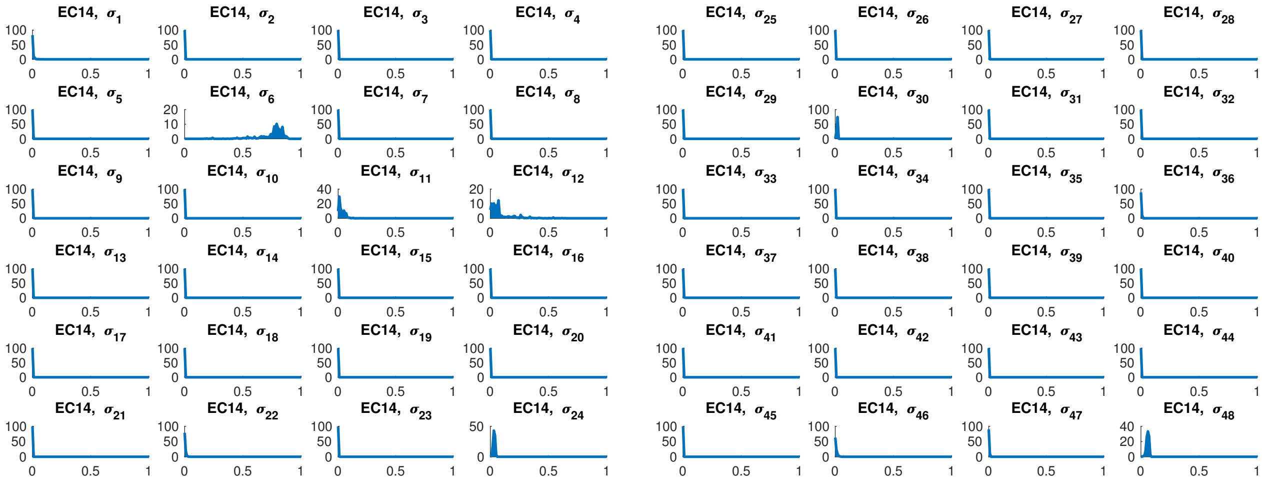

(i)

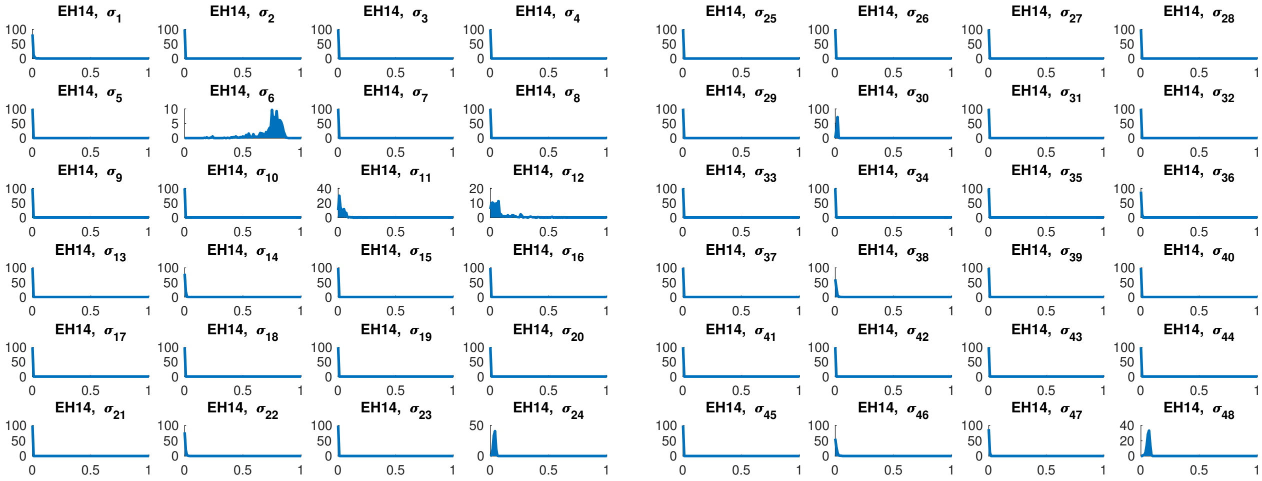

(ii)

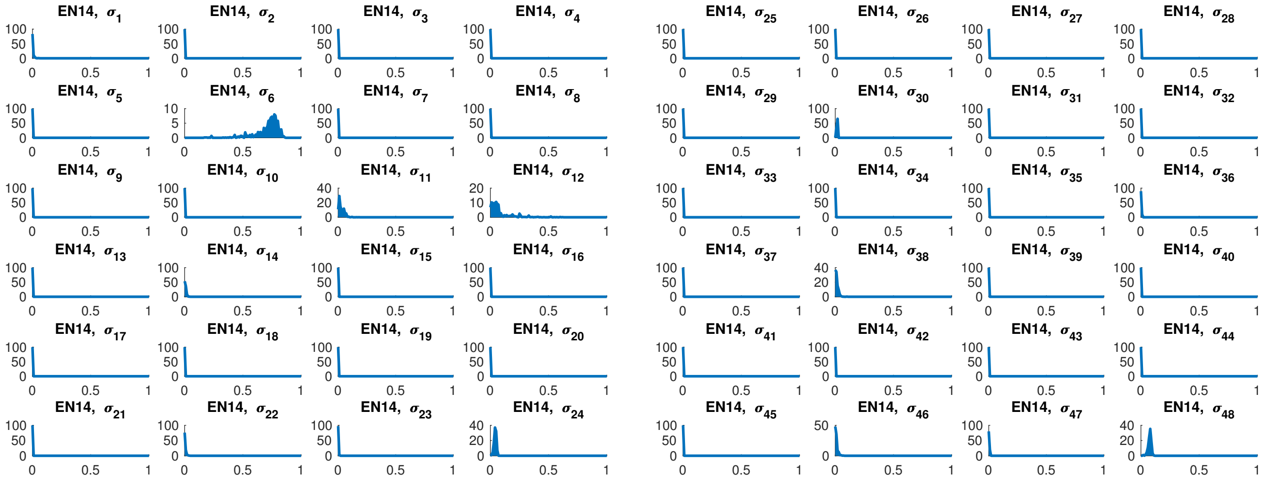

(iii)

Figure K: Probabilities $P(\sigma_k)$ for the Pou mutation (i.e., row E14 of Table 1) in open and closed chromatin. (i): probability distribution of the 48 σ_k states in normal chromatin (EC14 of Table 1) for the parameter sets correctly predicting gain/loss for the entire truth table. In the left panel RNAp is not bound to the TATAbox, in the right panel it is. (ii): probabilities of the same configurations for heterozygous su(var)3-9 mutant (i.e., EH14 in Table 1). (iii): probabilities of the same configurations for homozygous su(var)3-9 mutant (i.e., EN14 in Table 1).

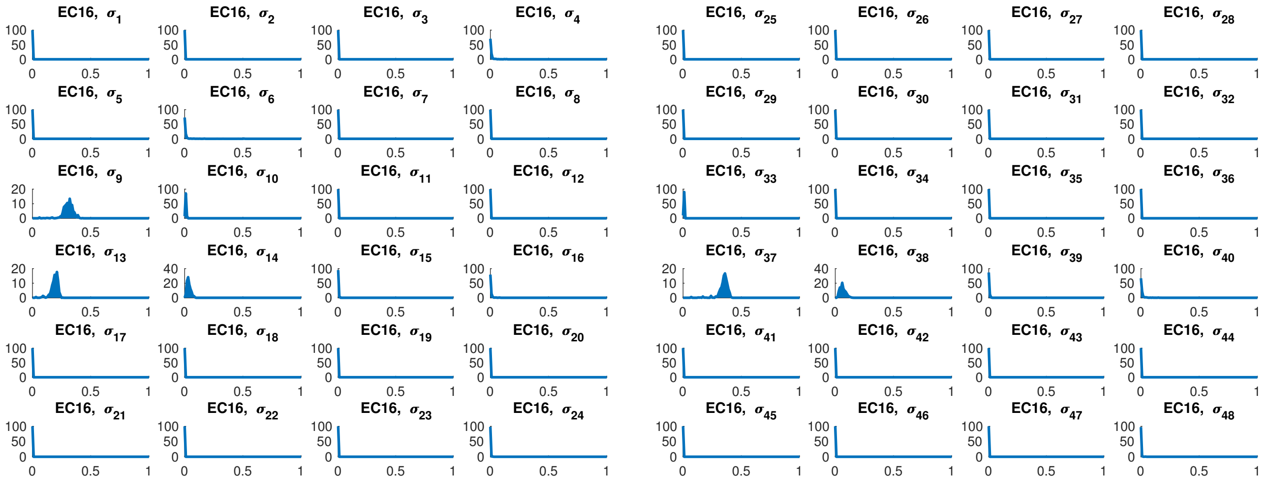

(i)

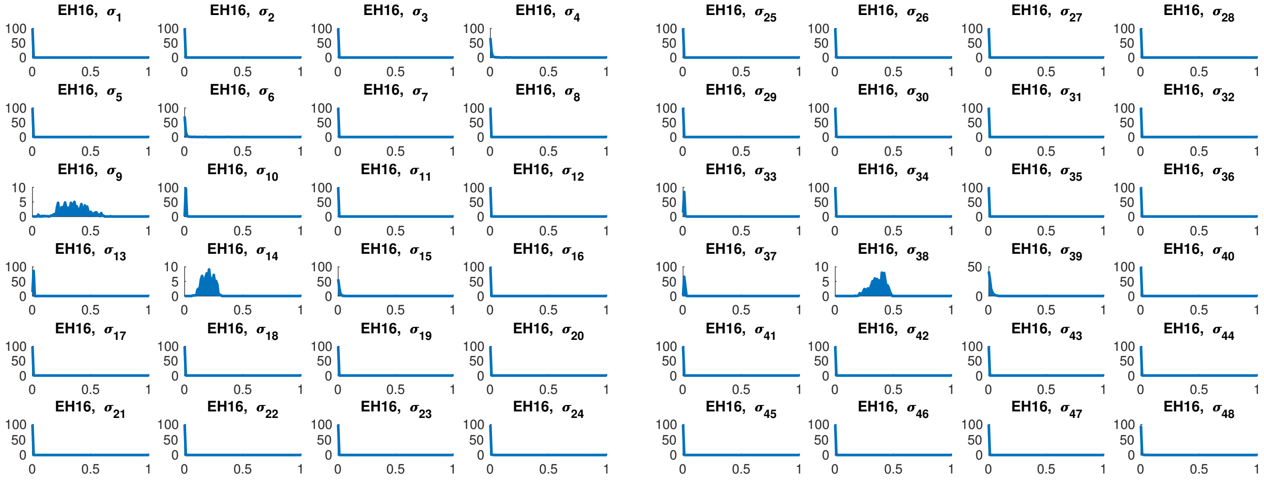

(ii)

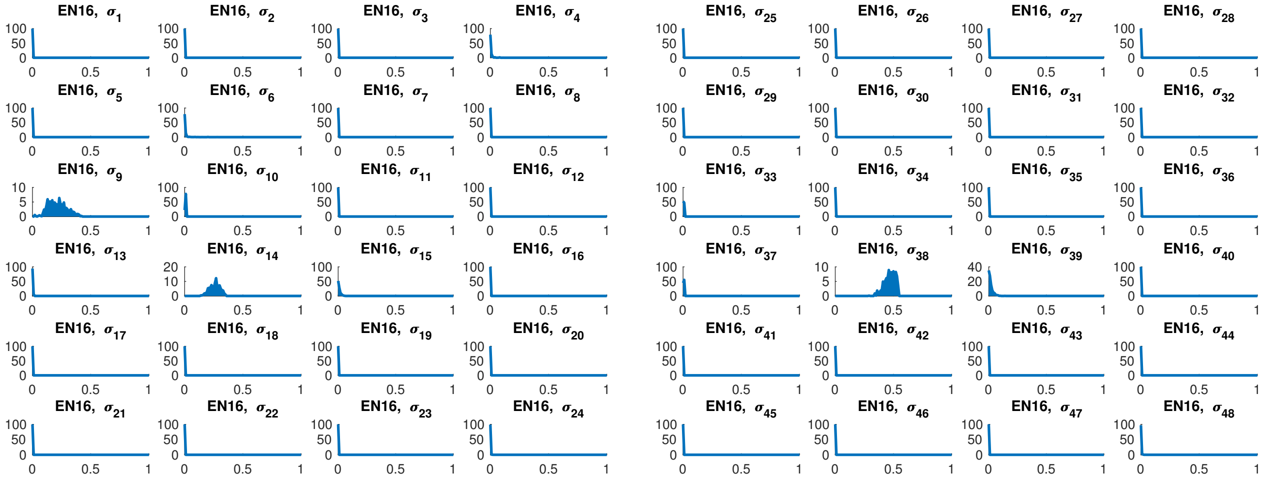

(iii)

Figure L: Probabilities $P(\sigma_k)$ for the wild type (i.e., row E16 of Table 1) in open and closed chromatin. (i): probability distribution of the 48 σ_k states in normal chromatin (EC16 of Table 1) for the parameter sets correctly predicting gain/loss for the entire truth table. In the left panel RNAp is not bound to the TATAbox, in the right panel it is. (ii): probabilities of the same configurations for heterozygous su(var)3-9 mutant (i.e., EH16 in Table 1). (iii): probabilities of the same configurations for homozygous su(var)3-9 mutant (i.e., EN16 in Table 1).

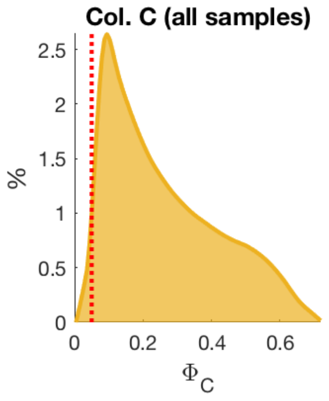

(i)

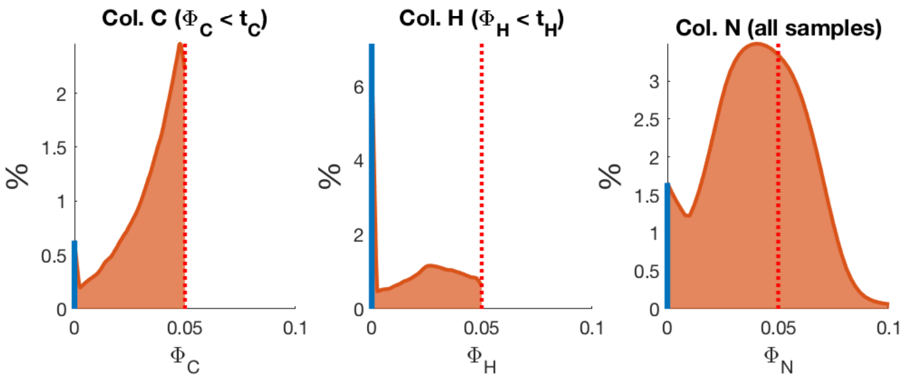

(ii)

Figure M: Prediction error distance. (i): For a non-optimized batch of samples, the distance Φ^C is shown. Only a small fraction of the samples lies below a threshold $\tau = 0.05$. (ii): After tuning the epigenetic parameters, in correspondence of the set of samples ($\sim 2 \cdot 10^5$) which satisfy $\Phi^C < 0.05$ and $\Phi^H < 0.05$, more than 50% of the samples also satisfy $\Phi^N < 0.05$. The blue bars at distance 0 correspond to the subset which fulfills all interval constraints of Table 1 ($\sim 10^3$ samples).
